# *In silico* Analyses of Skin and Peripheral Blood Transcriptional Data in Cutaneous Lupus Reveals CCR2-A Novel Potential Therapeutic Target

**DOI:** 10.3389/fimmu.2019.00640

**Published:** 2019-03-29

**Authors:** Rama Dey-Rao, Animesh A. Sinha

**Affiliations:** Department of Dermatology, Jacobs School of Medicine and Biomedical Sciences, University at Buffalo, Buffalo, NY, United States

**Keywords:** CCLE, cutaneous lupus, skin, autoimmune, therapeutic, bioinformatics, expression profiles

## Abstract

Cutaneous lesions feature prominently in lupus erythematosus (LE). Yet lupus and its cutaneous manifestations exhibit extraordinary clinical heterogeneity, making it imperative to stratify patients with varying organ involvement based on molecular criteria that may be of clinical value. We conducted several *in silico* bioinformatics-based analyses integrating chronic cutaneous lupus erythematosus (CCLE)-skin and blood expression profiles to provide novel insights into disease mechanisms and potential future therapy. In addition to substantiating well-known prominent apoptosis and interferon related response in both tissue environments, the overrepresentation of GO categories in the datasets, in the context of existing literature, led us to model a “disease road-map” demonstrating a coordinated orchestration of the autoimmune response in CCLE reflected in three phases: (1) initiation, (2) amplification, and (3) target damage in skin. Within this framework, we undertook *in silico* interactome analyses to identify significantly “over-connected” genes that are potential key functional players in the metabolic reprogramming associated with skin pathology in CCLE. Furthermore, overlapping and distinct transcriptional “hot spots” within CCLE skin and blood expression profiles mapping to specified chromosomal locations offer selected targets for identifying disease-risk genes. Lastly, we used a novel *in silico* approach to prioritize the receptor protein CCR2, whose expression level in CCLE tissues was validated by qPCR analysis, and suggest it as a drug target for use in future potential CCLE therapy.

## Introduction

Lupus erythematosus (LE) is a complex (1) autoimmune disease that spans a tremendous variety of forms, degrees, and phenotype expressions ranging from relatively well-defined cutaneous manifestations to a rapidly progressive, lethal, multi-organ involvement in the systemic disease (SLE) (2, 3). Cutaneous features represent a major and medically significant component of LE and are evidenced as a wide range of clinical manifestations, some of which are disfiguring and debilitating. CCLE, which encompasses discoid lupus erythematosus (DLE), represents the most common category of cutaneous lupus erythematosus (CLE). While it is clear that CLE pathogenesis is multifactorial, with distinct roles for genetic, environmental, immunologic as well as epigenetic factors, large gaps in knowledge remain regarding the exact causes, mechanisms and biological interactions leading to the development of autoimmune attack on the skin. A number of trigger factors have been reported to influence the course and prognosis of cutaneous lupus including, UV light, medication, and smoking. Additional factors such as race, sex, age of onset and genetic predisposition, among others also impact disease manifestation (4–9). Although susceptibility to CCLE has been linked to genes within the HLA (HLA-DQ and/or DR), as well as outside the HLA region (*HSP70-1, TNF–*α*/*β*, C4* null, *C2, IL-1*, and *IL10*) (10–19) the molecular and genetic basis of disease initiation, progression, and response to treatment is poorly understood.

In large part due to the gap in knowledge regarding disease susceptibility and pathomechanisms, the current therapeutic armamentum in cutaneous LE does not include any approved systemic drugs (20) and needs to be empirically determined for individual patients. Thus, an enhanced understanding of the molecular and genetic basis of disease is a requisite to advance the search for novel therapeutic targets, particularly those that are more targeted and even personalized.

In the present study, gene enrichment analyses of the CCLE skin and blood transcriptional datasets were conducted to illuminate shared, over-represented, disease-related pathways and processes across both tissue environments. Our findings corroborate the involvement of a generalized immune dysregulation and altered apoptosis as central drivers of the cutaneous lupus phenotypes (21, 22). Through our integrative analytical approach we propose a detailed “disease road-map” tethered upon functional enrichments that illuminate a potential orchestration from initiation, amplification to tissue targeting in skin as linked steps in the autoimmune response of CCLE. This framework allowed us to assign existing and emerging therapies within the “disease road-map” based on mechanism of action of specific medications. *In silico* based interactome analysis identified 3 “over-connected” genes as potential key functional players in the metabolic reprogramming associated with skin pathology in CCLE. Subsequently, drug target analyses allowed us to narrow the search and prioritize *CCR2* as the druggable target receptor that needs further research to test viability in potential future disease therapy.

## Materials and Methods

Recruitment of CCLE/CLE (more specifically the most common subtype, discoid LE DLE), age- and sex-matched patients, and healthy control individuals, tissue procurement and handling has been described in detail along with IRB approval number, consent, demographic data and raw data in our earlier reports (21–24). None of the patients were positive for ANA or met any criteria for SLE. No systemic or topical medications had been used by any of the patients for 2 months prior to sampling. The procedures for blood and tissue handling, peripheral blood mononuclear cell extractions, total RNA preparation, cDNA synthesis and microarray processing have also been described previously (21, 22, 25, 26). The transcriptional data analyzed was from skin of 6 lesional and 4 non-lesional biopsies from patients with CCLE and blood from 3 CCLE patients and 3 healthy controls. There was an overlap of 2 patients (1008 and 1009) between blood and skin analysis. The range of sample size reflects the limited human samples that were available for the rare autoimmune disorder CCLE/DLE.

### Differentially Expressed Genes (DEGs)

For the present study, we re-analyzed the CCLE-blood expression dataset to define a new DEGs list based on statistical criteria identical to our previously published CCLE-skin (21) thus allowing a more direct comparison. Briefly, we controlled the *p*-value at ≤ 0.05 and fold change (FC) ≥ ±1.1 and identified 783 non-redundant CCLE- peripheral blood DEGs (337 UP and 446 DOWN), that distinguished patients from healthy controls. The low FC cut off was chosen for this study because: **(**1) minimal expression differences may be biologically significant (27) and **(**2) it allowed us to start with a larger pool of significant genes from both transcriptional profiles with which to perform downstream statistical analyses with high stringencies, in order to streamline our search to only “over-connected” DEGs.

Bioinformatics tools in DAVID (28), (https://david.ncifcrf.gov/) and MetaCore™ v6.21 (Thomson Reuters, St Joseph, MI) were used (http://www.genego.com) to analyze and compare the two gene expression profiles (29). Unique gene symbols from the DEGs lists were mapped to “network objects” and used to probe the MetaCore database (metabase) (30). Using the disease terms “lupus” or “cutaneous lupus” we were able to generate and analyze several disease–associated canonical pathway maps followed by similar investigations in the Gene Ontology (GO) and KEGG databases. This allowed us to dissect each known overlapping disease-related biological pathway/process between the two tissue environments.

We compiled lists of interferon (IFN) inducible genes and those linked to apoptosis related pathways as described earlier (22). We also consolidated one hundred and five *potential* SLE susceptibility loci from genome–wide association studies (GWAS) recorded (31–44) in the National Human Genome Research Institute (NHGRI-EBI) catalog (http://www.ebi.ac.uk/gwas/search?query=lupus) and the SLEGEN study as well as susceptibility loci for CLE in a recent GWAS study (16) for comparison.

The DEG lists were analyzed for their chromosomal enrichment by leveraging the gene expression data to detect regions of the chromosomes with a statistically significant proportion of DEGs (called transcriptional “hot spots”) (45–49). We used DNA–Chip Analyzer (dCHIP) (www.dchip.org) for the purpose of gene mapping using the “genome” tool with masked duplicate probe sets. *P-value* ≤ 0.001 was calculated for all stretches of chromosomes (“hot spots”) that contained ≥ 5 DEGs (CCLE-blood DEGs used for this analysis) (50). We overlaid a similar map generated from our previous site-matched lesional vs. non-lesional skin analysis (from CCLE patients) (21) on the CCLE –blood chromosomal map. We explored the overlapping and unique genes that were significantly associated or not with systemic or cutaneous disease in previous gene expression and GWAS studies.

### Quantifying Gene Expression Using RT-qPCR

Total RNA was isolated as described previously (22, 26) using TRIzol reagent (Invitrogen, San Diego, CA, USA) per manufacturer's protocol and treated with DNase, purified and quantified by Nanodrop ND-1000. We used reverse transcription quantitative real-time PCR (RT-qPCR) to quantify gene expression from peripheral blood of an entirely different set of 5 CCLE patients (LE 1013, 1014, 1016, 1017, and 1018) and 5 healthy controls (CR, 221, 220, 231, 1042, and 1032). cDNA was synthesized from 400 ng total RNA using Promega Kit (Promega Corp., Madison, WI, USA) and quantitative real-time PCR was subsequently carried out using the FastStart Universal SYBR Green Master (ROX) from Roche (Roche Diagnostics,Mannheim, Germany) according to carefully standardized protocols. Intron-spanning primers were designed using Primer3 v. 4.0.0 (http://bioinfo.ut.ee/primer3/) for the following selected DEGs: *CCR2, IFI30, OAS1, OAS2, STAT1 TNFAIP3, ERBB3*, and *FGFR2*, based on published human gene sequences in the Ensemble Genome Browser (http://useast.ensembl.org/index.html). Primers were checked with a BLAT search (http://genome.ucsc.edu/cgi-bin/hgBlat). Amplicons were designed to be < 150 bp in most cases (Table 1). Duplicate experiments were run for the 5 biological replicates in each of the patient and control group. The resulting qPCR cycle times (*C*_t_) were normalized against the β-actin (*ACTB*) housekeeping gene to obtain Δ*C*_t_. Fold changes in expression were calculated using the 2^−ΔΔCt^ method (51) relative to one control sample taken as unity (**Figure 6**). The fold changes in gene expression obtained by qPCR were compared to those observed by microarray analysis.

**Table 1:** Quantifying gene expression.

**#**	**Gene symbol**	**Forward primer sequence**	**Reverse primer sequence**	**Prod size**	**Microarray (fold change)**				**qPCR (fold change) (Blood)**			
					**Skin**	***p*-value**	**Blood**	***p*-value**	**Control**	**±S.E.M**	**CCLE/DLE**	**±S.E.M**
1	CCR2^*^	GAGGCATAGGGCAGTGAGAG	GCAATCCTACAGCCAAGAGC	269	1.8	0.031	1.6	0.006	2.64	0.49	5.98	2.19
2	*IFI30*	TGACCATTGTCTGCATGGAA	TCCATGATAGTGTCTGGCGA	100	3.9	0.0006	2	0.027	1.72	0.21	3.02	0.46
3	*OAS1*	CAACTCTGCATCTACTGGACAAAG	AAGTTTCCTGTAGGGTCCGC	125	2.6	0.0001	6	0.001	2.3	0.47	15.42	6.44
4	*OAS2*	TTGACAACCGTCCTGGAAAA	GTAAGCAGCTCCAGGGCATA	121	2.6	0.007	1.9	0.007	1.71	0.17	8.84	3.52
5	STAT1^*^	TCAGAAGTGCTGAGTTGGCA	GTCCACGGAATGAGACCATC	125	4.2	0.002	2.2	0.043	4.05	0.78	9.94	2.34
6	*TNFAIP3*	TCAACTGGTGTCGAGAAGTCC	ACGCCCCACATGTACTGAGA	97	2.1	0.003	1.5	0.048	1.81	0.41	6	3.06
7	*ERBB3*	GAACATTCGCCCAACCTTTA	ACGTGGCCGATTAAGTGTTC	279	−2.3	0.003	−1.2	0.041	0.29	0.22	0.22	0.07
8	*FGFR2*	CAGAATGGATAAGCCAGCCA	GCTTGAACGTTGGTCTCTGG	95	−2	0.016	−1.3	0.049	0.12	0.12	0.1	0.03

Differential gene expression in peripheral blood measured by RT-qPCR. Intron-spanning primers were chosen for CCR2, IFI30, OAS1, OAS2, STAT1, TNFAIP3, ERBB3, and FGFR2, which are dysregulated genes within the CCLE skin and blood signatures. Q-PCR data is normalized to the housekeeping gene β-actin (ACTB). Fold changes in expression (with S.E.M) are calculated using the 2^−ΔΔCt^ method relative to one sample of the control taken as unity and represent duplicate runs of 5 biological replicates each of CCLE (DLE)-patients and healthy controls. Fold change in expression (between case and control blood samples) of CCR2 and STAT1 trended toward significance with p-values = 0.055 and 0.053, respectively (^*^starred). Gene expression values from the microarray data are included as fold change in gene expression in skin and blood associated with p ≤ 0.05. CCLE, chronic cutaneous lupus erythematosus/Discoid lupus erythematosus; S.E.M., standard error of the mean (see also **Figure 6**).

### Interactome and Drug Target Analysis

We started out with the broadest pool of statistically significant genes, and subsequently employ downstream bioinformatics-based statistical tools with high stringency to assign disease relevance. To go beyond simply cataloging disease-related molecules we subjected our DEGs from CCLE skin and blood to an interactome analysis by protein function” (52). The relative connectivity of a gene (encoded protein) mirrors its functional significance to the disease under investigation (53) and is calculated by the number of interactions between the experimental gene with the genes on the experimental list normalized to the number of interactions it has with all genes in the human database (in MetaCore). The ranking of importance is related to the “knowledge based” analysis that takes into consideration experimental DEGs in the context of their known interactions in complex gene/protein and molecular networks. The localization of receptors in membranes that allows the extracellular domains to be targeted by specific ligands and drugs was key to narrowing the search to the “over-connected” 3 receptors that were shared between the skin and blood transcriptional profiles. Finally, we were able to prioritize one DEG *CCR2* based on several analytical criteria discussed above. Network generating algorithms in MetaCore were based on auto-expand by one interaction including both up- and downstream reactions. We further categorized the genes by their association with SLE-genes (either expressed or as susceptibility loci). A “drug target analysis” was performed via MetaCore along with a literature search on all three receptor proteins, to discover novel drug/target combinations that offer the best potential to be used in the treatment of CLE.

## Results

### Differentially Expressed Genes (DEGs) and Ontology Enrichment Analysis

**Figure 1:**
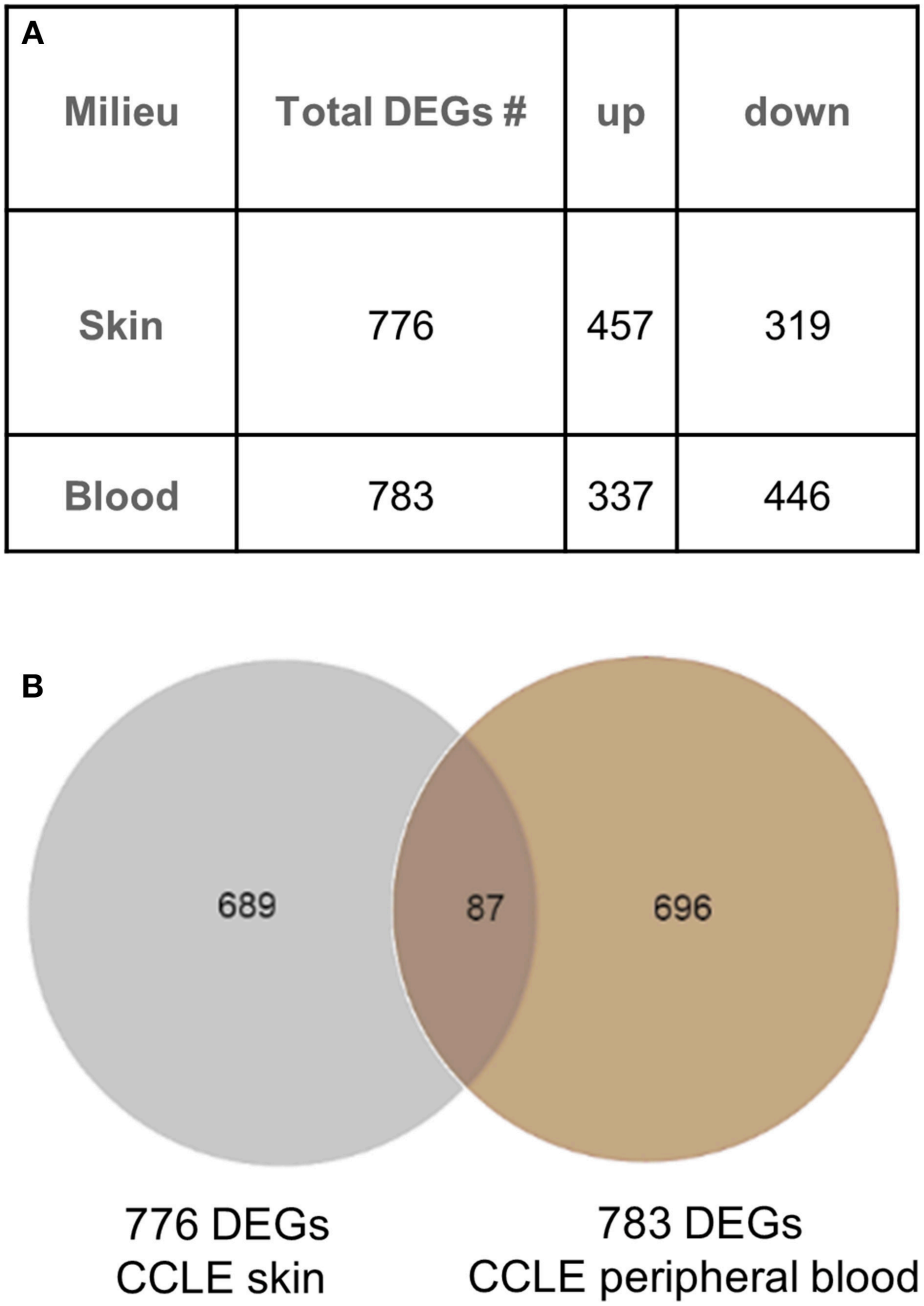
Gene expression analysis in CCLE blood and lesional skin. **(A)** Differentially expressed genes (DEGs) were generated from blood (CCLE vs. healthy control) and skin (lesional vs. non-lesional) of CCLE patients and healthy controls that were able to distinguish between compared phenotypes via hierarchical clustering. **(B)** An overlap of 11% (87 DEGs) between the two lists is shown by the Venn diagram. The majority of genes in both lists are distinct to the respective environment (skin and blood) from which they are generated.

We generated a blood gene expression profile of 783 non-redundant DEGs (Supplementary Table 1) for comparison with the skin profile (776 DEGs) described previously (21). Both lists were generated using the same fold change and *p*-value cut off (Figure 1A). Of the 87 DEGs (Figure 1B) common between the blood and skin transcriptional profiles, 65 were dysregulated in the same direction in both tissues (upregulated = UP or down-regulated = DOWN) and 22 were in the opposite direction (Supplementary Table 2).

### Pathways and Processes Based Enrichment Analyses

**Figure 2:**
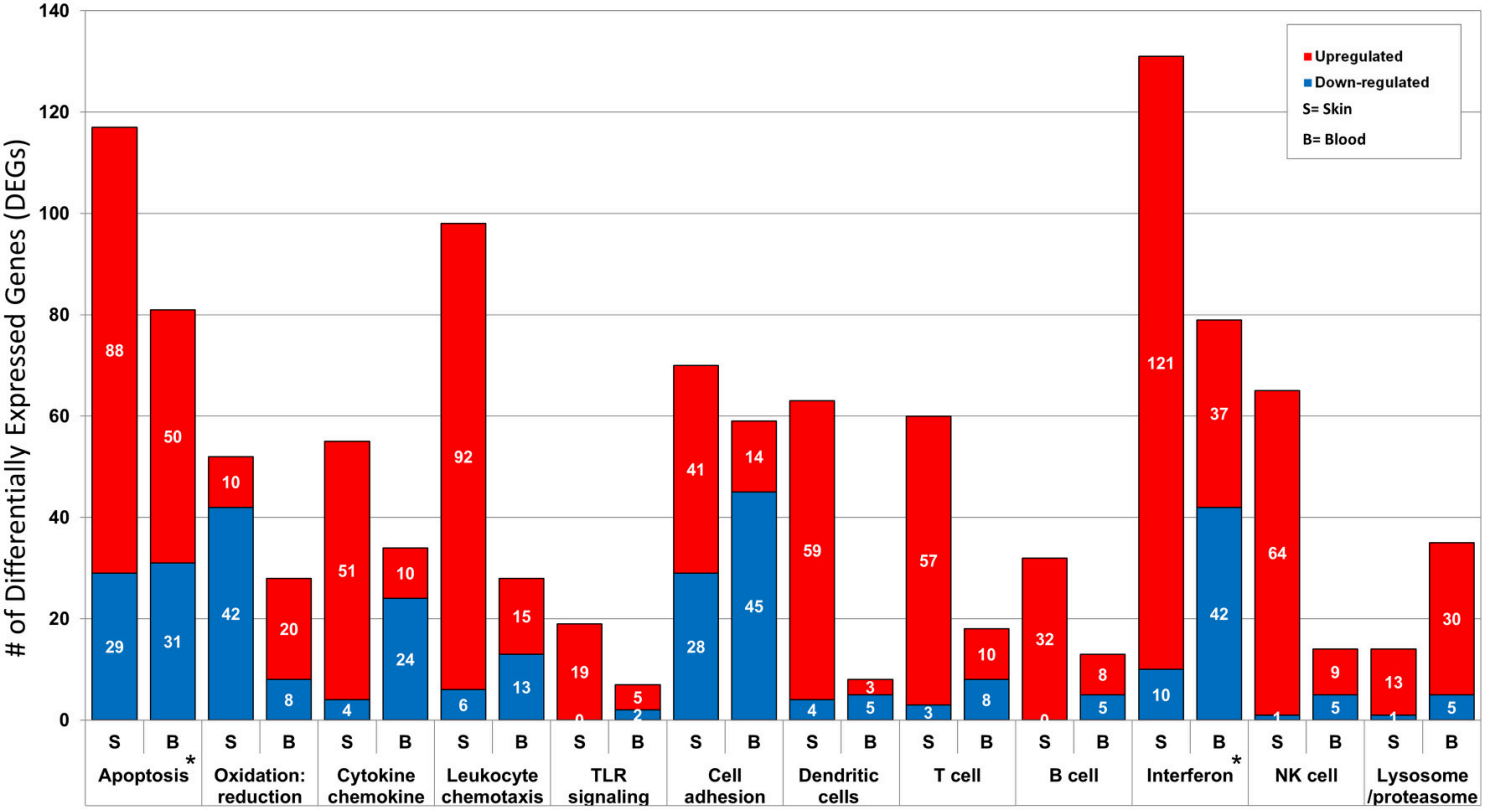
Functional annotation and pathway analysis of the DEGs from CCLE-blood and lesional skin. The two transcriptional profiles reveal several enriched disease-related Gene Ontology (GO) biological processes. We break down the number of DEGs (up- and down-regulated) included in some of the shared disease related processes such as: apoptosis, oxidation: reduction, cytokine chemokine, leukocyte chemotaxis, TLR signaling, cell adhesion, dendritic cells, T cell, B cell, type I interferon, NK cells and lysosome/proteasome. The most prominent signatures for interferon and apoptosis in both profiles are marked with a star (^*^).

Ontology enrichment analysis was processed through two different analytical platforms (DAVID and MetaCore) to identify enriched disease-related biological functions, pathways and processes. We uncovered prominent apoptotic and type I interferon (IFN) signatures in both tissue environments. The overall number of DEGs in the activated immune response related pathways and processes are consistently higher in lesional skin than in peripheral blood. However, processes associated with lysome/proteasome related breakdown were more pronounced in peripheral blood than in skin. The differences and similarities in the two tissue environments forms the basis of our present analysis aimed at elucidating specific local and systemic disturbances linked to pathomechanisms related to CCLE (Figure 2).

**Figure 3:**
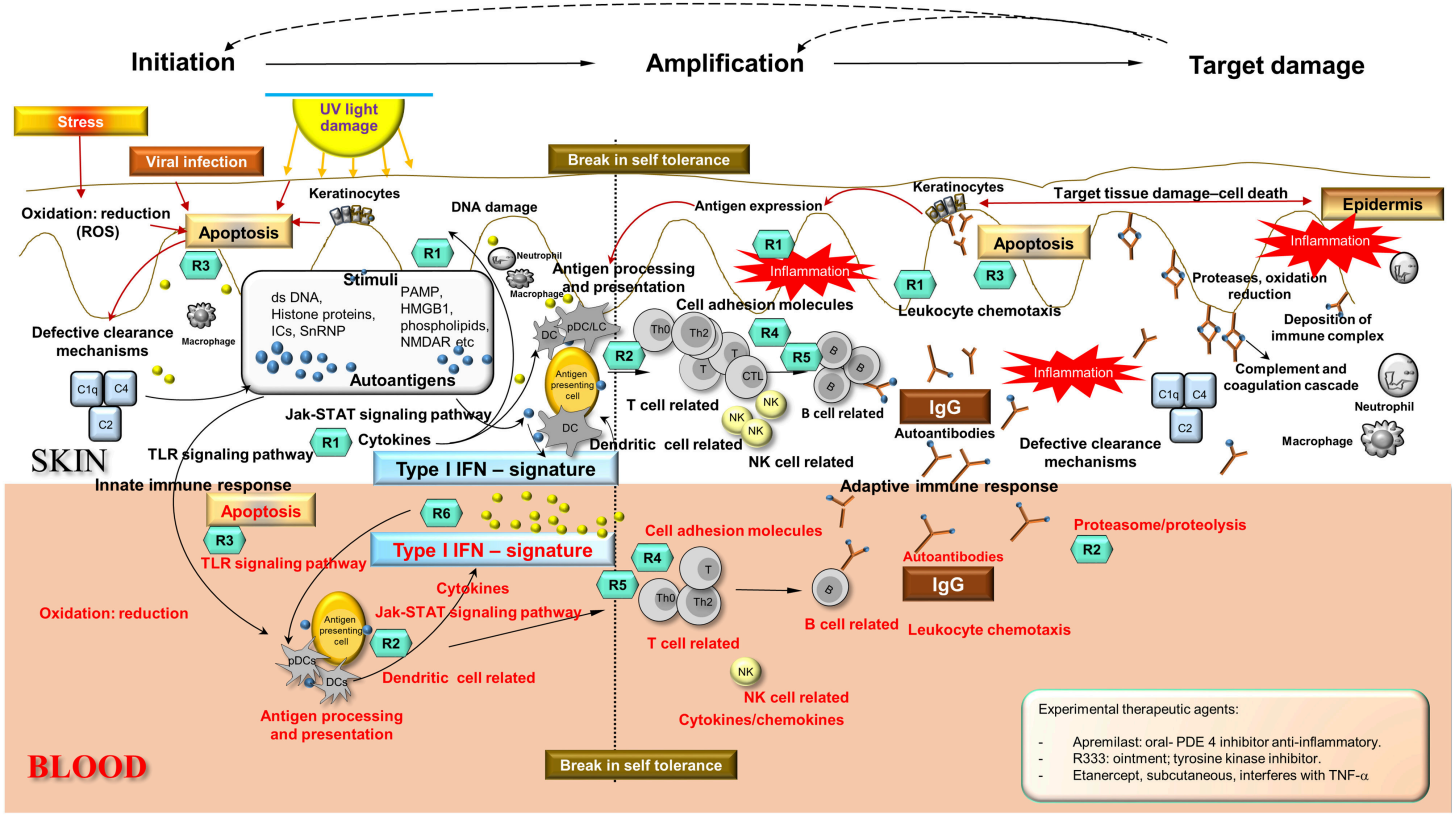
Disease Road Map. Enriched disease-related pathways and processes found in skin and blood profiles underlie three distinct phases in the autoimmune response in CCLE: (i) initiation, (ii) amplification of the immune response, and (iii) target damage in the skin, that are not mutually exclusive. The shared disease-related processes over-represented in skin and blood are as follows: apoptosis, oxidation reduction, cell adhesion, cytokine chemokine activity, leukocyte chemotaxis, NK cell, TLR signaling, dendritic cells, T cell, B cell activation, Interferon signature, lysosome and proteasome, antigen processing and presentation, complement cascade among others (see also Figure 2). Potential molecular targets of some of the existing and emerging drugs R (*n*) have been identified in the schematic according to mechanism of action presently used for CLE/DLE treatment. Some experimental drugs have also been included (see inserted box). Drugs used in CLE/DLE treatment R(*n*) (teal hexagons): *Topical agents:* R1 = corticosteroids fluocinonide, R-salbutamol sulfate (anti-inflammatory, acting upon cytokines and leukocytes) *Systemic agents:* R1 = glucocorticoids, R2 = hydroxychloroquine (antimalarial) (acting upon MHC presentation and lysosome pH, anti-inflammatory), R3 = thalidomide, lenalidomide, CC 11050 (TNF-α inhibitors, apoptosis), R4 = methotrexate MTX (acts on T cell proliferation), R5 = pimecrolimus (calcineurin inhibitor, down-regulating T cell activity) TRX-1, AMG 557 (humanized mAbs act as T cell regulators), R6 = humanized mAbs AMG 811 (anti-IFNα) and sirukumab (acts on pro-inflammatory cytokines such as IL-6). Ds, double stranded; UV, ultraviolet; PAMP, pathogen–associated molecular pattern; HMGB1, high mobility group protein B1; ICs, nucleic acid containing immune complexes; NMDAR, N-methyl-D-aspartate receptor; C1q, complement component 1; q subcomponent; C2, complement component 2; C4, complement component 4; DC, dendritic cells; pDC, plasmacytoid DC; LC, Langerhans cells; T, T cells; B, B cells; Th, T helper cells; Th0, T helper cells 0; CTL, cytotoxic T lymphocytes; IgG, immunoglobulin G; NK, natural killer cells; IFN, interferon; TLR, toll-like receptor; PDE4, phosphodiesterase-4; TNF, tumor necrosis factor.

Integration and synthesis of CCLE blood and skin transcriptional data in the context of existing literature regarding SLE/CCLE-related processes suggests a coordinated orchestration of the autoimmune response leading to organ-specific tissue damage in the skin that can be viewed in 3 phases: (1) initiation, (2) amplification of immune response, and (3) target damage in skin (Figure 3).

### Identification of Risk Loci

**Figure 4:**
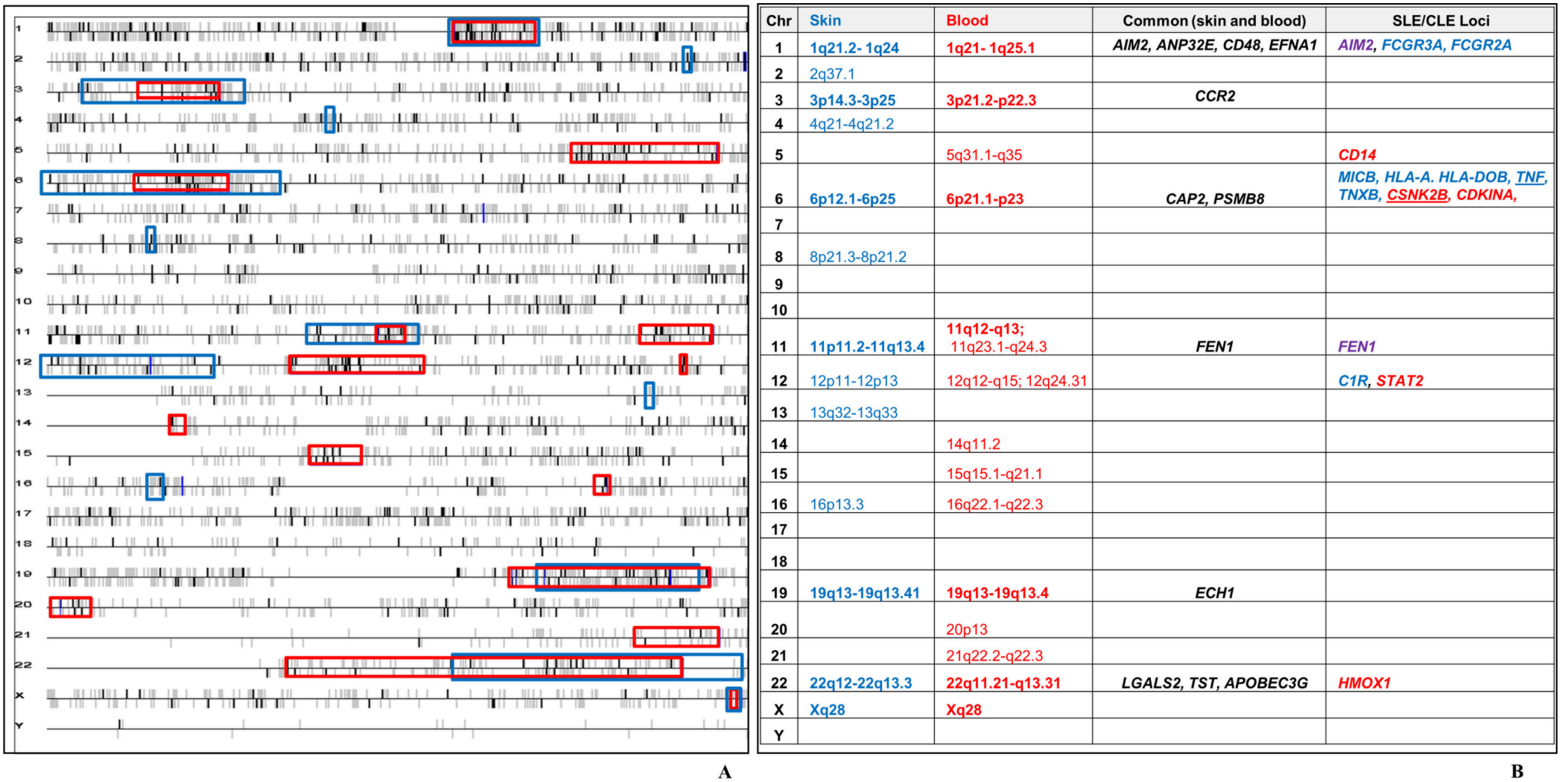
Chromosomal locations of transcriptional “hot spots” found in CCLE-associated blood and lesional skin signatures. **(A)** CCLE blood gene expression data is leveraged to identify transcriptional “hot-spots” in chromosomes where DEGs map with statistically increased frequency. Each horizontal line corresponds to one chromosome. Chromosomal locations of CCLE blood molecular signature are colored in bold black vertical bars vs. the non-differentially expressed genes which are gray. The vertical bars above and below the horizontal lines represent genes either on the forward or reverse strand. Sixteen significant stretches at *p*-value ≤ 0.001 are considered to be transcriptionally active “hot spots” using the CCLE blood profile (red boxes). Thirteen “hot spots” associated with CCLE lesional skin signature (blue boxes) from our previous study, have been overlaid on the blood chromosomal map revealing seven regions of overlap (bold). **(B)** Twelve genes in the overlapping “hot spots” (including ***CCR2***) were shared between the skin and blood profiles. The seven overlapping “hot spots” also include 14 DEGs from skin (blue), blood (red) and both milieus (purple), which have been previously reported as potential disease loci in SLE. *TNF* is a skin-DEG that is previously described as a putative disease locus for both SLE as well as CLE. *CSNK2B* is a blood-DEG that has been described as a disease locus in CLE.

We leveraged the blood transcriptional profile to locate genomic regions in which the 783 CCLE-DEGs cluster more frequently than would be expected by chance.(47, 49) 16 transcriptional “hot spots” were identified on chromosomes 1, 3, 5, 6, 11 (2), 12 (2), 14, 15, 16, 19, 20, 21, 22, and X, harboring 177 CCLE-blood DEGs. We had previously described 13 CCLE-skin specific “hot spots” on chromosomes (21). Seven transcriptionally active regions (chromosome 1, 3, 6, 11, 19, 22, and X) overlapped between skin and blood profiles (Figure 4A). Twelve dysregulated genes including *AIM2, ANP32E, CD48, EFNA1, CCR2, CAP2, PSMB8, FEN1, ECH1, LGALS2, TST*, and *APOBEC3G* were common to skin and blood within the overlapping “hot spots” (Figure 4B). See Supplementary Table 3 for details on the 177 DEGs contained within the sixteen CCLE-blood “hot spots.”

### Identification of a Novel Therapeutic Target

Using our transcriptional datasets, CCLE-specific gene regulation was evaluated through enrichment by “protein function” revealing a significantly high number of ligands, proteases, receptors and other protein classes (Supplementary Table 4). Overall interaction topology reveals a high level of incoming and outgoing connections within the CCLE-experimental dataset as well as to and from the CCLE-datasets to the metabase (Supplementary Table 5).

**Figure 5:**
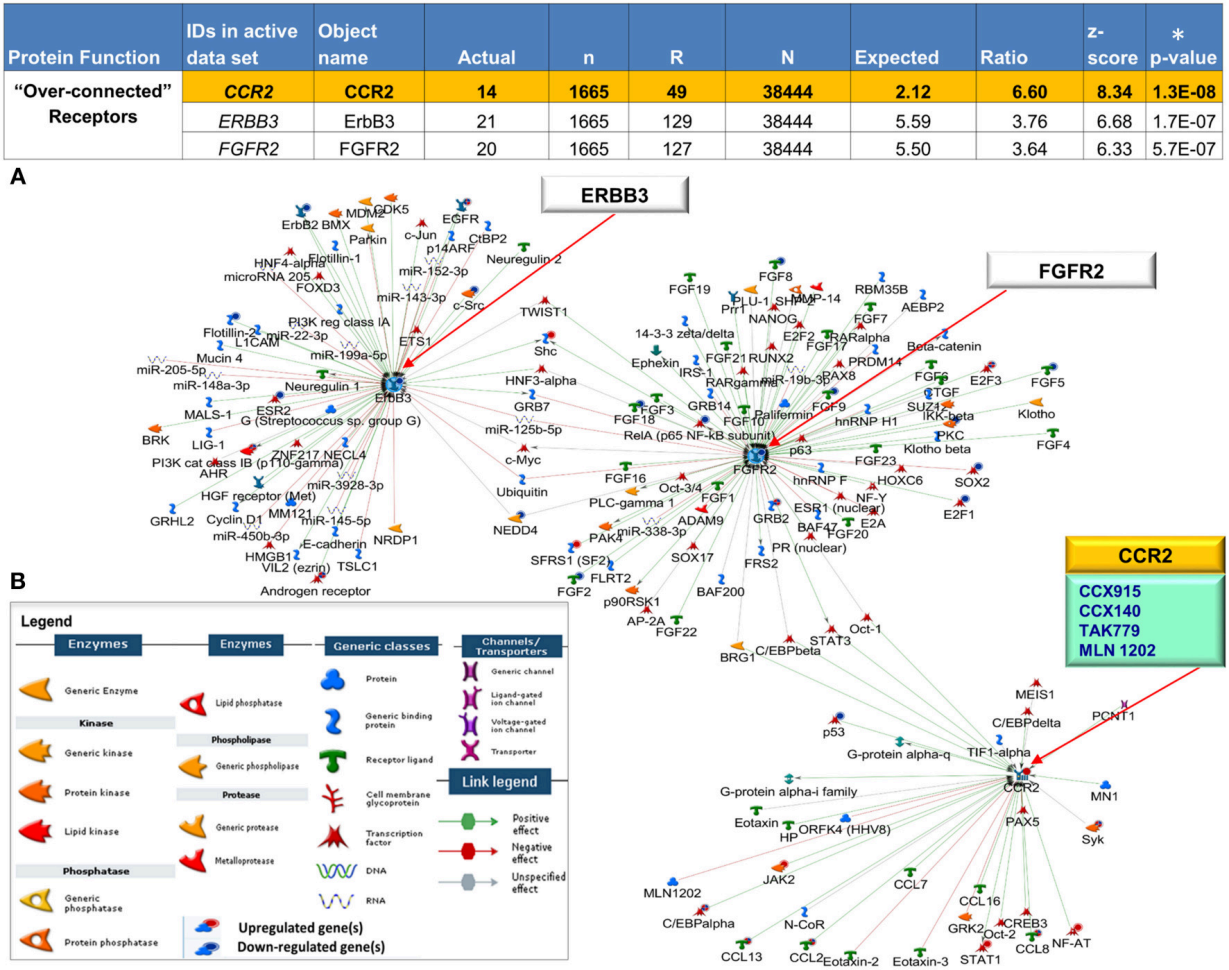
Interactive receptor proteins CCR2, ERBB3 and FGFR2. **(A)** We focused on three receptor proteins (CCR2, ERBB3, and FGFR2) that were considered “over-connected” when the number of observed interactions (actual) was greater than the number of expected interactions accompanied by (significant) low *p*-values (^*^) and high z-scores **(B)** Algorithms in MetaCore are used to generate networks using the expand by single interaction in both upstream and downstream directions. Interactions within the network reveal all 3 receptors as central hubs connected to each other as well as with a large number of curated interactions with both positive (green) and negative effect (red) to and from (arrow direction) several objects in the CCLE-skin profile as well the larger human proteome database. The individual CCLE-DEGs and proteins in the metabase are representated as nodes of different shapes (see legend) with connections/interactions to one another. Drug target analysis revealed 4 drugs (teal box) that target one of the receptor proteins CCR2, (yellow). All 4 drugs are currently being used in the clinic to treat other closely related diseases (see Supplementary Table 7). Explanation of columns: Actual, number of network objects in the activated signatures which interact with the chosen object; *n*, number of network objects in experimental datasets; *R*, number of network objects in the activated background list which interact with the chosen object; *N*, total number of protein-based objects in the activated background list; Expected, mean of hypergeometric distribution; Ratio, connectivity ratio (Actual/Expected); z-score, (Actual-Expected)/(standard deviation); *p*-value: probability to have the value of Actual by chance under null hypothesis of no over-connectivity. Star (^*^) denotes significant *p*-values.

We leveraged our expression dataset to search for key drivers of mechanisms underlying CCLE pathogenesis. We reveal 44 DEGs shared between blood and skin that are statistically “over-connected” to objects within the two CCLE datasets as well as the larger human database in MetaCore (Supplementary Table 6). Three (*CCR2, ERBB3*, and *FGFR2)* of the seven “over-connected” shared DEGs coding for receptor proteins (localized at the cell membrane) were selected for further investigations. All three were found to be interactive hubs by interactome and network analysis (Figure 5). A literature- and Metabase- search with the term “discoid lupus erythematosus” allowed an investigation into existing as well as emerging therapies in CLE/DLE. A drug analysis on all three receptors revealed CCR2 to be targeted by drugs that are currently in clinical trials for treatment of other related diseases. see Supplementary Table 7 for a list of existing, emerging, and experimental as well as the newly proposed therapeutic agents, along with relevant literature and source documentation.

### Validation Studies

**Figure 6:**
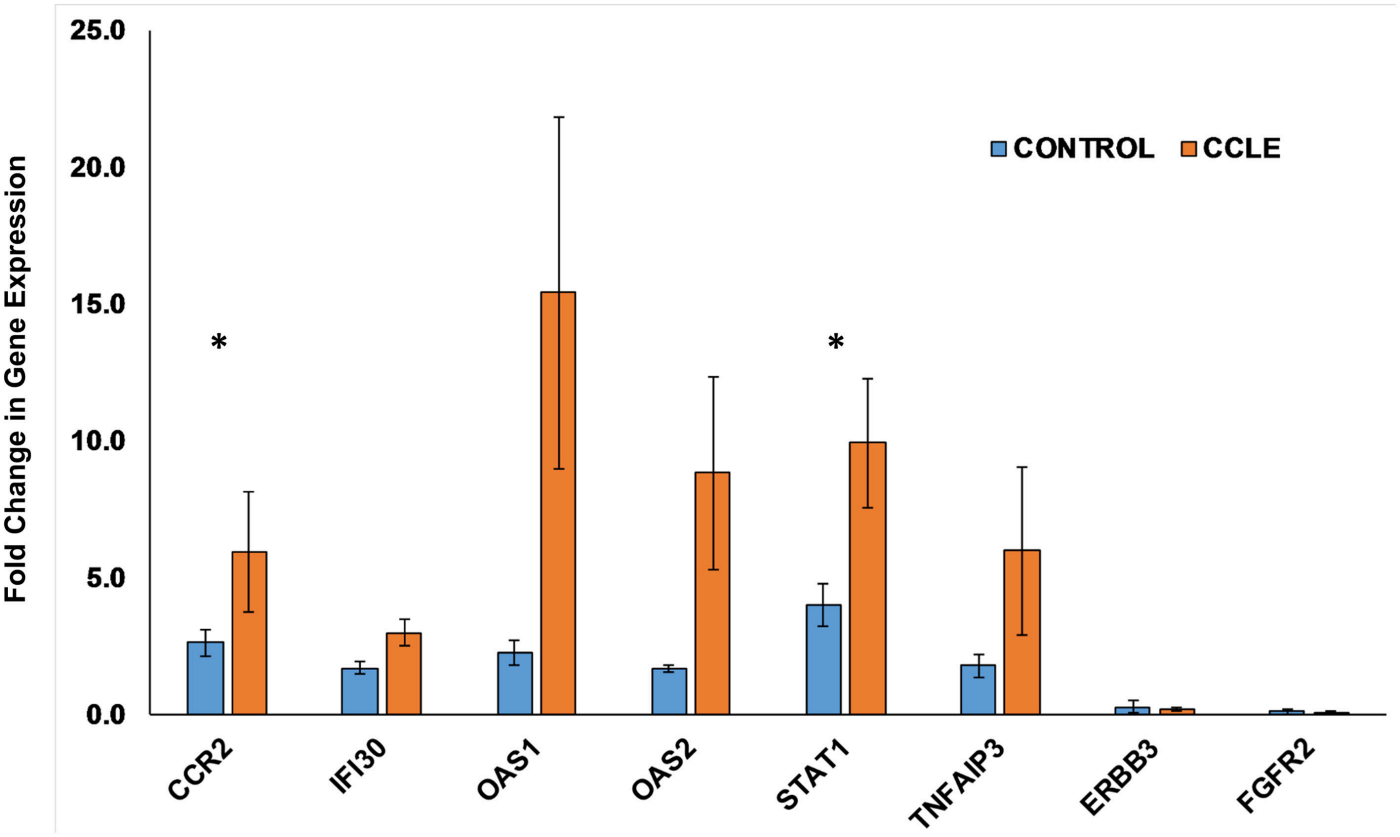
Quantifying gene expression by RT-qPCR. Gene expression for the eight DEGs: *CCR2, IFI30, OAS1, OAS2, STAT1, TNFAIP3, ERBB3*, and *FGFR2*, within both the CCLE skin and blood signatures were quantified using RT-qPCR. Data is normalized to the housekeeping gene β-actin (*ACTB*). Fold change in gene expression in peripheral blood (presented as bar graphs with S.E.M) is calculated using the 2^−ΔΔCt^ method and represent duplicate runs of 5 biological replicates each of CCLE (DLE)-patients and healthy control individuals. Fold change in expression (between case and controls) of *CCR2* and *STAT1* trended toward significant (^*^p-value = 0.055 and 0.053, respectively). CCLE, chronic cutaneous lupus erythematosus/discoid lupus erythematosus; S.E.M., standard error of the mean.

We further evaluated the expression of 8 genes shown to be dysregulated by microarray analysis in CCLE/DLE by RT-qPCR (qPCR) (Table 1 and Figure 6). Included among the 8 was *CCR2*, which we propose as a potential drug target in CCLE therapy. The qPCR experiments demonstrate a relative level of gene expression consistent with our microarray data for all eight DEGs. Genes such as *CCR2, IFI30, OAS1, OAS2*, and *STAT1* and *TNFAIP3* are over-expressed in CCLE/DLE patients as compared to healthy controls. While ERBB3 and FGFR2 exhibited overall low levels of expression, the trend in downward expression (case vs. controls) was also similar to our microarray analysis.

## Discussion

### Development of a Disease “Roadmap”

Our new *in silico* analyses reveal a more enhanced composite of disease pathomechanisms operative in CCLE. In the proposed disease road-map schema (Figure 3), the three phases are not necessarily mutually exclusive, with specific components potentially operative in two, or all three phases across the spectrum. Below, we break down the three broad phases: (1) initiation, (2) amplification, (3) target damage, and dissect the linked disease relevant biological pathway/process (Figure 2) to discuss a model for pathobiology in the context of recognized systemic (blood) and local (skin) disturbances.

#### Initiation

Several well-known trigger factors e.g., UV light, medications, smoking are associated with cutaneous lupus (4, 9, 54–57), all of which could contribute to enhanced autoantigen display to the immune system. Theoretically, this could occur via increased keratinocyte apoptosis, autoantigen translocation to the keratinocyte cell surface, and/or a decreased clearance of apoptotic debris as in SLE (58), causing self-antigens (e.g., nucleosomes) to persist in the extracellular environment, enabling recognition by autoreactive cells (59–65). The skin has been speculated to be the site of autoimmune initiation for both cutaneous and systemic disease in genetically susceptible individuals (56, 57, 66).

### Apoptosis

The potential significance of apoptosis in CLE is underscored by several observations including an increase in the number of UV induced apoptotic keratinocytes found in lesional skin (54, 67). In our analysis, a larger number of dysregulated genes were associated with apoptosis in lesional skin (117 with 75% UP) as compared to peripheral blood (81 with 61% UP), emphasizing enhanced apoptosis (particularly in the skin) as a prominent signature of the disease. Both pro- and anti-apoptotic molecules are dysregulated simultaneously in both environments (21, 22), underscoring the complex and epistatic nature of the apoptotic cascade. It might also indicate redundancies of contributors to apoptosis, perhaps including involvement of diverse cell types (e.g., keratinocytes and lymphocytes) relevant to lupus pathology.

### Oxidation: Reduction

Increased upregulation (71% of the 28 DEGs) in oxidative and nitrosative stress related processes in CCLE peripheral blood demonstrated in our dataset is similar to past observations in both SLE and CLE (68–71), underscoring the possibility of post-translational modification of auto-proteins/peptides, rendering them altered and able to provoke the autoimmune reaction.

### Cytokines, Chemokine, and Leukocyte Chemotaxis

UV radiation is known to induce the production of numerous pro-inflammatory cytokines in the skin and contributes to the marked photosensitivity observed in CCLE (56, 72). These cytokines can promote translocation of intracellular autoantigens to the surface of epidermal keratinocytes (13, 73). We find higher numbers of upregulated DEGs (93%*)* associated with cytokine-chemokine related processes in lesional skin as opposed to blood (70% DOWN), perhaps reflecting an influx of cytokine producing cells at the local site of pathology. This may be particularly important as these mediators are short lived and local effects could be tissue specific and different from systemic effects (74).

### Pattern Recognition Receptor (PRR) Signaling

We discovered 19 genes (100% UP) related to PRR to be activated in lesional skin, as opposed to 7 (71% UP) in peripheral blood. Upregulation of TLR-4 expression was previously demonstrated in affected tissue of CLE patients (75). PRRs participate in the recognition of both extrinsic and endogenous danger-associated conserved molecular patterns, and thus bridge innate and adaptive immune responses.

#### Amplification of Immune Response

CCLE is characterized by an increase in local mediators of inflammation in the skin. These effects in turn induce changes which include induction of adhesion molecules that are needed for the migration and sequestration of activated lymphocytes and leukocytes to the skin (76). Accumulating apoptotic debris may also bolster autoantigen presentation by Langerhans cells (LCs) to T cells (77). Binding of autoantibodies to keratinocytes could then further expose self-antigens and promote cytokine release to continue and amplify the autoimmune response. T cell reactivity may in turn promote B cell activation and production of autoantibodies specific to previously sequestered or altered molecules.

#### Cell Adhesion

Progression of lupus is facilitated by the local generation of adhesion molecules and expression of their ligands in inflammatory cells (78, 79). In the present study, we demonstrate nearly equal numbers of dysregulated genes linked to cell adhesion in both skin and blood, but observe 59% UP in lesional skin as compared to 76% DOWN in peripheral blood.

#### Dendritic Cells (DC)

Antigen presentation by *immature* DCs may lead to T cell anergy due to lack of co-stimulation, a mechanism by which certain (self) antigens may evade immune responsiveness (80, 81). We observe much higher numbers of activated genes associated with skin-dendritic cells (94% UP of 63 DEGs), as compared to only 8 DEGs (63% DOWN) in the peripheral blood, underscoring clear patho-mechanistic differences in the two environments.

#### HLA Region

CLE susceptibility (in patients positive for anti-Ro) is linked to the major histocompatibility complex (MHC) on chromosome 6, including genes for human leukocyte antigens (HLA), complement components, and tumor necrosis factor (TNF) (12, 21, 82–84). We found the top enriched KEGG pathway associated with the 87 DEGs overlapping between CCLE-skin and blood profiles to be antigen processing and presentation (Supplementary Table 8) skewed toward activation in both skin (92% UP) and blood (79% UP). Of the twelve DEGs mapping to chromosome 6, *CSNK2B*, and *CDKN1A* are reported as potential CLE-associated susceptibility locus (16) and associated with SLE (85), respectively (Supplementary Table 3). The precise influence of the HLA region on CCLE is not clear as of yet, but is likely related to its well-defined role in antigen presentation and activation/selection of T cells in the pathogenesis of LE (86–91).

### T Cell Response

Our analysis uncovered 60 DEGs (95% UP) in skin and 18 (56% UP) in blood (Figure 2) that are involved in T cell related processes such as -activation, -differentiation, -selection, -proliferation, -receptor signaling and -thymic selection, thus implicating a similarly activated T cell mediated autoimmune reaction in CCLE as well.

### B Cells

Although B cell hyperactivity and the production of autoantibodies in LE appears to be T cell driven (92, 93), breaking of B cell tolerance without the support of T cells has been reported as well (94). Given that 55–75 % of B cell receptors on human immature B cells are self-reactive, maintenance of B cell tolerance is vital for thwarting the production of autoantibodies with potential disease causing specificities (95). We observed 32 DEGs (100% UP) in lesional skin and 13 (62% UP) in blood associated with B cell antigen receptor engagement.

### Interferon

Cytokines play a crucial role in modulating the immune response to foreign and self-antigens, both in the initiation and amplification of the immune response in CCLE. A prominent IFN-α signature in dermal lesions of SLE patients, suggests that the skin acts as a reservoir for IFN producing cells with the ability to promote autoimmunity (96–98). This appears to be a central theme in cutaneous lupus as well (99). In this analysis, we demonstrate a stronger IFN signature in CCLE skin (that is predominantly upregulated) as compared to peripheral blood. This may indicate a potential shift in the engagement of this key cytokine pathway as a pathogenic mediator from the blood to the skin in CCLE patients. Supplementary Figure 1 demonstrates one such over-represented and activated canonical pathway linked to IFN signaling, with more upregulated genes in the skin than from blood. A more comprehensive picture of the role of various IFN family members in disease pathogenesis will emerge as detailed demographics such as disease onset, age, organs involved, and therapy are taken into consideration.

#### Target Damage

The pathology in CLE is one of inflammatory lichenoid reaction in which basal keratinocytes that express surface self-antigens are the chief center of damage (100). Global gene expression data revealing dysregulated apoptosis, inflammation, complement system as well as lysosome and proteasome associated breakdown processes in skin and blood from CCLE patients serves to support the model for the creation, accumulation and presentation of autoantigens to the immune system to be at the heart of the disease (101–103). Dysregulated apoptosis and clearance processes might be significant for both initiating the autoimmune response as well as for the ultimate damage to the skin, demonstrating how various phases of the disease might be mutually non-exclusive.

### Apoptosis/NK Cells and Inflammation

The role of defects in the apoptotic pathway has been discussed previously in the context of disease initiation, but may be relevant for ultimate target damage as well. Multiple apoptotic pathways are involved in both CCLE skin and blood. We observed upregulated markers from both the extrinsic (death receptor related) and intrinsic pathway (stress related) in blood and skin. An understanding of the molecular events that regulate cell death at both the skin and systemic level is essential for clarification of pathogenesis in the disease.

In addition to UV induction of apoptosis, cellular cytotoxic mechanisms involving CTLs, and natural killer (NK) cells have also been implicated in CLE (4, 104–107). Our analysis finds several more NK cell associated genes (65 DEGs, mostly UP) in the CCLE lesional skin than in the blood profile (14 DEGs, mostly UP), with 7 DEGs shared between the two environments. Inflammatory response also included more activated genes in the skin (90% of 144 DEGs UP) than in the blood (78% of 14 DEGs UP).

### Complement

The complement cascade is known to be involved in opsonization of apoptotic cells for efficient clearance by phagocytosis, in the absence of which apoptotic cells may remain longer in the system to stimulate autoantibody production (108). This aberration in the clearing mechanism could be associated with both initiation as well as the target damage stage of the autoimmune reaction. In the present study, we observe many more dysregulated genes related to the complement cascade in lesional skin (such as *C2, C1R, C1QB, C3AR1, C4A /// C4B, CD59, CFB, CFD, ERCC6 /// PGBD3*, and *ITGB2* among others; 89% UP) than in blood (*C4BPB, CFHR3, FANCC PLAU, IGHG1*. and *ITGB2;* 83% DOWN). Another effect of complement activation can be apoptosis through cellular events (109), and could be yet another explanation of the increased apoptosis observed in lesional skin over blood (described earlier).

### Lysosome/Proteasome

Ubiquitin-mediated proteasomal proteolysis constitutes the intracellular protein-degradation apparatus involved in several cellular functions such as cell cycle, cell differentiation, immune and inflammatory response, stress signaling and apoptosis among others. Upregulated genes (*CTSB, CTSC, CTSH, CTSZ*, and *CTSL*) from the cathepsin related protease family, as well as 15 other members of the proteasome family (all involved in proteolysis), were skewed toward the blood than in skin. The evidence supporting enhanced activation of proteolytic markers in peripheral blood over skin of CCLE patients indicates effective processing of peptides in the systemic milieu to enable MHC I and II mediated autoantigen presentation in the disease, which could lead back to amplification of the immune response and serve as a feedback mechanism in the disease.

Overall, our investigations offer a global comprehensive viewpoint of CCLE-associated transcriptional changes that may be particularly relevant for understanding disease mechanisms and identification of biomarkers relevant to disease progression.

### Genetics

We have reported a significant, but not complete overlap between our CCLE expression data and previously reported SLE transcriptional data, indicating that while these two conditions are related, they are also clearly distinct (110). Five of the twelve shared DEGs (described above) in the overlapping CCLE skin and blood “hot spot” regions (*AIM2, CD48, CCR2, FEN1*, and *LGALS2*) also overlap with previously reported SLE associated genes expression or GWAS studies. The remaining seven common DEGs in the two profiles (*ANP32E, EFNA1, CAP2, PSMB8, ECH1, TST*, and *APOBEC3G)* that do not overlap with SLE may thus represent CCLE specific genes. *CSNK2B* (casein kinase 2, beta polypeptide), in the CCLE-blood “hot spot” within the MHC region of chromosome 6, and *APOC1*, mapping to the “hot spot” on chromosome 19 in CCLE-skin have also previously been reported as potential gene markers of CLE (16, 111).

Although the pathogenetic relationship between DLE and SLE is still unclear, our comparative CCLE skin and blood analyses supports the existence of both overlapping as well as distinct genetic susceptibilities and mechanisms relevant to the development of systemic as well as cutaneous LE as discussed previously (4, 21, 22, 110, 112, 113). Furthermore, the DEGs mapping to the “hot spots” which do not overlap with SLE and/or are previously identified as CLE susceptibility loci potentially represent specific association to the cutaneous disease.

### Therapeutic Considerations—Current and Future

Currently, no consensus or evidence based therapeutic regimen exists for CCLE. Moreover, very few well-controlled trials have systematically evaluated commonly used treatments for CLE (114–118). Disease management in CCLE begins with precise diagnosis and a treatment plan that can only be decided upon after comprehensive evaluation and recording of patient clinical and laboratory data. This includes immunological, clinical presentation, race, sex, age of onset, disease flares history, family history, past therapy failures, and histopathology of a skin biopsy among others.

Topical and systemic glucocorticoids, antimalarials, methotrexate and thalidomide are the standard of care in SLE, and are used in cutaneous disease as well. All treatments must be accompanied by intermittent re-evaluation of the patients to screen disease prognosis. The overall choices in therapy are discussed very briefly.

Tacrolimus (calcineurin inhibitor), may be a good substitute for those patients who respond poorly to topical corticosteroids (119). Antimalarial drugs (oral) such as chloroquine and quinacrine are also considered the first line of systemic CLE therapy (120–122). In refractory CLE that does not respond well to antimalarial therapy, the addition of medications that generally suppress the immune system is required. These include: methotrexate (123), mycophenolate mofetil (124), oral retinoids such as acitretin (125, 126), dapsone (127), as well as intravenous immunoglobulin (IVIG) (128, 129), Thalidomide and its structural analogs have also been used to treat recalcitrant CLE, with success in some cases (130). Trials assessing monoclonal antibodies such as sifalimumab and belimumab in CLE are on-going but mostly found to be effective in SLE with mucocutaneous involvement (131, 132).

We have mapped many of the drugs that are currently in clinical use for CCLE to their targets of influence in the “disease road map” based on mechanism of action (please refer to Figure 3). GO enrichment analyses of our transcriptional profiles reveal enhanced and activated leukocyte chemotaxis and B cell activation in skin more so than blood. This might explain the observed efficacy of the preventive topical corticosteroids (CS) treatments (114), since they bind to specific cytoplasmic receptors resulting in the inhibition of leukocyte infiltration at the site of inflammation. CS are equally effective in both humoral- and T cell mediated diseases (133). Over-represented disease-related processes such as lysosome/proteasome degradation in blood, as well as NK cells, and antigen processing and presentation in both skin and blood are expected to be the target of antimalarials (hydroxychloroquine or chloroquine), that are known to alter pH in lysosomes (134), modify TLR activation (135) and inhibit antigen presentation. We observe activated T cell related processes in CLE skin and blood analyses which are the predicted targets for several immunosuppressant medications such as methotrexate, a T cell proliferation inhibitor used in the treatment of recalcitrant CLE (123). Thalidomide and analog lenalidomide effectively target TNF-α in UV-induced apoptosis, thus decreasing inflammation. Prominent apoptotic signatures in both skin and blood presumably indicate effective use of these drugs, but they are not the drug of choice in cutaneous lupus due to potential serious side effects. Experimental therapeutic agents include azathropine, (anti-purine metabolite); apremilast, (phosphodiesterase-4 inhibitor); R333 (topical JAK/spleen tyrosine kinase inhibitor acting as an anti-inflammatory molecule), and etanercept (TNF-α inhibitor). Many of these agents are linked to clinical trials (Supplementary Table 7).

Clearly, healthcare is moving toward tailoring medicines/therapy targeting specific molecules in individual patients whose expression profiles are known. Our genome-wide transcriptome study is a representation of gene regulation in a highly balanced system of networks and pathways underlying CCLE. Disruption of any of the pathways by primary or secondary drug targeting offers unique treatment opportunities (136). Overall, our new *in silico* analyses identified a novel target, *CCR2* which is a key link to prominent disease related pathways and processes that merits further investigation for potential use in future therapy of CLE.

*Chemokine (C-C motif) receptor*2 (*CCR2*) encodes two isoforms of a receptor for monocyte chemoattractant protein-1 (MCP-1), which specifically mediates monocyte chemotaxis in inflammatory diseases such as rheumatoid arthritis. *CCR2* maps to the overlapping CCLE skin (FC = 1.8) and blood (FC = 1.6) “hot spot” on chromosome 3. Additionally, previous reports have found a positive correlation with increased gene expression in SLE (137, 138). This makes *CCR2* (implicated in the damaging inflammation underlying autoimmune and inflammatory diseases) a good potential therapeutic target candidate in cutaneous lupus as well. We discovered three xenobiotics: CCX915, CCX140 (clinical trials- phase I and II), and TAK779 (preclinical) that have been used to target the extracellular region of *CCR2*. CCX915 is a highly selective inhibitor of the *CCR2* chemokine receptor in multiple sclerosis and other autoimmune and inflammatory diseases. Preclinical data show that CCX140 selectively inhibits CCR2-mediated migration of monocytes and does not inhibit migration mediated by other chemokine receptors, even when the compound is given at high doses. This high degree of target specificity is an important safety feature that may allow CCX140 to be effective while avoiding unwanted side effects. We also discovered one highly specific humanized monoclonal antibody MLN1202 (clinical trial-Phase II) that interrupts MCP-1 binding to *CCR2*. These drugs (CCX915, CCX140-B, TAK779, and MLN 1202) have been used in the past in treatment of rheumatoid arthritis, diabetes mellitus, multiple sclerosis (139) and cardiovascular diseases with a modicum of success (140, 141) (Supplementary Table 7).

## Summary

Overall, the present study represents the first comparative analyses of CCLE skin and blood transcriptional profile along with “interactome-” “network-” “drug target-” analyses. The data is integrated and synthesized within the milieu of current literature on SLE/CCLE. We hypothesize a “disease road-map” demonstrating a coordinated orchestration of the autoimmune response in CCLE reflected in three phases: (1) initiation, (2) amplification, and (3) target damage in skin. *In -silico* interactome analyses was conducted to identify potential key functional players associated with the skin disease. Our careful and systematic downstream analyses of the CCLE skin and blood transcriptional data not only allowed us to uncover potential crucial contributors to the metabolic changes linked to the skin disease, but to select the best potential target candidate for future therapy.

*Chemokine (C-C motif) receptor*2 (*CCR2)* is the only molecule within the scope of our bioinformatics-guided analyses that fits all criteria we used to prioritize it as a potential drug target: (a) included as CCLE-DEG (blood and skin) by *microarray analysis*. The observed upregulation in case vs. control (blood) and lesional vs. non-lesional (skin) were accompanied by low e-values of ≤ 0.05, (b) overexpressed in disease vs. control peripheral blood by RT*-qPCR analysis, in a separate set of samples as those used in the microarray analysis*, (c) enriched in lupus-related pathways and processes by *ontology enrichment analyses*, (d) “over-connected” functional molecule by *interactome analysis*, (e) a reaction hub by *network analysis*, (f) mapped to chromosome 3 in an overlapping skin/blood transcriptional “hot spot” by *chromosome mapping analysis*, and (g) targeted by drugs that are currently being used to treat other diseases such as inflammatory and immune diseases by *drug target analysis*.

In summary, the present study, based on genome-wide gene expression aims to integrate clinical, genetic and bioinformatics data to bridge the gap between the laboratory and clinical management of patients. Information and evidence garnered from this report potentially impacts future clinical decision-making through the definition of actionable diagnostic and prognostic markers of the disease and the illumination of disease related pathways relevant to therapeutic response. Finally, we present the exciting possibility of designing potential new clinical trials (with a shorter cycle time due to pre-resolved regulatory issues) in the treatment of CCLE using *repurposed drugs* that target the CCLE related receptor, CCR2.

## Data Availability

The raw data associated with this study can be accessed associated with our two previous “Data in Brief” publications which are from skin (doi: 10.1016/j.dib.2015.02.024; PMID: 26217761) and blood (doi: 10.1016/j.dib.2014.11.006; PMID: 26217703).

## Ethics Statement

We have followed ethical conduct of research according to the Weill Cornell Medical College Institutional Review Board: WCM IRB# 0998-398. Signed consent forms were obtained from all patient and healthy control individuals before obtaining punch biopsies or performing blood draws.

## Author Contributions

RD-R contributed to experimentation, data analyses, research, conceptualizing, and writing of the manuscript and editing. AS contributed to design and procurement of samples for microarray and editing of manuscript.

### Conflict of Interest Statement

The authors declare that the research was conducted in the absence of any commercial or financial relationships that could be construed as a potential conflict of interest.

## Abbreviations

C/C/LE: chronic/cutaneous/lupus erythematosus
SLE: systemic lupus erythematosus
DLE: Discoid lupus erythematosus
HLA: human leukocyte antigen
DEGs: differentially expressed genes
DAVID: Database for Annotation, Visualization and Integrated Discovery
MHC: major histocompatibility complex
DC: dendritic cells
IFN: interferon
CTL: cytotoxic T-lymphocytes
IC: immune complex
GO: gene ontology
FC: fold change
NF-kB: nuclear factor kappa-light-chain-enhancer of activated B cells
qPCR/RT-qPCR: quantitative polymerase chain reaction/ real-time quantitative polymerase chain reaction.
