# Supplementary material for: In silico Analyses of Skin and Peripheral Blood Transcriptional Data in Cutaneous Lupus Reveals CCR2-A Novel Potential Therapeutic Target
Source: Front Immunol. 2019 Mar 29;10:640. doi: 10.3389/fimmu.2019.00640 (PMC6450170; doi:10.3389/fimmu.2019.00640)
Supplement: Supplementary file 1 [file Data_Sheet_1.PDF]

CCLE peripheral blood: 783 DEGs																		
							Disease related pathways and processes											
Probeset ID	Entrez Gene	Gene Symbol	Chromosomal Location	Gene Title	p-value	FC (CCLE patients vs. Control)	Apop	Oxid: Red	Cell adh	Cyto-chemo	Leu chem	NK	DC	TLR	TC	BC	IFN	Lys/Prot
39463_at	22848	*AAK1*	chr2p14	AP2 associated kinase 1	0.027	-1.2											√	
35390_at	23460	*ABCA6*	chr17q24.3	ATP-binding cassette, sub-family A (ABC1), member 6	0.033	-1.2												
41681_at	22	*ABCB7*	chrXq13.3	ATP-binding cassette, sub-family B (MDR/TAP), member 7	0.014	1.2												
40294_at	23457	*ABCB9*	chr12q24	ATP-binding cassette, sub-family B (MDR/TAP), member 9	0.015	-1.2		√										√
1930_at	8714	*ABCC3*	chr17q22	ATP-binding cassette, sub-family C (CFTR/MRP), member 3	0.001	-1.4												
37261_g_at	10060	*ABCC9*	chr12p12.1	ATP-binding cassette, sub-family C (CFTR/MRP), member 9	0.014	-1.2												
1635_at	25	*ABL1*	chr9q34.1	c-abl oncogene 1, non-receptor tyrosine kinase	0.047	1.2	√		√								√	
39058_at	29	*ABR*	chr17p13.3	active BCR-related gene	0.030	1.3												
36417_s_at	30	***ACAA1***	chr3p23-p22	acetyl-CoA acyltransferase 1	0.030	1.4		√										
39207_r_at	176	*ACAN*	chr15q26.1	aggrecan	0.046	-1.4			√									
37246_at	65057	*ACD*	chr16q22.1	adrenocortical dysplasia homolog (mouse)	0.033	-1.1												
41058_g_at	55856	*ACOT13*	chr6p22.3	acyl-CoA thioesterase 13	0.012	1.5												
34160_at	71	*ACTG1*	chr17q25	actin, gamma 1	0.028	1.2												
41654_at	100	*ADA*	chr20q13.12	adenosine deaminase	0.008	1.5			√		√				√	√		
37091_g_at	1587	*ADAM3A*	chr8p11.23	ADAM metallopeptidase domain 3A	0.012	-1.2												
36269_at	9508	*ADAMTS3*	chr4q13.3	ADAM metallopeptidase with thrombospondin type 1 motif, 3	0.041	-1.2												
33353_at	107	*ADCY1*	chr7p13-p12	adenylate cyclase 1 (brain)	0.042	-1.2				√							√	
34686_at	108	*ADCY2*	chr5p15.3	adenylate cyclase 2 (brain)	0.036	-1.2				√							√	
39383_at	112	*ADCY6*	chr12q12-q13	adenylate cyclase 6	0.038	-1.2				√							√	
34637_f_at	124	*ADH1A*	chr4q23	alcohol dehydrogenase 1A (class I), alpha polypeptide	0.029	-1.1		√										
31550_at	153	*ADRB1*	chr10q24-q26	adrenergic, beta-1-, receptor	0.029	-1.5												
34315_at	10939	*AFG3L2*	chr18p11	AFG3 ATPase family gene 3-like 2 (S. cerevisiae)	0.043	1.4												
38852_at	116986	*AGAP2*	chr12q14.1	ArfGAP with GTPase domain, ankyrin repeat and PH domain 2	0.042	-1.3												
31664_at	3268	*AGFG2*	chr7q22.1	ArfGAP with FG repeats 2	0.016	-1.3												
37880_at	189	*AGXT*	chr2q37.3	alanine-glyoxylate aminotransferase	0.030	-1.3												
34728_g_at	10768	***AHCYL1***	chr1p13.2	adenosylhomocysteinase-like 1	0.050	1.6												
36497_at	113146	***AHNAK2***	chr14q32.33	AHNAK nucleoprotein 2	0.004	-1.2												
34439_at	9447	***AIM2***	chr1q22	absent in melanoma 2	0.029	-1.1	√										√	
41250_at	7965	*AIMP2*	chr7p22	aminoacyl tRNA synthetase complex-interacting multifunctional protein 2	0.006	1.4												
40685_at	221	*ALDH3B1*	chr11q13	aldehyde dehydrogenase 3 family, member B1	0.012	1.6												
678_at	251	*ALPPL2*	chr2q37	alkaline phosphatase, placental-like 2	0.050	-1.4												
33532_at	8092	*ALX1*	chr12q21.31	ALX homeobox 1	0.014	-1.2			√									
41706_at	114899 /// 23600	*AMACR /// C1QTNF3*	chr5p13	alpha-methylacyl-CoA racemase /// C1q and tumor necrosis factor related protein 3	0.000	-1.2												
35502_at	269	*AMHR2*	chr12q13	anti-Mullerian hormone receptor, type II	0.032	-1.2				√								
32919_at	347902	*AMIGO2*	chr12q13.11	adhesion molecule with Ig-like domain 2	0.012	-1.1			√									
36865_at	23357	*ANGEL1*	chr14q24.3	angel homolog 1 (Drosophila)	0.010	-1.2												
38199_at	90806	*ANGEL2*	chr1q32.3	angel homolog 2 (Drosophila)	0.028	-1.2												
40347_at	81611	***ANP32E***	chr1q21.2	acidic (leucine-rich) nuclear phosphoprotein 32 family, member E	0.023	1.5												
37747_at	308	*ANXA5*	chr4q26-q28|4q27	annexin A5	0.020	1.9												
35593_at	314	*AOC2*	chr17q21	amine oxidase, copper containing 2 (retina-specific)	0.019	-1.3												
36161_at	163	*AP2B1*	chr17q11.2-q12	adaptor-related protein complex 2, beta 1 subunit	0.039	1.4												
32030_at	10239	***AP3S2***	chr15q26.1	adaptor-related protein complex 3, sigma 2 subunit	0.035	1.2												√
40148_at	323	*APBB2*	chr4p13	amyloid beta (A4) precursor protein-binding, family B, member 2	0.025	-1.1												
39106_at	335	***APOA1***	chr11q23-q24	apolipoprotein A-I	0.047	-1.3							√					
261_s_at	338	*APOB*	chr2p24-p23	apolipoprotein B (including Ag(x) antigen)	0.018	-1.2												
41472_at	60489	***APOBEC3G***	chr22q13.1-q13.2	apolipoprotein B mRNA editing enzyme, catalytic polypeptide-like 3G	0.018	2.4												
40430_at	375	*ARF1*	chr1q42.13	ADP-ribosylation factor 1	0.048	-1.3												
34725_at	2909	*ARHGAP35*	chr19q13.3	glucocorticoid receptor DNA binding factor 1	0.045	-1.2												
35505_at	8289	*ARID1A*	chr1p35.3	AT rich interactive domain 1A (SWI-like)	0.048	-1.4											√	
31375_at	403	*ARL3*	chr10q23.3	ADP-ribosylation factor-like 3	0.049	-1.2												
39829_at	10123	***ARL4C***	chr2q37.1	ADP-ribosylation factor-like 4C	0.030	1.7												
32701_at	421	*ARVCF*	chr22q11.21	armadillo repeat gene deleted in velocardiofacial syndrome	0.013	-1.1			√									
31875_at	51665	*ASB1*	chr2q37	ankyrin repeat and SOCS box-containing 1	0.018	1.1	√											
287_at	467	*ATF3*	chr1q32.3	activating transcription factor 3	0.002	1.2	√										√	
41235_at	468	*ATF4*	chr22q13.1	activating transcription factor 4 (tax-responsive enhancer element B67)	0.039	1.6	√										√	
32439_at	496	*ATP4B*	chr13q34	ATPase, H+/K+ exchanging, beta polypeptide	0.033	-1.3												
34811_at	518	*ATP5G3*	chr2q31.1	ATP synthase, H+ transporting, mitochondrial Fo complex, subunit C3 (subunit 9)	0.046	1.5												
36107_at	522	*ATP5J*	chr21q21.1	ATP synthase, H+ transporting, mitochondrial Fo complex, subunit F6	0.024	1.5												
40134_at	9551	*ATP5J2*	chr7q22.1	ATP synthase, H+ transporting, mitochondrial Fo complex, subunit F2	0.029	1.5												
33854_at	51382	*ATP6V1D*	chr14q23-q24.2	ATPase, H+ transporting, lysosomal 34kDa, V1 subunit D	0.042	1.4												
37367_at	529	*ATP6V1E1*	chr22pter-q11.2|22q11.1	ATPase, H+ transporting, lysosomal 31kDa, V1 subunit E1	0.034	1.2												
37395_at	9296	*ATP6V1F*	chr7q32	ATPase, H+ transporting, lysosomal 14kDa, V1 subunit F	0.044	1.3		√										
33741_at	51606	*ATP6V1H*	chr8q11.2	ATPase, H+ transporting, lysosomal 50/57kDa, V1 subunit H	0.012	1.2												√
33634_at	5205	*ATP8B1*	chr18q21-q22|18q21.31	ATPase, aminophospholipid transporter, class I, type 8B, member 1	0.031	-1.2												
36142_at	6310	*ATXN1*	chr6p23	ataxin 1	0.041	1.6												
34487_at	6310	*ATXN3*	chr6p23	ataxin 3	0.017	-1.2												
39401_at	552889	*ATXN7L3B*	chr12q21	ataxin 7-like 3B	0.010	1.2												
41582_at	10677	*AVIL*	chr12q14.1	advillin	0.012	-1.2												
1278_at	558	*AXL*	chr19q13.1	AXL receptor tyrosine kinase	0.017	1.4												
39432_at	8702	*B4GALT4*	chr3q13.3	UDP-Gal:betaGlcNAc beta 1,4- galactosyltransferase, polypeptide 4	0.004	1.5												
41115_s_at	8938	*BAIAP3*	chr16p13.3	BAI1-associated protein 3	0.026	-1.3												
1801_at	580	*BARD1*	chr2q34-q35	BRCA1 associated RING domain 1	0.034	2												
35425_at	8538	*BARX2*	chr11q25	BARX homeobox 2	0.007	-1.2			√									
1909_at	596	*BCL2*	chr18q21.33|18q21.3	B-cell CLL/lymphoma 2	0.044	1.3	√		√		√				√	√		
31611_s_at	10018	*BCL2L11*	chr2q13	BCL2-like 11 (apoptosis facilitator)	0.002	1.2	√		√							√		
1339_s_at	613	*BCR*	chr22q11|22q11.23	breakpoint cluster region	0.029	-1.1												
32127_at	636	***BICD1***	chr12p11.2-p11.1	bicaudal D homolog 1 (Drosophila)	0.023	1.1												
35266_at	10904	*BLCAP*	chr20q11.23	bladder cancer associated protein	0.012	1.3												
1544_at	641	*BLM*	chr15q26.1	Bloom syndrome, RecQ helicase-like	0.019	1.4					√				√			

37700_at	642	*BLMH*	chr17q11.2	bleomycin hydrolase	0.003	1.4												
32618_at	644	*BLVRA*	chr7p14-cen	biliverdin reductase A	0.025	2.1												
32088_at	8548	*BLZF1*	chr1q24	basic leucine zipper nuclear factor 1	0.026	1.2												
1831_at	653	*BMP5*	chr6p12.1	bone morphogenetic protein 5	0.042	-1.3												
35021_at	658	*BMPR1B*	chr4q22-q24	bone morphogenetic protein receptor, type IB	0.033	-1.2	√		√	√								
38709_at	9790	*BMS1*	chr10q11.21	BMS1 homolog, ribosome assembly protein (yeast)	0.047	1.3												
41732_at	552900	*BOLA2*	chr16p11.2	bolA homolog 2 (E. coli)	0.032	1.3		√										
41512_at	8315	*BRAP*	chr12q24	BRCA1 associated protein	0.041	1.1												
141_s_at	2972	*BRF1*	chr14q	BRF1 homolog, subunit of RNA polymerase III	0.035	-1.2	√											
35154_at	55643	*BTBD2*	chr19p13.3	BTB (POZ) domain containing 2	0.033	-1.3												
33975_at	685	*BTC*	chr4q13-q21	betacellulin	0.014	-1.2												
41802_at	79703	*C11orf80*	chr11q13.2	Chromosome 11 open reading frame 80	0.046	-1.1												
38967_at	9556	*C14orf2*	chr14q32.33	chromosome 14 open reading frame 2	0.021	1.6												
37884_f_at	29798	*C2orf27A*	chr2q21.2	chromosome 2 open reading frame 27A	0.007	-1.1												
31971_at	23434	*C3orf27*	chr3q21	chromosome 3 open reading frame 27	0.010	-1.4												
38367_s_at	725	*C4BPB*	chr1q32	complement component 4 binding protein, beta	0.033	-1.2										√		
39241_at	759	*CA1*	chr8q21.2	carbonic anhydrase I	0.047	-1.1												
33623_g_at	775	*CACNA1C*	chr12p13.3	calcium channel, voltage-dependent, L type, alpha 1C subunit	0.019	-1.5												
31993_f_at	783	*CACNB2*	chr10p12	calcium channel, voltage-dependent, beta 2 subunit	0.011	-1.4												
34116_at	785	*CACNB4*	chr2q22-q23	calcium channel, voltage-dependent, beta 4 subunit	0.041	-1.2									√			
37157_at	794	*CALB2*	chr16q22.2	calbindin 2	0.003	-1.3												
38721_at	57658	***CALCOCO1***	chr12q13.13	calcium binding and coiled-coil domain 1	0.010	-1.2												
1158_s_at	808	*CALM3*	chr19q13.2-q13.3	calmodulin 3 (phosphorylase kinase, delta)	0.023	-1.2											√	
32863_at	57172	*CAMK1G*	chr1q32-q41	calcium/calmodulin-dependent protein kinase IG	0.038	-1.2												
33404_at	10486	***CAP2***	chr6p22.3	CAP, adenylate cyclase-associated protein, 2 (yeast)	0.013	-1.3												
38391_at	822	*CAPG*	chr2p11.2	capping protein (actin filament), gelsolin-like	0.021	1.5												
37001_at	824	*CAPN2*	chr1q41-q42	calpain 2, (m/II) large subunit	0.027	2	√											
40475_at	827	*CAPN6*	chrXq23	calpain 6	0.033	-1.2												
36138_at	826	*CAPNS1*	chr19q13.12	calpain, small subunit 1	0.036	1.2												
39999_at	843	***CASP10***	chr2q33-q34	caspase 10, apoptosis-related cysteine peptidase	0.013	1.6	√					√						
38958_at	846	*CASR*	chr3q13	calcium-sensing receptor	0.027	-1.2												
41072_at	859	*CAV3*	chr3p25	caveolin 3	0.023	-1.2												
36691_at	883	*CCBL1*	chr9q34.11	cysteine conjugate-beta lyase, cytoplasmic	0.013	-1.2												
35106_at	881	*CCIN*	chr9p13.3	calicin	0.028	-1.2												
2021_s_at	898	*CCNE1*	chr19q12	cyclin E1	0.014	-1.2												
39936_at	729230	***CCR2***	chr3p21.31	chemokine (C-C motif) receptor 2	0.006	1.6				√	√		√				√	
40774_at	7203	*CCT3*	chr1q23	chaperonin containing TCP1, subunit 3 (gamma)	0.010	1.4												
38720_at	10574	*CCT7*	chr2p13.2	chaperonin containing TCP1, subunit 7 (eta)	0.012	1.3												
36661_s_at	929	*CD14*	chr5q22-q32|5q31.1	CD14 molecule	0.018	1.9								√			√	
31438_s_at	9332	***CD163***	chr12p13.3	CD163 molecule	0.000	2												
38567_at	912	***CD1D***	chr1q22-q23	CD1d molecule	0.002	1.6					√				√			
39226_at	917	***CD3G***	chr11q23	CD3g molecule, gamma (CD3-TCR complex)	0.050	1.5	√				√	√			√		√	
38006_at	962	***CD48***	chr1q21.3-q22	CD48 molecule	0.045	1.5					√	√			√			
37003_at	967	*CD63*	chr12q12-q13	CD63 molecule	0.049	1.5												√
771_s_at	924	*CD7*	chr17q25.2-q25.3	CD7 molecule	0.039	-1.5					√				√			
35282_r_at	975	*CD81*	chr11p15.5	CD81 molecule	0.029	1.3									√	√		
40920_at	8555	*CDC14B*	chr9q22.3	CDC14 cell division cycle 14 homolog B (S. cerevisiae)	0.005	-1.3												
37458_at	8318	*CDC45*	chr22q11.21	cell division cycle 45 homolog (S. cerevisiae)	0.046	-1.3												
32266_at	1010	*CDH12*	chr5p14.3	cadherin 12, type 2 (N-cadherin 2)	0.035	-1.2			√									
37857_at	92211	*CDHR1*	chr10q23.1	cadherin-related family member 1	0.005	-1.2			√									
41820_s_at	8621	*CDK13*	chr7p13	Cyclin-dependent kinase 13	0.043	-1.2												
2031_s_at	1026	*CDKN1A*	chr6p21.2	cyclin-dependent kinase inhibitor 1A (p21, Cip1)	0.047	1.3	√				√					√		
40392_at	1045	*CDX2*	chr13q12.3	caudal type homeobox 2	0.046	-1.3											√	
39219_at	1054	*CEBPG*	chr19q13.11	CCAAT/enhancer binding protein (C/EBP), gamma	0.024	1.9					√						√	
38936_at	63036	*CELA2A*	chr1p36.21	chymotrypsin-like elastase family, member 2A	0.010	-1.3												
31478_at	51032	*CELA2B*	chr1p36.21	chymotrypsin-like elastase family, member 2B	0.035	-1.2												
41660_at	9620	*CELSR1*	chr22q13.3	cadherin, EGF LAG seven-pass G-type receptor 1	0.009	-1.2			√									
35496_at	1068	*CETN1*	chr18p11.32	centrin, EF-hand protein, 1	0.049	-1.3												
33469_r_at	10878	*CFHR3*	chr1q32	complement factor H-related 3	0.031	-1.2												
38920_at	1111	*CHEK1*	chr11q24.2	CHK1 checkpoint homolog (S. pombe)	0.030	-1.3	√											
40808_at	1113	*CHGA*	chr14q32	chromogranin A (parathyroid secretory protein 1)	0.035	-1.4												
32425_at	1135	*CHRNA2*	chr8p21	cholinergic receptor, nicotinic, alpha 2 (neuronal)	0.021	-1.5												
33403_at	26097	*CHTOP*	chr1q21.3	chromatin target of PRMT1	0.033	1.5												
40690_at	1164	*CKS2*	chr9q22	CDC28 protein kinase regulatory subunit 2	0.001	1.5												
36809_at	1178	*CLC*	chr19q13.1	Charcot-Leyden crystal protein	0.010	-2.8												
36771_at	1269	*CNR2*	chr1p36.11	cannabinoid receptor 2 (macrophage)	0.024	-1.2												
38952_s_at	1305	*COL13A1*	chr10q22	collagen, type XIII, alpha 1	0.018	-1.4			√									
39550_at	23127	*COLGALT2*	chr1q25	collagen beta(1-O)galactosyltransferase 2	0.044	-1.3												
1789_at	10987	*COPS5*	chr8q13.1	COP9 constitutive photomorphogenic homolog subunit 5	0.050	1.5											√	
34772_at	10391	*CORO2B*	chr15q23	coronin, actin binding protein, 2B	0.023	-1.1												
41760_at	1347	*COX7A2*	chr6q12	cytochrome c oxidase subunit VIIa polypeptide 2 (liver)	0.045	1.4												
36687_at	1349	*COX7B*	chrXq21.1	cytochrome c oxidase subunit VIIb	0.016	1.6												
40748_at	1358	*CPA2*	chr7q32	carboxypeptidase A2 (pancreatic)	0.003	-1.2												
37172_at	1361	*CPB2*	chr13q14.11	carboxypeptidase B2 (plasma)	0.001	-1.3												
40650_r_at	1394	*CRHR1*	chr17q12-q22	corticotropin releasing hormone receptor 1	0.009	-1.3											√	
38925_at	7180	*CRISP2*	chr6p12.3	cysteine-rich secretory protein 2	0.047	1.2												
772_at	1398	*CRK*	chr17p13.3	v-crk sarcoma virus CT10 oncogene homolog (avian)	0.011	-1.2				√								
36389_at	56253	***CRTAM***	chr11q24.1	cytotoxic and regulatory T cell molecule	0.049	1.3	√				√				√			
40119_at	10491	*CRTAP*	chr3p22.3	cartilage associated protein	0.023	-1.2												
1170_at	1435	*CSF1*	chr1p21-p13	colony stimulating factor 1 (macrophage)	0.042	-1.3			√	√	√							
410_s_at	1460	*CSNK2B*	chr6p21-p12|6p21.3	casein kinase 2, beta polypeptide	0.039	1.2	√											
34009_at	30848	*CTAG2*	chrXq28	cancer/testis antigen 2	0.024	-1.3												
2069_s_at	1495	*CTNNA1*	chr5q31	catenin (cadherin-associated protein), alpha 1, 102kDa	0.032	1.6			√									
32372_at	1508	*CTSB*	chr8p22	cathepsin B	0.037	1.8	√											√
133_at	1075	*CTSC*	chr11q14.2	cathepsin C	0.034	1.7												√

39846_at	8722	*CTSF*	chr11q13	cathepsin F	0.040	-1.4												√
37021_at	1512	*CTSH*	chr15q25.1	cathepsin H	0.040	1.8	√											√
37391_at	1514	***CTSL***	chr9q21.33	cathepsin L	0.002	2.6	√											√
32515_s_at	1522	*CTSZ*	chr20q13	Cathepsin Z	0.042	-1.3												√
39724_s_at	8454	*CUL1*	chr7q36.1	cullin 1	0.005	2.3	√										√	
40646_at	1524	*CX3CR1*	chr3p21|3p21.3	chemokine (C-X3-C motif) receptor 1	0.031	2.3			√	√								
1033_g_at	3579	*CXCR2*	chr2q35	chemokine (C-X-C motif) receptor 2	0.034	-2				√	√						√	
38459_g_at	1528	***CYB5A***	chr18q23	cytochrome b5 type A (microsomal)	0.019	1.2		√										
37306_at	23191	*CYFIP1*	chr15q11	cytoplasmic FMR1 interacting protein 1	0.027	1.8												
34548_at	1584	*CYP11B1*	chr8q21	cytochrome P450, family 11, subfamily B, polypeptide 1	0.007	-1.3												
1080_s_at	1544	*CYP1A2*	chr15q24.1	cytochrome P450, family 1, subfamily A, polypeptide 2	0.015	-1.2		√										
34983_at	1592	*CYP26A1*	chr10q23-q24	Cytochrome P450, family 26, subfamily A, polypeptide 1	0.047	-1.4												
40379_at	1571	*CYP2E1*	chr10q24.3-qter	cytochrome P450, family 2, subfamily E, polypeptide 1	0.043	-1.3												
37862_at	1629	*DBT*	chr1p31	dihydrolipoamide branched chain transacylase E2	0.004	-1.2												
630_at	1635	*DCTD*	chr4q35.1	dCMP deaminase	0.009	1.7												
36220_at	23576	*DDAH1*	chr1p22	dimethylarginine dimethylaminohydrolase 1	0.027	-1.2												
1243_at	1643	*DDB2*	chr11p12-p11	damage-specific DNA binding protein 2, 48kDa	0.004	1.7											√	
41180_i_at	1652	*DDT*	chr22q11.23	D-dopachrome tautomerase	0.012	-1.2												
149_at	10212	*DDX39*	chr19p13.12	DEAD (Asp-Glu-Ala-Asp) box polypeptide 39	0.033	1.6												
38191_at	9681	*DEPDC5*	chr22q12.3	DEP domain containing 5	0.023	-1.2		√										
37335_at	1716	*DGUOK*	chr2p13	deoxyguanosine kinase	0.008	1.2												
39896_at	8449	*DHX16*	chr6p21.3	DEAH (Asp-Glu-Ala-His) box polypeptide 16	0.033	1.3												
31857_r_at	9704	*DHX34*	chr19q13.3	DEAH (Asp-Glu-Ala-His) box polypeptide 34	0.012	-1.5												
34829_at	1736	*DKC1*	chrXq28	dyskeratosis congenita 1, dyskerin	0.012	1.2												
33594_at	27121	*DKK4*	chr8p11.2-p11.1	dickkopf homolog 4 (Xenopus laevis)	0.001	-1.3												
32648_at	8788	*DLK1*	chr14q32	delta-like 1 homolog (Drosophila)	0.031	-1.2												
33257_at	1743	*DLST*	chr14q24.3	dihydrolipoamide S-succinyltransferase (E2 component)	0.037	-1.3												
276_at	3301	*DNAJA1*	chr9p13.3	DnaJ (Hsp40) homolog, subfamily A, member 1	0.023	1.4	√											
33533_at	11080	*DNAJB4*	chr1p31.1	DnaJ (Hsp40) homolog, subfamily B, member 4	0.028	-1.2												
40830_at	3338	*DNAJC4*	chr11q13	DnaJ (Hsp40) homolog, subfamily C, member 4	0.041	-1.3												
34463_at	1773	*DNASE1*	chr16p13.3	deoxyribonuclease I	0.032	-1.3												
37214_g_at	1774	*DNASE1L1*	chrXq28	deoxyribonuclease I-like 1	0.041	1.5												
37413_at	1800	*DPEP1*	chr16q24.3	dipeptidase 1 (renal)	0.015	-1.3												
35679_s_at	1804	*DPP6*	chr7q36.2	dipeptidyl-peptidase 6	0.006	-1.3												
33050_at	1816	*DRD5*	chr4p16.1	dopamine receptor D5	0.038	-1.2												
34619_at	1823	*DSC1*	chr18q12.2|18q12.1	desmocollin 1	0.013	-1.3			√									
36699_at	1826	*DSCAM*	chr21q22.2	Down syndrome cell adhesion molecule	0.003	-1.2			√									
1292_at	1844	*DUSP2*	chr2q11	dual specificity phosphatase 2	0.009	1.6												
529_at	1847	*DUSP5*	chr10q25	dual specificity phosphatase 5	0.036	1.7												
1561_at	1850	*DUSP8*	chr11p15.5	dual specificity phosphatase 8	0.007	-1.2												
1443_at	1852	*DUSP9*	chrXq28	dual specificity phosphatase 9	0.030	-1.4												
37300_at	1778	*DYNC1H1*	chr14q32.3-qter|14q32	dynein, cytoplasmic 1, heavy chain 1	0.039	1.5												
946_at	6993	***DYNLT1***	chr6q25.2-q25.3	dynein, light chain, Tctex-type 1	0.031	1.7												
36521_at	22873	*DZIP1*	chr13q32.1	DAZ interacting protein 1	0.017	-1.1												
32756_at	1891	***ECH1***	chr19q13.1	enoyl CoA hydratase 1, peroxisomal	0.022	1.5												
1507_s_at	1909	*EDNRA*	chr4q31.22	endothelin receptor type A	0.010	-1.2											√	
40425_at	1942	***EFNA1***	chr1q21-q22	ephrin-A1	0.022	-1.2												
38097_at	9538	*EI24*	chr11q24	etoposide induced 2.4 mRNA	0.027	1.3												
38662_at	5610	*EIF2AK2*	chr2p22-p21	eukaryotic translation initiation factor 2-alpha kinase 2	0.038	2.7	√										√	
40616_at	8669	*EIF3J*	chr15q21.1	eukaryotic translation initiation factor 3, subunit J	0.043	1.6												
1199_at	1973	*EIF4A1*	chr17p13	eukaryotic translation initiation factor 4A1	0.005	1.4												
38031_at	9775	*EIF4A3*	chr17q25.3	eukaryotic translation initiation factor 4A3	0.041	1.3												
167_at	1983	*EIF5*	chr14q32.32	eukaryotic translation initiation factor 5	0.022	1.4												
40536_f_at	9669	*EIF5B*	chr2q11.2	Eukaryotic translation initiation factor 5B	0.005	1.1												
36411_s_at	1993	*ELAVL2*	chr9p21	ELAV (embryonic lethal, abnormal vision, Drosophila)-like 2	0.028	-1.2												
40445_at	1999	*ELF3*	chr1q32.2	E74-like factor 3 (ets domain transcription factor, epithelial-specific)	0.033	-1.1												
31913_at	2001	*ELF5*	chr11p13-p12	E74-like factor 5 (ets domain transcription factor)	0.009	-1.1												
31621_s_at	2006	*ELN*	chr7q11.23	elastin	0.033	-1.3												
31755_at	2020	*EN2*	chr7q36	engrailed homeobox 2	0.018	-1.2												
2035_s_at	2023	*ENO1*	chr1p36.2	enolase 1, (alpha)	0.030	1.6												
39084_at	2027	*ENO3*	chr17pter-p11	enolase 3 (beta, muscle)	0.007	-1.2												
39010_at	2029	*ENSA*	chr1q21.3	endosulfine alpha	0.042	1.4												
1605_g_at	2044	*EPHA5*	chr4q13.1	EPH receptor A5	0.017	-1.2												
1901_s_at	2064	*ERBB2*	chr17q11.2-q12|17q21.1	v-erb-b2 erythroblastic leukemia viral oncogene homolog 2	0.036	-1.3	√		√		√				√			
33639_g_at	2065	***ERBB3***	chr12q13	v-erb-b2 erythroblastic leukemia viral oncogene homolog 3 (avian)	0.041	-1.2	√		√									
37869_at	23085	*ERC1*	chr12p13.3	ELKS/RAB6-interacting/CAST family member 1	0.023	-1.3												
39079_at	2079	*ERH*	chr14q24.1|7q34	enhancer of rudimentary homolog (Drosophila)	0.010	1.4												
36357_at	112479	*ERI2*	chr16p12.3	ERI1 exoribonuclease family member 2	0.005	-1.2												
31380_at	2081	*ERN1*	chr17q24.2	endoplasmic reticulum to nucleus signaling 1	0.042	-1.2	√											
31437_r_at	2100	*ESR2*	chr14q23.2	estrogen receptor 2 (ER beta)	0.009	-1.4	√										√	
39569_at	2125	*EVPL*	chr17q25	envoplakin	0.008	-1.2												
39625_at	23086	***EXPH5***	chr11q22.3	exophilin 5	0.049	-1.2												
36345_g_at	2150	*F2RL1*	chr5q13	coagulation factor II (thrombin) receptor-like 1	0.006	-1.3											√	
36543_at	2152	***F3***	chr1p22-p21	coagulation factor III (thromboplastin, tissue factor)	0.043	1.1												
38586_at	2168	*FABP1*	chr2p11	fatty acid binding protein 1, liver	0.035	-1.2												
33856_at	8933	*FAM127A*	chrXq26	family with sequence similarity 127, member A	0.037	1.4												
41873_at	9715	*FAM131B*	chr7q34	family with sequence similarity 131, member B	0.013	-1.2												
41687_r_at	202134	*FAM153B*	chr5q35.2	Family with sequence similarity 153, member B	0.000	-1.2												
39434_at	387680	*FAM21A*	chr10q11.23	family with sequence similarity 21, member A	0.022	1.6												
33433_at	25940	*FAM98A*	chr2p22.3	family with sequence similarity 98, member A	0.041	1.4												
160034_s_at	2176	*FANCC*	chr9q22.3	Fanconi anemia, complementation group C	0.005	-1.4												
34291_at	2193	*FARSA*	chr19p13.2	phenylalanyl-tRNA synthetase, alpha subunit	0.037	1.1		√										
38027_at	2192	*FBLN1*	chr22q13.31	fibulin 1	0.030	-1.2												
39038_at	10516	*FBLN5*	chr14q32.1	fibulin 5	0.030	-1.5			√									
32535_at	2200	*FBN1*	chr15q21.1	fibrillin 1	0.022	-1.2												

34848_at	2222	*FDFT1*	chr8p23.1-p22	farnesyl-diphosphate farnesyltransferase 1	0.034	1.2		√										
41583_at	2237	***FEN1***	chr11q12	flap structure-specific endonuclease 1	0.032	1.4												
31487_at	9637	***FEZ2***	chr2p21	fasciculation and elongation protein zeta 2 (zygin II)	0.006	-1.2												
38825_at	2243	*FGA*	chr4q28	fibrinogen alpha chain	0.029	-1.2												
41586_at	8817	*FGF18*	chr5q34	fibroblast growth factor 18	0.012	-1.4												
41806_at	2247	*FGF2*	chr4q26	fibroblast growth factor 2 (basic)	0.047	-1.3												
1732_at	2250	*FGF5*	chr4q21	fibroblast growth factor 5	0.047	-1.2												
1525_s_at	2253	*FGF8*	chr10q24	fibroblast growth factor 8 (androgen-induced)	0.027	-1.2												
35081_at	2254	*FGF9*	chr13q11-q12	fibroblast growth factor 9 (glia-activating factor)	0.014	-1.3												
1145_g_at	2263	***FGFR2***	chr10q26	fibroblast growth factor receptor 2	0.049	-1.3												
31897_at	11259	*FILIP1L*	chr3q12.1	filamin A interacting protein 1-like	0.040	-1.2												
31376_at	2319	*FLOT2*	chr17q11-q12	flotillin 2	0.023	-1.2			√									
31980_at	8456	*FOXN1*	chr17q11-q12	forkhead box N1	0.033	-1.3												
35030_i_at	9758	*FRMPD4*	chrXp22.2	FERM and PDZ domain containing 4	0.042	-1.2												
35083_at	2512	*FTL*	chr19q13.33	ferritin, light polypeptide	0.021	1.1												
38300_at	8321	*FZD1*	chr7q21	frizzled homolog 1 (Drosophila)	0.001	1.2												
628_at	2535	***FZD2***	chr17q21.1	frizzled homolog 2 (Drosophila)	0.041	1.1												
36323_at	2554	*GABRA1*	chr5q34-q35	gamma-aminobutyric acid (GABA) A receptor, alpha 1	0.019	-1.2												
33197_M_at	2597	*GAPDH*	chr12p13	glyceraldehyde-3-phosphate dehydrogenase	0.049	1.4												
34542_at	26330	*GAPDHS*	chr19q13.12	glyceraldehyde-3-phosphate dehydrogenase, spermatogenic	0.045	-1.2												
36581_at	2617	***GARS***	chr7p15	glycyl-tRNA synthetase	0.002	1.7												
661_at	2619	***GAS1***	chr9q21.3-q22	growth arrest-specific 1	0.031	-1.1	√											
34552_at	2520	*GAST*	chr17q21	gastrin	0.005	-1.2												
37194_at	2624	*GATA2*	chr3q21.3	GATA binding protein 2	0.050	-1.5											√	
32502_at	81544	*GDPD5*	chr11q13.4-q13.5	glycerophosphodiester phosphodiesterase domain containing 5	0.030	-1.3												
1692_s_at	2675	*GFRA2*	chr8p21.3	GDNF family receptor alpha 2	0.033	-1.2												
32002_at	2676	*GFRA3*	chr5q31.1-q31.3	GDNF family receptor alpha 3	0.045	-1.4												
308_f_at	2689	*GH2*	chr17q24.2	growth hormone 2	0.021	-1.3				√								
160030_at	2690	*GHR*	chr5p13-p12	growth hormone receptor	0.004	-1.3				√							√	
1390_s_at	2691	*GHRH*	chr20q11.2	growth hormone releasing hormone	0.031	-1.3												
32531_at	2697	*GJA1*	chr6q21-q23.2	gap junction protein, alpha 1, 43kDa	0.050	1.1												
2074_at	2702	*GJA5*	chr1q21.1	gap junction protein, alpha 5, 40kDa	0.038	-1.3												
38903_at	2709	*GJB5*	chr1p35.1	gap junction protein, beta 5, 31.1kDa	0.023	-1.4												
38129_at	2710	***GK***	chrXp21.3	glycerol kinase	0.021	-1.3												
34048_at	2712	*GK2*	chr4q13	glycerol kinase 2	0.002	-1.1												
36833_at	2717	*GLA*	chrXq22	galactosidase, alpha	0.034	1.2												√
37742_at	2720	*GLB1*	chr3p21.33	galactosidase, beta 1	0.021	1.6												√
31310_at	2741	*GLRA1*	chr5q32	glycine receptor, alpha 1	0.027	-1.2												
34554_at	2742	*GLRA2*	chrXp22.1-p21.3	glycine receptor, alpha 2	0.038	-1.2												
31357_at	2744	***GLS***	chr2q32-q34	glutaminase	0.034	-1.1												
35820_at	2760	*GM2A*	chr5q33.1	GM2 ganglioside activator	0.040	1.5												√
36772_at	2792	*GNGT1*	chr7q21.3	guanine nucleotide binding protein (G protein)	0.022	-1.2				√							√	
36930_at	29889	*GNL2*	chr1p34.3	guanine nucleotide binding protein-like 2 (nucleolar)	0.003	1.5												
37145_at	10578	***GNLY***	chr2p11.2	granulysin	0.010	-2.7							√					
34332_at	10007	*GNPDA1*	chr5q21	glucosamine-6-phosphate deaminase 1	0.039	1.4		√										
38620_at	9570	*GOSR2*	chr17q21	golgi SNAP receptor complex member 2	0.012	1.2												
33063_at	2811	*GP1BA*	chr17pter-p12	glycoprotein Ib (platelet), alpha polypeptide	0.008	-1.4			√									
35101_at	2815	*GP9*	chr3q21.3	glycoprotein IX (platelet)	0.009	-1.2			√									
41102_at	27238	*GPKOW*	chrXp11.23	G patch domain and KOW motifs	0.031	1.1												
41756_at	11321 /// 84450	*GPN1 /// ZNF512*	chr2p23 /// chr2p23.3	GPN-loop GTPase 1 /// zinc finger protein 512	0.017	1.2												
40301_at	23432	*GPR161*	chr1q24.2	G protein-coupled receptor 161	0.047	-1.3												
36429_at	11318	*GPR182*	chr12q13.3	G protein-coupled receptor 182	0.039	-1.4												
39573_at	2898	*GRIK2*	chr6q16.3-q21	glutamate receptor, ionotropic, kainate 2	0.047	-1.2												
41198_at	2896	*GRN*	chr17q21.32	granulin	0.019	1.9												
272_at	2922	*GRP*	chr18q21.1-q21.32	gastrin-releasing peptide	0.040	-1.4												
32430_at	2925	*GRPR*	chrXp22.2	gastrin-releasing peptide receptor	0.019	-1.4											√	
41631_f_at	2937	*GSS*	chr20q11.2	Glutathione synthetase	0.049	-1.2												
1121_g_at	2947	*GSTM3*	chr1p13.3	glutathione S-transferase mu 3 (brain)	0.036	-1.5												
829_s_at	2950	*GSTP1*	chr11q13	glutathione S-transferase pi 1	0.007	1.2												
36510_at	2963	*GTF2F2*	chr13q14	general transcription factor IIF, polypeptide 2, 30kDa	0.019	1.2												
39162_at	404672	*GTF2H5*	chr6q25.3	general transcription factor IIH, polypeptide 5	0.045	1.3												
35958_at	84163	*GTF2IRD2*	chr7q11.23	GTF2I repeat domain containing 2	0.045	-1.4												
41263_at	9567	*GTPBP1*	chr22q13.1	GTP binding protein 1	0.041	-1.1												
34450_at	2984	*GUCY2C*	chr12p12	guanylate cyclase 2C (heat stable enterotoxin receptor)	0.021	-1.1												
36576_at	9555	*H2AFY*	chr5q31.1	H2A histone family, member Y	0.014	1.6												
37166_at	23498	*HAAO*	chr2p21	3-hydroxyanthranilate 3,4-dioxygenase	0.030	-1.2		√										
33072_at	3062	*HCRTR2*	chr6p12	hypocretin (orexin) receptor 2	0.048	-1.3											√	
38771_at	3065	***HDAC1***	chr1p34	histone deacetylase 1	0.045	1.2	√										√	
37709_at	8226	*HDHD1*	chrXp22.32	haloacid dehalogenase-like hydrolase domain containing 1	0.046	1.3		√										
31504_at	3069	*HDLBP*	chr2q37.3	high density lipoprotein binding protein	0.003	1.2												
39733_at	9709	*HERPUD1*	chr16q13	homocysteine-inducible, endoplasmic reticulum stress-inducible	0.024	1.8												
32325_at	11112	*HIBADH*	chr7p15	3-hydroxyisobutyrate dehydrogenase	0.010	-1.4												
34696_at	26275	*HIBCH*	chr2q32.2	3-hydroxyisobutyryl-CoA hydrolase	0.040	1.2												
32706_at	7290	*HIRA*	chr22q11.2|22q11.21	HIR histone cell cycle regulation defective homolog A	0.005	1.5												
284_at	8969	*HIST1H2AG*	chr6p22.1	histone cluster 1, H2ag	0.014	-1.3												
31528_f_at	8342	*HIST1H2BM*	chr6p22.1	histone cluster 1, H2bm	0.040	1.2												
40370_f_at	3135	*HLA-G*	chr6p21.3	major histocompatibility complex, class I, G	0.044	1.2						√						
39328_at	3156	*HMGCR*	chr5q13.3-q14	3-hydroxy-3-methylglutaryl-CoA reductase	0.003	1.4												
31402_at	57824	*HMHB1*	chr5q31.3	histocompatibility (minor) HB-1	0.003	-1.3												
33802_at	3162	*HMOX1*	chr22q12|22q13.1	heme oxygenase (decycling) 1	0.000	1.7					√							
40800_at	90861	*HN1L*	chr16p13.3	hematological and neurological expressed 1-like	0.031	-1.2												
34640_at	6927	*HNF1A*	chr12q22-qter|12q24.2	HNF1 homeobox A	0.018	-1.2	√										√	
35916_s_at	220988	*HNRNPA3*	chr2q31.2	heterogeneous nuclear ribonucleoprotein A3	0.045	1.8												
37809_at	3205 /// 3206	*HOXA10 /// HOXA9*	chr7p15.2	homeobox A10 /// homeobox A9	0.011	-1.2												
39476_at	3231	*HOXD1*	2q31.1	homeobox D1	0.043	-1.2												

35384_at	3269	*HRH1*	chr3p25	histamine receptor H1	0.049	-1.3											√	
41555_at	9957	*HS3ST1*	chr4p16	Heparan sulfate (glucosamine) 3-O-sulfotransferase 1	0.026	-1.3												
40778_at	3028	*HSD17B10*	chrXp11.2	hydroxysteroid (17-beta) dehydrogenase 10	0.017	1.3												
202_at	3298	*HSF2*	chr6q22.31	heat shock transcription factor 2	0.041	1.3												
741_g_at	3359	*HTR3A*	chr11q23.1	5-hydroxytryptamine (serotonin) receptor 3A	0.040	-1.3												
34466_at	3360	*HTR4*	chr5q31-q33	5-hydroxytryptamine (serotonin) receptor 4	0.005	-1.4												
37176_at	3373	*HYAL1*	chr3p21.3-p21.2	hyaluronoglucosaminidase 1	0.005	-1.2												√
40758_at	3396	*ICT1*	chr17q25.1	immature colon carcinoma transcript 1	0.016	1.5												
39023_at	3417	*IDH1*	chr2q33.3	isocitrate dehydrogenase 1 (NADP+), soluble	0.023	1.5												
33155_at	3425	*IDUA*	chr4p16.3	iduronidase, alpha-L-	0.044	-1.2												√
425_at	3429	*IFI27*	chr14q32	interferon, alpha-inducible protein 27	0.027	14											√	
925_at	10437	***IFI30***	chr19p13.1	interferon, gamma-inducible protein 30	0.027	2		√									√	
37641_at	10561	*IFI44*	chr1p31.1	interferon-induced protein 44	0.043	7.4											√	
36927_at	10964	*IFI44L*	chr1p31.1	interferon-induced protein 44-like	0.040	10.6											√	
1540_f_at	3442	*IFNA5*	chr9p22	interferon, alpha 5	0.012	-1.3				√		√		√			√	
1038_s_at	3459	*IFNGR1*	chr6q23.3	interferon gamma receptor 1	0.044	1.4				√		√					√	
34718_at	3480	*IGF1R*	chr15q26.3	insulin-like growth factor 1 receptor	0.037	-1.2	√											
1591_s_at	3481 /// 723961	*IGF2 /// INS-IGF2*	chr11p15.5	insulin-like growth factor 2 (somatomedin A)	0.042	-1.3					√							
41420_at	3488	*IGFBP5*	chr2q33-q36	insulin-like growth factor binding protein 5	0.006	-1.2												
1736_at	3489	*IGFBP6*	chr12q13	insulin-like growth factor binding protein 6	0.033	-1.4												
31564_at	3500	*IGHG1*	chr14q32.33	Immunoglobulin heavy constant gamma 1 (G1m marker)	0.003	-1.2											√	
218_at	3550	*IK*	chr2p15-p14|5q31.3	IK cytokine, down-regulator of HLA II	0.049	1.5												
35659_at	3587	***IL10RA***	chr11q23	interleukin 10 receptor, alpha	0.047	1.3				√			√					
41848_f_at	11009	*IL24*	chr1q32	interleukin 24	0.032	-1.2				√								
36755_s_at	3568	*IL5RA*	chr3p26-p24	interleukin 5 receptor, alpha	0.019	-1.3				√								
938_at	3581	*IL9R*	chrXq28 and Yq12	interleukin 9 receptor	0.024	-1.2				√								
41406_at	65123	*INTS3*	chr1q21.3	integrator complex subunit 3	0.047	1.2												
35123_at	10526	*IPO8*	chr12p11.21	importin 8	0.006	-1.2												
32957_g_at	124152	*IQCK*	chr16p12.3	IQ motif containing K	0.020	-1.2												
478_g_at	3663	*IRF5*	chr7q32	interferon regulatory factor 5	0.021	1.4	√							√			√	
41348_at	10265	*IRX5*	chr16q12.2	iroquois homeobox 5	0.032	-1.5												
1978_at	3673	*ITGA2*	chr5q11.2	integrin, alpha 2 (CD49B, alpha 2 subunit of VLA-2 receptor)	0.047	-1.2			√									
37918_at	3689	***ITGB2***	chr21q22.3	integrin, beta 2 (complement component 3 receptor 3 and 4 subunit)	0.049	1.6			√		√	√	√				√	
1176_at	3690	*ITGB3*	chr17q21.32	integrin, beta 3 (platelet glycoprotein IIIa, antigen CD61)	0.031	-1.2			√									
40752_at	113177	*IZUMO4*	chr19p13.3	IZUMO family member 4	0.037	-1.2												
37468_at	3717	*JAK2*	chr9p24	Janus kinase 2	0.047	1.5	√		√	√							√	
36351_at	81621	*KAZALD1*	chr10q24.31	Kazal-type serine peptidase inhibitor domain 1	0.028	-1.1												
32344_r_at	3739	*KCNA4*	chr11p14	potassium voltage-gated channel, shaker-related subfamily, member 4	0.048	-1.2												
32709_at	7881	*KCNAB1*	chr3q26.1	potassium voltage-gated channel, shaker-related subfamily, beta member 1	0.024	1.2												
31312_at	9312	*KCNB2*	chr8q13.2	potassium voltage-gated channel, Shab-related subfamily, member 2	0.005	-1.3												
32356_at	3762	*KCNJ5*	chr11q24	potassium inwardly-rectifying channel, subfamily J, member 5	0.003	-1.3												
40737_at	3778	*KCNMA1*	chr10q22.3	potassium large conductance calcium-activated channel, subfamily M, alpha member 1	0.007	-1.3												
36064_at	3785	*KCNQ2*	chr20q13.3	potassium voltage-gated channel, KQT-like subfamily, member 2	0.032	-1.4												
39479_g_at	154881	*KCTD7*	chr7q11.21	Potassium channel tetramerisation domain containing 7	0.019	-1.2												
41122_at	23028	*KDM1A*	chr1p36.12	lysine (K)-specific demethylase 1A	0.021	1.2	√										√	
40080_at	23065	*KIAA0090*	chr1p36.13	KIAA0090	0.015	-1.2												
35744_at	9812	*KIAA0141*	chr5q31.3	KIAA0141	0.005	1.2												
36970_at	23199	*KIAA0182*	chr16q24.1	KIAA0182	0.031	1.2												
36562_at	9811	*KIAA0427*	chr18q21.1	KIAA0427	0.039	-1.3												
37232_at	9786	*KIAA0586*	chr14q23.1	KIAA0586	0.027	-1.2												
36904_at	547	*KIF1A*	chr2q37.3	kinesin family member 1A	0.024	-1.2												
37171_at	9493	*KIF23*	chr15q23	kinesin family member 23	0.012	-1.1												
31978_at	3834	*KIF25*	chr6q27	kinesin family member 25	0.045	-1.3												
37778_at	22944	*KIN*	chr10p15-p14	KIN, antigenic determinant of recA protein homolog (mouse)	0.022	1.1												
36735_f_at	3811 /// 3812 /// 727787	*KIR3DL1 /// KIR3DL2*	chr19q13.4	killer cell immunoglobulin-like receptor, three domains, long cytoplasmic tail, 1 /// k	0.035	-1.4					√							
246_at	3816	*KLK1*	chr19q13.3	kallikrein 1	0.030	-1.2												
1805_g_at	354	*KLK3*	chr19q13.41	kallikrein-related peptidase 3	0.024	-1.4												
37554_at	5653	*KLK6*	chr19q13.3	kallikrein-related peptidase 6	0.045	-1.2												
32297_s_at	3821 /// 3822	***KLRC1 /// KLRC2***	chr12p13	killer cell lectin-like receptor subfamily C, member 1 /// killer cell lectin-like rece	0.017	-2.3						√					√	
32287_s_at	3821 /// 3822	***KLRC1 /// KLRC2 /// KLRC3***	chr12p13	killer cell lectin-like receptor subfamily C, member 1 /// killer cell lectin-like rece	0.026	-2.8						√						
32288_r_at	3823	*KLRC3*	chr12p13	killer cell lectin-like receptor subfamily C, member 3	0.047	-1.7												
40407_at	3838	*KPNA2*	chr17q24.2	karyopherin alpha 2 (RAG cohort 1, importin alpha 1)	0.003	1.6	√										√	
35085_r_at	889	*KRIT1*	chr7q21.2	KRIT1, ankyrin repeat containing	0.048	1.1												
35766_at	3875	*KRT18*	chr12q13	keratin 18	0.007	-1.3												
34568_at	3884	*KRT33B*	chr17q21.2	keratin 33B	0.013	-1.3												
34244_r_at	26013	***L3MBTL1***	chr20q13.12	l(3)mbt-like 1 (Drosophila)	0.047	-1.2	√											
40891_f_at	8270	*LAGE3*	chrXq28	L antigen family, member 3	0.040	1.1												
36917_at	3908	*LAMA2*	chr6q22-q23	laminin, alpha 2	0.017	-1.3			√									
41610_at	3911	***LAMA5***	chr20q13.2-q13.3	laminin, alpha 5	0.030	-1.3			√									
232_at	3915	*LAMC1*	chr1q31	laminin, gamma 1 (formerly LAMB2)	0.001	-1.2			√									
41485_at	3939	*LDHA*	chr11p15.4	lactate dehydrogenase A	0.003	1.8												
32855_at	3949	*LDLR*	chr19p13.3	low density lipoprotein receptor	0.006	1.9												
33412_at	3956	*LGALS1*	chr22q13.1	lectin, galactoside-binding, soluble, 1	0.025	2.1												
37456_at	3957	***LGALS2***	chr22q12-q13|22q13.1	lectin, galactoside-binding, soluble, 2	0.012	2												
37754_at	3959	***LGALS3BP***	chr17q25	lectin, galactoside-binding, soluble, 3 binding protein	0.002	2.8			√									
38091_at	3965	***LGALS9***	chr17q11.2	lectin, galactoside-binding, soluble, 9	0.037	1.5												
35378_at	3972	*LHB*	chr19q13.32	luteinizing hormone beta polypeptide	0.038	-1.2												
37542_at	10184	***LHFPL2***	chr5q14.1	lipoma HMGIC fusion partner-like 2	0.010	1.8												
32475_at	11024	*LILRA1*	chr19q13.4	leukocyte immunoglobulin-like receptor, subfamily A (with TM domain), member 1	0.047	1.5												
36753_at	11006	***LILRB4***	chr19q13.4	leukocyte immunoglobulin-like receptor, subfamily B (with TM and ITIM domains)	0.019	2.1											√	
39287_at	100131825	*LOC100131825*	chr1q23.2	hypothetical LOC100131825	0.032	-1.3												
35635_at	92249	*LOC92249*	chrXq11.1	hypothetical LOC92249	0.008	-1.3												
39245_at	10162	***LPCAT3***	chr12p13	Lysophosphatidylcholine acyltransferase 3	0.023	-1.1												
31659_at	26103	*LRIT1*	chr10q23	leucine-rich repeat, immunoglobulin-like and transmembrane domains 1	0.004	-1.4												
34409_at	26020	*LRP10*	chr14q11.2	low density lipoprotein receptor-related protein 10	0.047	-1.3												

36194_at	4043	*LRPAP1*	chr4p16.3	low density lipoprotein receptor-related protein associated protein 1	0.015	1.4												
39017_at	27257	***LSM1***	chr8p11.2	LSM1 homolog, U6 small nuclear RNA associated (S. cerevisiae)	0.020	1.3												
32547_at	4074	*M6PR*	chr12p13	mannose-6-phosphate receptor (cation dependent)	0.028	1.3												√
1721_g_at	4085	*MAD2L1*	chr4q27	MAD2 mitotic arrest deficient-like 1 (yeast)	0.004	1.2												
32270_g_at	9223	*MAGI1*	chr3p14.1	membrane associated guanylate kinase, WW and PDZ domain containing 1	0.005	-1.3			√									
40036_at	4116	*MAGOH*	chrXp11.23	mago-nashi homolog, proliferation-associated (Drosophila)	0.042	1.8												
37628_at	4129	***MAOB***	chr3p21	monoamine oxidase B	0.043	-1.3		√										
32226_at	4134	*MAP4*	chr3p21.3	microtubule-associated protein 4	0.036	1.2												
1637_at	7867	*MAPKAPK3*	chr14q32.3	mitogen-activated protein kinase-activated protein kinase 3	0.044	1.4												
40826_at	4140	*MARK3*	chr12q13.2	MAP/microtubule affinity-regulating kinase 3	0.027	1.2												
39342_at	4141	*MARS*	chr5q12.3	methionyl-tRNA synthetase	0.018	1.3												
40015_at	375449	*MAST4*	chr20q13.1-q13.2	microtubule associated serine/threonine kinase family member 4	0.047	-1.3												
32925_at	8785	*MATN4*	chr2p25.1	matrilin 4	0.022	-1.3												
38262_at	129642	*MBOAT2*	chr5q21	membrane bound O-acyltransferase domain containing 2	0.004	-1.3												
35561_at	4163	*MCC*	chr15q15-q21	mutated in colorectal cancers	0.038	-1.2												
36535_at	4236	*MFAP1*	chr15q25	microfibrillar-associated protein 1	0.005	1.4												
34403_at	4240	*MFGE8*	chr1p36.22	milk fat globule-EGF factor 8 protein	0.036	-1.3			√									
34369_at	9927	*MFN2*	chr4p16.3	mitofusin 2	0.004	1.5	√										√	
32080_at	10227	*MFSD10*	chr12q24.31	major facilitator superfamily domain containing 10	0.032	1.2												
35444_at	126353	*MISP*	chr19p13.3	mitotic spindle positioning	0.036	-1.2												
41728_at	9761	*MLEC*	chr17q21.1	malectin	0.048	1.2												
35608_at	653483	*MLLT4-AS1*	chr6q27	MLLT4 antisense RNA 1 (head to head)	0.028	-1.2												
32578_at	6945	*MLX*	chr2p13.1	MAX-like protein X	0.048	1.3												
37734_at	55233	*MOBKL1B*	chr13q12.11	MOB1, Mps One Binder kinase activator-like 1B (yeast)	0.026	1.4												
35110_at	54737	*MPHOSPH8*	chr4p16.1	M-phase phosphoprotein 8	0.008	-1.2												
35356_at	114932	*MRFAP1L1*	chr2q11.1-q11.2	Morf4 family associated protein 1-like 1	0.028	1.4												
37174_at	9801	*MRPL19*	chr15q25	mitochondrial ribosomal protein L19	0.040	1.4												
39004_at	64963	*MRPS11*	chrXq11.1	mitochondrial ribosomal protein S11	0.023	1.2		√										
40771_at	4478	*MSN*	chr10q26.3	moesin	0.033	1.2			√		√							
38623_at	92170	*MTG1*	chr14q24	mitochondrial GTPase 1 homolog (S. cerevisiae)	0.015	-1.2												
674_g_at	4522	*MTHFD1*	chr1q12-q21	methylenetetrahydrofolate dehydrogenase (NADP+ dependent) 1	0.042	1.6												
1229_at	10903	*MTMR11*	chr8p22	myotubularin related protein 11	0.029	1.4												
38013_at	57509	*MTUS1*	chr1q21	microtubule associated tumor suppressor 1	0.024	-1.2												
38783_at	4582	***MUC1***	chr11p15.5	mucin 1, cell surface associated	0.020	-1.2	√						√				√	
733_at	4583	*MUC2*	chr9q31.3-q32	mucin 2, oligomeric mucus/gel-forming	0.030	-1.6												
31777_at	4593	*MUSK*	chr1p34.1	muscle, skeletal, receptor tyrosine kinase	0.029	-1.2												
490_g_at	4595	*MUTYH*	chr12q23.2	mutY homolog (E. coli)	0.014	1.2												
40503_at	4604	*MYBPC1*	chr19q13.33	myosin binding protein C, slow type	0.044	-1.2			√									
37941_at	4606	*MYBPC2*	chr16p13.11	myosin binding protein C, fast type	0.040	-1.3			√									
773_at	4629	*MYH11*	chr3q13.13	myosin, heavy chain 11, smooth muscle	0.039	-1.3												
31652_at	22989	*MYH15*	chr12q13.13	myosin, heavy chain 15	0.020	-1.1												
38251_at	140465	*MYL6B*	chr17q11-q12	myosin, light chain 6B, alkali, smooth muscle and non-muscle	0.046	1.1												
35729_at	4642	*MYO1D*	chr10q24	myosin ID	0.022	-1.1												
34678_at	26509	*MYOF*	chr22q13-qter|22q11	myoferlin	0.028	2												
36607_at	4668	*NAGA*	22q13.2	N-acetylgalactosaminidase, alpha-	0.023	1.4												√
32931_at	4684	*NCAM1*	11q23.2	neural cell adhesion molecule 1	0.005	-1.2			√									
32642_at	1463	*NCAN*	chr19p12	neurocan	0.019	-1.3			√									
38309_r_at	23154	*NCDN*	chr1p34.3	neurochondrin	0.022	-1.3												
32590_at	4691	*NCL*	chr2q37.1	nucleolin	0.047	1.4	√										√	
39358_at	9612	***<u>NCOR2</u>***	chr12q24	nuclear receptor corepressor 2	0.015	1.9	√										√	
39077_at	125144	*NCRNA00188*	chr17p11.2	non-protein coding RNA 188	0.046	-1.4												
40041_at	10403	***NDC80***	chr18p11.32	NDC80 homolog, kinetochore complex component (S. cerevisiae)	0.019	1.7												
31500_at	10397	*NDRG1*	chr8q24	N-myc downstream regulated 1	0.003	-1.3					√							
41411_at	51103	*NDUFAF1*	chr15q11.2-q21.3	NADH dehydrogenase (ubiquinone) 1 alpha subcomplex, assembly factor 1	0.040	1.5		√										
35264_at	4722	*NDUFS3*	chr11p11.11	NADH dehydrogenase (ubiquinone) Fe-S protein 3	0.030	1.4												
32078_at	4734	*NEDD4*	chr15q	neural precursor cell expressed, developmentally down-regulated 4	0.029	-1.2	√											
41407_at	7936	*NELFE*	chr6p21.3	negative elongation factor complex member E	0.016	1.2												
250_at	4775	*NFATC3*	chr16q22.2	nuclear factor of activated T-cells, cytoplasmic, calcineurin-dependent 3	0.002	1.2	√					√			√	√		
1377_at	4790	*NFKB1*	chr4q24	nuclear factor of kappa light polypeptide gene enhancer in B-cells 1	0.022	1.4	√			√				√	√	√	√	
35366_at	4811	*NID1*	chr1q43	nidogen 1	0.009	1.3			√									
33598_r_at	114548	*NLRP3*	chr1q44	NLR family, pyrin domain containing 3	0.012	1.3	√											
1521_at	4830	*NME1*	chr17q21.3	non-metastatic cells 1, protein (NM23A) expressed in	0.008	1.2			√									
37032_at	4837	***NNMT***	chr11q23.1	nicotinamide N-methyltransferase	0.024	-1.1												
576_at	4846	*NOS3*	chr7q36	nitric oxide synthase 3 (endothelial cell)	0.028	-1.2												
35663_at	4885	*NPTX2*	chr7q21.3-q22.1	neuronal pentraxin II	0.002	-1.2												
36836_at	8829	*NRP1*	chr10p12	neuropilin 1	0.006	-1.3			√									
38719_at	4905	*NSF*	chr17q21	N-ethylmaleimide-sensitive factor	0.050	1.4												
35041_at	4908	*NTF3*	chr12p13	neurotrophin 3	0.037	-1.4												
40325_at	4913	*NTHL1*	chr16p13.3	nth endonuclease III-like 1 (E. coli)	0.049	-1.2												
1059_at	4916	*NTRK3*	chr15q25	neurotrophic tyrosine kinase, receptor, type 3	0.049	-1.1												
33787_at	9891	*NUAK1*	chr12q23.3	NUAK family, SNF1-like kinase, 1	0.024	-1.2												
39142_at	11051	*NUDT21*	chr16q12.2	nudix (nucleoside diphosphate linked moiety X)-type motif 21	0.044	1.5												
39390_at	55746	*NUP133*	chr1q42.13	nucleoporin 133kDa	0.047	1.4												
33297_at	221443	*OARD1*	chr6p21.1	O-acyl-ADP-ribose deacylase 1	0.018	1.4												
38389_at	4938	***OAS1***	chr12q24.1	2',5'-oligoadenylate synthetase 1, 40/46kDa	0.001	6	√										√	
39264_at	4939	***OAS2***	chr12q24.2	2'-5'-oligoadenylate synthetase 2, 69/71kDa	0.007	1.9											√	
36203_at	4953	***ODC1***	chr2p25	ornithine decarboxylase 1	0.014	1.5												
34065_at	9480	*ONECUT2*	18q21.31	one cut homeobox 2	0.029	-1.3			√								√	
36043_at	4978	*OPCML*	chr11q25	opioid binding protein/cell adhesion molecule-like	0.026	-1.4			√									
35024_at	4986	*OPRK1*	chr8q11.2	opioid receptor, kappa 1	0.005	-1.2												
31350_at	26532	*OR10H3*	chr19p13.1	olfactory receptor, family 10, subfamily H, member 3	0.020	-1.3												
31428_at	10798	*OR5I1*	chr11q11	olfactory receptor, family 5, subfamily I, member 1	0.011	-1.2												
32007_at	23762	*OSBP2*	chr22q12.2	oxysterol binding protein 2	0.030	-1.2												
38723_at	55644	*OSGEP*	chr14q11.2	O-sialoglycoprotein endopeptidase	0.043	1.3												
32445_at	5049	*PAFAH1B2*	chr11q23	platelet-activating factor acetylhydrolase 1b, catalytic subunit 2 (30kDa)	0.050	-1.3												

35874_at	5051	*PAFAH2*	chr1p36	platelet-activating factor acetylhydrolase 2, 40kDa	0.045	-1.2												
36367_at	27328 /// 83259	*PCDH11X /// PCDH11Y*	chrXq21.3 /// chrYp11.2	protocadherin 11 X-linked /// protocadherin 11 Y-linked	0.037	-1.1												
38588_at	5145	*PDE6A*	chr5q31.2-q34	Phosphodiesterase 6A, cGMP-specific, rod, alpha	0.015	-1.3												
35714_at	8566	*PDXK*	chr21q22.3	pyridoxal (pyridoxine, vitamin B6) kinase	0.010	1.4												
37111_g_at	5209	*PFKFB3*	chr10p15.1	6-phosphofructo-2-kinase/fructose-2,6-biphosphatase 3	0.006	-1.2												
33962_at	5251	*PHEX*	chrXp22.2-p22.1	phosphate regulating endopeptidase homolog, X-linked	0.011	-1.2												
39117_at	5253	*PHF2*	chr9q22.31	PHD finger protein 2	0.045	1.5											√	
776_at	5281	*PIGF*	chr2p21-p16	phosphatidylinositol glycan anchor biosynthesis, class F	0.045	1.7												
35665_at	5289	*PIK3C3*	chr18q12.3	phosphoinositide-3-kinase, class 3	0.033	1.4												
34839_at	10531	*PITRM1*	chr10p15.2	pitrilysin metallopeptidase 1	0.014	1.5												
460_at	5316	*PKNOX1*	chr21q22.3	PBX/knotted 1 homeobox 1	0.021	-1.2	√				√				√			
33475_at	8502	*PKP4*	chr2q24.1	plakophilin 4	0.048	-1.2			√									
37068_at	7941	*PLA2G7*	chr6p21.2-p12	phospholipase A2, group VII (platelet-activating factor acetylhydrolase, plasma)	0.021	1.4												
40061_at	5326	*PLAGL2*	chr20q11.21	pleiomorphic adenoma gene-like 2	0.036	1.7												
37310_at	5328	***PLAU***	chr10q24	plasminogen activator, urokinase	0.007	-1.3							√					
364_s_at	5331	*PLCB3*	chr11q13	phospholipase C, beta 3 (phosphatidylinositol-specific)	0.043	-1.2				√								
36151_at	23646	*PLD3*	chr19q13.2	phospholipase D family, member 3	0.023	1.5												
33807_at	22874	*PLEKHA6*	chr1q32.1	pleckstrin homology domain containing, family A member 6	0.035	-1.2												
41253_s_at	55041	*PLEKHB2*	chr2q21.1	pleckstrin homology domain containing, family B (evectins) member 2	0.016	1.4		√										
40959_at	26030	*PLEKHG3*	chr14q23.3	pleckstrin homology domain containing, family G (with RhoGef domain) member 3	0.032	-1.3												
34378_at	123	***PLIN2***	chr9p22.1	perilipin 2	0.044	1.5												
32775_r_at	5359	***PLSCR1***	chr3q23	phospholipid scramblase 1	0.042	2.6	√										√	
40730_at	5368	*PNOC*	chr8p21	prepronociceptin	0.005	-1.1												
40434_at	5420	***PODXL***	chr7q32-q33	podocalyxin-like	0.021	-1.3			√		√							
31747_g_at	94026	*POM121L2*	chr6p22.1	POM121 membrane glycoprotein-like 2	0.020	-1.4												
38380_at	10775	*POP4*	chr19q12	processing of precursor 4, ribonuclease P/MRP subunit (S. cerevisiae)	0.010	1.3												
34551_at	5458	*POU4F2*	chr4q31.2	POU class 4 homeobox 2	0.024	-1.2												
37848_at	8496	*PPFIBP1*	chr12p12.1	PTPRF interacting protein, binding protein 1 (liprin beta 1)	0.046	-1.1			√									
34366_g_at	10450	*PPIE*	chr1p32	peptidylprolyl isomerase E (cyclophilin E)	0.031	1.3												
38352_at	10465	*PPIH*	chr1p34.1	peptidylprolyl isomerase H (cyclophilin H)	0.006	1.3												
34925_at	4660	*PPP1R12B*	chr1q32.1	protein phosphatase 1, regulatory (inhibitor) subunit 12B	0.045	-1.3												
39364_s_at	5507	*PPP1R3C*	chr10q23-q24	protein phosphatase 1, regulatory (inhibitor) subunit 3C	0.042	-1.1												
41540_at	5510	*PPP1R7*	chr2q37.3	protein phosphatase 1, regulatory (inhibitor) subunit 7	0.032	1.5												
1384_at	5521	*PPP2R2B*	chr5q32	protein phosphatase 2, regulatory subunit B, beta	0.038	-1.2												
40784_at	5527	*PPP2R5C*	chr14q32.31	protein phosphatase 2, regulatory subunit B', gamma	0.043	1.4												
34774_at	5538	*PPT1*	chr1p32	palmitoyl-protein thioesterase 1	0.032	1.7												√
33922_at	7799	*PRDM2*	chr1p36.21	PR domain containing 2, with ZNF domain	0.014	-1.4												
41213_at	5052	*PRDX1*	chr1p34.1	peroxiredoxin 1	0.012	1.7	√											
31634_at	5554	*PRH1*	chr12p13.2	proline-rich protein HaeIII subfamily 1	0.003	-1.2												
34346_at	5571	*PRKAG1*	chr12q12-q14	protein kinase, AMP-activated, gamma 1 non-catalytic subunit	0.019	1.2												
362_at	5590	*PRKCZ*	chr1p36.33-p36.2	protein kinase C, zeta	0.006	-1.3	√		√	√							√	
31543_at	58503	*PROL1*	chr4q13.3	proline rich, lacrimal 1	0.030	-1.3												
38248_at	9762	*ProSAPiP1*	chr20p13	ProSAPiP1 protein	0.021	-1.2												
31918_at	5629	*PROX1*	chr1q41	prospero homeobox 1	0.026	-1.2											√	
40269_at	8559	*PRPF18*	chr10p13	PRP18 pre-mRNA processing factor 18 homolog (S. cerevisiae)	0.031	1.3												
39675_at	5639	*PRRG2*	chr19q13.33	proline rich Gla (G-carboxyglutamic acid) 2	0.045	-1.3												
40206_at	5681	*PSKH1*	chr16q22.1	protein serine kinase H1	0.047	-1.3												
41240_at	5683	*PSMA2*	chr7p13	proteasome (prosome, macropain) subunit, alpha type, 2	0.023	1.1	√											√
1449_at	5685	*PSMA4*	chr15q25.1	proteasome (prosome, macropain) subunit, alpha type, 4	0.017	1.7	√											√
37046_at	5686	*PSMA5*	chr1p13	proteasome (prosome, macropain) subunit, alpha type, 5	0.013	1.6	√											√
36122_at	5687	*PSMA6*	chr14q13	proteasome (prosome, macropain) subunit, alpha type, 6	0.013	1.9	√											√
1310_at	5690	*PSMB2*	chr1p34.2	proteasome (prosome, macropain) subunit, beta type, 2	0.009	1.5	√											√
941_at	5694	*PSMB6*	chr17p13	proteasome (prosome, macropain) subunit, beta type, 6	0.025	1.2	√											√
1313_at	5695	*PSMB7*	chr9q34.11-q34.12	proteasome (prosome, macropain) subunit, beta type, 7	0.008	1.6	√											√
41184_s_at	5696	***PSMB8***	chr6p21.3	proteasome (prosome, macropain) subunit, beta type, 8	0.019	1.4												√
688_at	5700	*PSMC1*	chr14q32.11	proteasome (prosome, macropain) 26S subunit, ATPase, 1	0.019	1.2												√
592_at	5702	*PSMC3*	chr11p11.2	proteasome (prosome, macropain) 26S subunit, ATPase, 3	0.008	1.3												√
32848_at	5704	*PSMC4*	chr19q13.11-q13.13	proteasome (prosome, macropain) 26S subunit, ATPase, 4	0.005	1.3												√
1314_at	5707	*PSMD1*	chr2q37.1	proteasome (prosome, macropain) 26S subunit, non-ATPase, 1	0.013	1.3												√
1191_s_at	5717	*PSMD11*	chr17q11.2	proteasome (prosome, macropain) 26S subunit, non-ATPase, 11	0.038	1.4												√
40276_at	5713	*PSMD7*	chr16q22.3	proteasome (prosome, macropain) 26S subunit, non-ATPase, 7	0.029	1.4												√
1312_at	5714	*PSMD8*	chr19q13.2	proteasome (prosome, macropain) 26S subunit, non-ATPase, 8	0.003	1.5		√										√
38406_f_at	5730	*PTGDS*	chr9q34.2-q34.3	prostaglandin D2 synthase 21kDa (brain)	0.037	-1.2												
38209_at	5731	*PTGER1*	chr19p13.1	prostaglandin E receptor 1 (subtype EP1), 42kDa	0.007	-1.3												
36117_at	5747	*PTK2*	chr8q24.3	PTK2 protein tyrosine kinase 2	0.033	1.2				√								
1496_at	5786	*PTPRA*	chr20p13	protein tyrosine phosphatase, receptor type, A	0.045	1.4												
37777_at	5787	*PTPRB*	chr12q15-q21	protein tyrosine phosphatase, receptor type, B	0.036	-1.2												
994_at	5797	*PTPRM*	chr18p11.2	protein tyrosine phosphatase, receptor type, M	0.032	-1.2			√									
41345_at	5813	*PURA*	chr5q31	Purine-rich element binding protein A	0.033	-1.3	√											
40816_at	11137	*PWP1*	chr12q23.3	PWP1 homolog (S. cerevisiae)	0.044	1.3												
38644_at	5829	*PXN*	chr12q24.31	paxillin	0.039	-1.2	√		√	√							√	
33599_at	27314	*RAB30*	chr11q12-q14	RAB30, member RAS oncogene family	0.005	-1.2												
35508_at	11021	*RAB35*	chr12q24.31	RAB35, member RAS oncogene family	0.022	-1.1												
37362_at	5869	*RAB5B*	chr12q13	RAB5B, member RAS oncogene family	0.007	-1.2												
781_at	5876	*RABGGTB*	chr1p31	Rab geranylgeranyltransferase, beta subunit	0.035	1.7		√										
35152_at	10268	***RAMP3***	chr7p13-p12	receptor (G protein-coupled) activity modifying protein 3	0.012	-1.2												
36240_at	2889	*RAPGEF1*	chr9q34.3	Rap guanine nucleotide exchange factor (GEF) 1	0.045	-1.3												
1381_at	5915	*RARB*	chr3p24	retinoic acid receptor, beta	0.048	-1.3	√											
1042_at	5918	*RARRES1*	chr3q25.32	retinoic acid receptor responder (tazarotene induced) 1	0.043	-1.2												
549_at	5917	*RARS*	chr5q35.1	arginyl-tRNA synthetase	0.016	1.2												
1905_s_at	5925	*RB1*	chr13q14.2	retinoblastoma 1	0.016	-1.2	√										√	
41265_at	83759	*RBM4B*	chr11q13	RNA binding motif protein 4B	0.047	-1.3												
40819_at	9939	*RBM8A*	chr1q12	RNA binding motif protein 8A	0.012	-1.3												
333_s_at	5937	*RBMS1*	chr2q24.2	RNA binding motif, single stranded interacting protein 1	0.012	1.4	√											
36527_at	51634	*RBMX2*	chrXq26.1	RNA binding motif protein, X-linked 2	0.030	1.2												

680_s_at	5949	*RBP3*	chr10q11.2	retinol binding protein 3, interstitial	0.031	-1.2												
34162_at	11030	*RBPMS*	chr8p12	RNA binding protein with multiple splicing	0.022	-1.2												
1271_g_at	5970	***RELA***	chr11q13	v-rel reticuloendotheliosis viral oncogene homolog A (avian)	0.008	-1.2	√			√		√	√	√	√	√	√	
39157_at	10739	*RFPL2*	chr22q12.3	ret finger protein-like 2; ret finger protein-like 3	0.019	-1.2												
37396_at	8625	*RFXANK*	chr19p12	regulatory factor X-associated ankyrin-containing protein	0.049	-1.2												
37539_at	23179	***RGL1***	chr1q25.3	ral guanine nucleotide dissociation stimulator-like 1	0.048	1.4												
37309_at	387	*RHOA*	chr3p21.3	ras homolog gene family, member A	0.018	1.9			√	√								
36550_at	54453	*RIN2*	chr20p11.22	Ras and Rab interactor 2	0.036	2.9												
31352_at	6035	*RNASE1*	chr14q11.2	ribonuclease, RNase A family, 1 (pancreatic)	0.031	-1.3												
36766_at	6036	*RNASE2*	chr14q24-q31	ribonuclease, RNase A family, 2 (liver, eosinophil-derived neurotoxin)	0.007	2												
33979_at	6037	*RNASE3*	chr14q24-q31	ribonuclease, RNase A family, 3	0.022	-1.3												
32664_at	6038	***RNASE4***	chr14q11.1	ribonuclease, RNase A family, 4	0.045	1.4												
34660_at	6039	***RNASE6***	chr14q11.2	ribonuclease, RNase A family, k6	0.003	1.7												
35212_at	11236	*RNF139*	chr8q24	ring finger protein 139	0.016	1.2												
31661_at	6048	*RNF5*	chr6p21.3	ring finger protein 5	0.006	-1.3											√	
39496_s_at	84900	*RNFT2*	chr12q24.22	Ring finger protein, transmembrane 2	0.005	-1.2		√										
36187_at	6050	*RNH1*	chr11p15.5	ribonuclease/angiogenin inhibitor 1	0.036	1.4												
652_g_at	6119	*RPA3*	chr7p22	replication protein A3, 14kDa	0.045	1.2												
31370_at	57096	*RPGRIP1*	chr14q11.2	retinitis pigmentosa GTPase regulator interacting protein 1	0.002	-1.2												
41214_at	6192	*RPS4Y1*	chrYp11.3	ribosomal protein S4, Y-linked 1	0.048	-1.3												
32892_at	6196	***RPS6KA2***	chr6q27	ribosomal protein S6 kinase, 90kDa, polypeptide 2	0.041	-1.2												
722_at	9125	*RQCD1*	chr2q35	RCD1 required for cell differentiation1 homolog (S. pombe)	0.048	-1.2												
35316_at	10670	*RRAGA*	chr9p22.1	Ras-related GTP binding A	0.023	1.4		√										
38338_at	6237	*RRAS*	chr19q13.3-qter	related RAS viral (r-ras) oncogene homolog	0.003	1.3	√											
1838_g_at	22800	*RRAS2*	chr11p15.2	related RAS viral (r-ras) oncogene homolog 2	0.046	-1.2												
34314_at	6240	*RRM1*	chr11p15.5	ribonucleotide reductase M1	0.021	1.4												
38689_at	51493	*RTCB*	chr22q12	RNA 2',3'-cyclic phosphate and 5'-OH ligase	0.022	1.6												
34408_at	6253	*RTN2*	chr19q13.32	reticulon 2	0.049	-1.2												
35638_at	862	*RUNX1T1*	chr8q22	runt-related transcription factor 1; translocated to, 1 (cyclin D-related)	0.049	-1.2	√										√	
37990_at	6261	*RYR1*	chr19q13.1	ryanodine receptor 1 (skeletal)	0.036	-1.4												
34458_at	6278	*S100A7*	chr1q21	S100 calcium binding protein A7	0.033	-1.2												
37567_at	6297	*SALL2*	chr14q11.1-q12	sal-like 2 (Drosophila)	0.043	-1.4	√											
41451_s_at	56681	*SAR1A*	chr10q22.1	SAR1 homolog A (S. cerevisiae)	0.024	1.3												
38516_at	6324	*SCN1B*	chr19q13.1	sodium channel, voltage-gated, type I, beta	0.040	-1.1												
34462_at	6339	*SCNN1D*	chr1p36.3-p36.2	sodium channel, nonvoltage-gated 1, delta	0.008	-1.1												
34826_at	6389	*SDHA*	chr5p15	succinate dehydrogenase complex, subunit A, flavoprotein (Fp)	0.045	1.3												
35751_at	6390	*SDHB*	chr1p36.1-p35	succinate dehydrogenase complex, subunit B, iron sulfur (Ip)	0.028	1.4												
39139_at	23478	*SEC11A*	chr15q25.3	SEC11 homolog A (S. cerevisiae)	0.042	1.4												
32817_at	23541	***SEC14L2***	chr22q12.2	SEC14-like 2 (S. cerevisiae)	0.038	-1.3												
34921_at	9717	*SEC14L5*	chr16p13.3	SEC14-like 5 (S. cerevisiae)	0.049	-1.2												
36061_at	9037	***SEMA5A***	chr5p15.2	sema domain, seven thrombospondin repeats (type 1 and type 1-like)	0.047	-1.2			√									
33172_at	55752	*SEPT11*	chr4q21.1	septin 11	0.019	-1.2												
32056_at	5414	*SEPT4*	chr17q23	septin 4	0.006	-1.2	√											
33332_at	253190	*SERHL2*	chr22q13.2	serine hydrolase-like /// serine hydrolase-like 2	0.036	-1.2												
33305_at	1992	***SERPINB1***	6p25.2	serpin peptidase inhibitor, clade B (ovalbumin), member 1	0.035	1.5												
862_at	5268	*SERPINB5*	chr18q21.3	serpin peptidase inhibitor, clade B (ovalbumin), member 5	0.044	-1.2												
40083_at	23064	*SETX*	chr9q34.13	senataxin	0.015	1.5												
37379_at	10946	*SF3A3*	chr1p34.3	splicing factor 3a, subunit 3, 60kDa	0.023	1.2		√										
41199_s_at	6421	*SFPQ*	chr1p34.3	splicing factor proline/glutamine-rich	0.010	1.5												
38691_s_at	6440	*SFTPC*	chr8p21	surfactant protein C	0.007	-1.2												
34992_g_at	6444	*SGCD*	chr5q33-q34	sarcoglycan, delta (35kDa dystrophin-associated glycoprotein)	0.016	-1.4												
36039_s_at	6450	*SH3BGR*	chr21q22.3	SH3 domain binding glutamic acid-rich protein	0.006	-1.2		√										
38118_at	6464	*SHC1*	chr1q21	SHC (Src homology 2 domain containing) transforming protein 1	0.014	1.3				√		√					√	
38744_at	7979	*SHFM1*	chr7q21.3	split hand/foot malformation (ectrodactyly) type 1	0.015	1.9												√
40004_at	6495	*SIX1*	chr14q23.1	SIX homeobox 1	0.032	-1.4											√	
41557_at	23517	*SKIV2L2*	chr5q11.2	superkiller viralicidic activity 2-like 2 (S. cerevisiae)	0.031	1.3												
1941_at	6502	*SKP2*	chr5p13	S-phase kinase-associated protein 2 (p45)	0.009	-1.2	√										√	
36362_at	6559	***SLC12A3***	chr16q13	solute carrier family 12 (sodium/chloride transporters), member 3	0.030	-1.3												
38624_at	6560	*SLC12A4*	chr16q22.1	solute carrier family 12 (potassium/chloride transporters), member 4	0.028	-1.4												
32906_at	6564	***SLC15A1***	chr13q33-q34	solute carrier family 15 (oligopeptide transporter), member 1	0.036	-1.2												
928_at	1811	***SLC26A3***	chr7q31	solute carrier family 26, member 3	0.026	-1.1												
34057_at	9153	*SLC28A2*	chr15q15	solute carrier family 28 (sodium-coupled nucleoside transporter), member 2	0.042	-1.2												
34749_at	1318	*SLC31A2*	chr9q31-q32	solute carrier family 31 (copper transporters), member 2	0.024	1.7												
40922_at	206358	*SLC36A1*	chr5q33.1	Solute carrier family 36 (proton/amino acid symporter), member 1	0.006	-1.2												
41305_at	6524	*SLC5A2*	chr16p12-p11	solute carrier family 5 (sodium/glucose cotransporter), member 2	0.046	-1.4												
34167_s_at	6534	*SLC6A7*	chr5q31-q32	solute carrier family 6 (neurotransmitter transporter, L-proline), member 7	0.010	-1.2												
33731_at	9056	***SLC7A7***	chr14q11.2	solute carrier family 7 (cationic amino acid transporter, y+ system), member 7	0.036	1.5												
39634_at	9353	*SLIT2*	chr4p15.2	slit homolog 2 (Drosophila)	0.032	-1.5												
33576_at	26050	*SLITRK5*	chr13q31.2	SLIT and NTRK-like family, member 5	0.005	-1.2												
453_at	6601	*SMARCC2*	chr12q13.2	SWI/SNF related, matrix associated, actin dependent regulator of chromatin, subfamily c	0.027	-1.1											√	
37753_at	6602	*SMARCD1*	chr12q13-q14	SWI/SNF related, matrix associated, actin dependent regulator of chromatin, subfamily d	0.024	-1.4											√	
35546_at	8243	*SMC1A*	chrXp11.22-p11.21	structural maintenance of chromosomes 1A	0.010	1.2	√											
34878_at	10051	*SMC4*	chr3q26.1	structural maintenance of chromosomes 4	0.014	1.4												
41107_at	9751	*SNPH*	chr20p13	syntaphilin	0.018	-1.3												
41029_at	11066	*SNRNP35*	chr12q24.31	small nuclear ribonucleoprotein 35kDa (U11/U12)	0.031	1.2												
38455_at	6628	*SNRPB*	chr20p13	small nuclear ribonucleoprotein polypeptides B and B1	0.020	1.6												
31654_at	22986	*SORCS3*	chr10q23-q25	sortilin-related VPS10 domain containing receptor 3	0.007	-1.3												
33108_i_at	6657	*SOX2*	chr3q26.3-q27	SRY (sex determining region Y)-box 2	0.029	-1.3	√										√	
32120_at	10615	*SPAG5*	chr17q11.2	sperm associated antigen 5	0.001	1.3												
40615_at	80208	*SPG11*	chr15q14	spastic paraplegia 11 (autosomal recessive)	0.039	1.8												
35839_at	6713	*SQLE*	chr8q24.1	squalene epoxidase	0.025	1.2		√										
38904_at	6714	*SRC*	chr20q12-q13	v-src sarcoma (Schmidt-Ruppin A-2) viral oncogene homolog (avian)	0.042	-1.3	√		√								√	
36069_at	23380	*SRGAP2*	chr1q32.1	SLIT-ROBO Rho GTPase activating protein 2	0.033	1.7												
31855_at	8406	*SRPX*	chrXp21.1	sushi-repeat-containing protein, X-linked	0.029	-1.3			√									
36099_at	6426	*SRSF1*	chr17q22	serine/arginine-rich splicing factor 1	0.041	1.3												

37782_at	6750	*SST*	chr3q28	somatostatin	0.020	-1.4											√	
34068_f_at	6756	*SSX1*	chrXp11.23	synovial sarcoma, X breakpoint 1	0.042	-1.2												
31462_f_at	6758	*SSX5*	chrXp11.23	synovial sarcoma, X breakpoint 5	0.026	-1.2												
38146_at	9705	*ST18*	chr8q11.23	suppression of tumorigenicity 18 (breast carcinoma) (zinc finger protein)	0.045	-1.2												
32942_at	29906	*ST8SIA5*	chr18q21.1	ST8 alpha-N-acetyl-neuraminide alpha-2,8-sialyltransferase 5	0.049	-1.1												
39391_at	10617	*STAMBP*	chr2p13.1	STAM binding protein	0.040	1.4												
32859_at	6772	***STAT1***	chr2q32.2	signal transducer and activator of transcription 1, 91kDa	0.043	2.2	√			√		√		√			√	
1244_at	6773	*STAT2*	chr12q13.3	signal transducer and activator of transcription 2, 113kDa	0.008	-1.2	√			√							√	
32043_at	8614	*STC2*	chr5q35.1	stanniocalcin 2	0.037	-1.3												
207_at	10963	*STIP1*	chr11q13	stress-induced-phosphoprotein 1	0.004	1.2												
38685_at	23673	*STX12*	chr1p35.3	syntaxin 12	0.032	1.4												
39523_at	8675	*STX16*	chr20q13.32	syntaxin 16	0.024	-1.2												
33079_at	10228	*STX6*	chr1q25.3	syntaxin 6	0.039	-1.2												
31804_f_at	6817	*SULT1A1*	chr16p12.1	sulfotransferase family, cytosolic, 1A, phenol-preferring, member 1	0.046	1.9												
38738_at	6612	*SUMO3*	chr21q22.3	SMT3 suppressor of mif two 3 homolog 3 (S. cerevisiae)	0.009	1.4											√	
36931_at	6876	*TAGLN*	chr11q23.2	transgelin	0.022	-1.3												
38473_at	6897	*TARS*	chr5p13.2	threonyl-tRNA synthetase	0.023	1.6												
32507_at	23216	***TBC1D1***	chr4p14	TBC1 (tre-2/USP6, BUB2, cdc16) domain family, member 1	0.042	-1.3												
38223_at	11138	*TBC1D8*	chr2q11.2	TBC1 domain family, member 8 (with GRAM domain)	0.011	1.8												
39398_s_at	6904	*TBCD*	chr17q25.3	tubulin folding cofactor D	0.006	1.2												
33794_g_at	6942	*TCF20*	chr22q13.3|22q13.3	transcription factor 20 (AR1)	0.014	-1.2												
32256_r_at	7013	*TERF1*	chr8q13	telomeric repeat binding factor (NIMA-interacting) 1	0.045	1.2												
40767_at	7035	*TFPI*	chr2q32	tissue factor pathway inhibitor (lipoprotein-associated coagulation inhibitor)	0.019	-1.1												
971_s_at	7042	*TGFB2*	chr1q41	transforming growth factor, beta 2	0.027	-1.3			√	√	√							
1767_s_at	7043	*TGFB3*	chr14q24	transforming growth factor, beta 3	0.007	-1.2				√								
32868_at	7053	*TGM3*	chr20q11.2	transglutaminase 3 (E polypeptide, protein-glutamine-gamma-glutamyltransferase)	0.015	-1.4												
33001_s_at	9333	*TGM5*	chr15q15.2	transglutaminase 5	0.032	-1.2												
31827_s_at	10618	*TGOLN2*	chr2p11.2	trans-golgi network protein 2	0.029	1.2		√										
32124_at	57187	***THOC2***	chrXq25-q26.3	THO complex 2	0.029	1.2												
1428_at	7068	*THRB*	chr3p24.2	thyroid hormone receptor, beta (erythroblastic leukemia viral (v-erb-a) oncogene homolo	0.016	-1.3												
35230_at	148022	*TICAM1*	chr19p13.3	toll-like receptor adaptor molecule 1	0.037	1.3					√			√		√	√	
41626_at	8914	*TIMELESS*	chr12q12-q13	timeless homolog (Drosophila)	0.034	1.2												
32830_g_at	10440	*TIMM17A*	chr1q32.1	translocase of inner mitochondrial membrane 17 homolog A (yeast)	0.026	1.4		√										
1693_s_at	7076	*TIMP1*	chrXp11.3-p11.23	TIMP metallopeptidase inhibitor 1	0.024	1.5												
40837_at	7089	*TLE2*	chr19p13.3	transducin-like enhancer of split 2 (E(sp1) homolog, Drosophila)	0.031	-1.2												
31333_at	7092	*TLL1*	chr4q32-q33	tolloid-like 1	0.016	-1.2												
36950_at	54732	*TMED9*	chr5q35.3	transmembrane emp24 protein transport domain containing 9	0.005	1.2		√										
37934_at	161291	***TMEM30B***	chr14q23.1	transmembrane protein 30B	0.039	-1.2												
40437_at	25963	*TMEM87A*	chr15q15.1	transmembrane protein 87A	0.013	1.6												
32682_at	7112	*TMPO*	chr12q22	thymopoietin	0.023	-1.1												
34067_at	164656	*TMPRSS6*	chr22q12.3	transmembrane protease, serine 6	0.038	-1.3												
595_at	7128	***TNFAIP3***	chr6q23	tumor necrosis factor, alpha-induced protein 3	0.048	1.5	√										√	
31410_at	23495	*TNFRSF13B*	chr17p11.2	tumor necrosis factor receptor superfamily, member 13B	0.025	-1.2				√	√					√		
35287_at	8741	*TNFSF13*	chr17p13.1	TNFSF12-TNFSF13 readthrough /// tumor necrosis factor (ligand) superfamily, member 13	0.002	1.6					√							
41748_at	7125	*TNNC2*	chr20q12-q13.11	troponin C type 2 (fast)	0.038	-1.4												
38896_at	30000	*TNPO2*	chr19p13.2	transportin 2	0.031	-1.4												
31377_r_at	23534	*TNPO3*	chr7q32.2	transportin 3	0.010	-1.2												
34598_at	7143	*TNR*	chr1q24	tenascin R (restrictin, janusin)	0.003	-1.1			√									
32360_s_at	8940	*TOP3B*	chr22q11.22	topoisomerase (DNA) III beta	0.045	-1.3												
1974_s_at	7157	*TP53*	chr17p13.1	tumor protein p53	0.036	-1.2	√				√				√		√	
32824_at	1200	*TPP1*	chr11p15	tripeptidyl peptidase I	0.022	1.4												√
33784_at	7186	*TRAF2*	chr9q34	TNF receptor-associated factor 2	0.036	-1.1	√				√				√		√	
36139_at	10758	*TRAF3IP2*	chr6q21	TRAF3 interacting protein 2	0.006	-1.2										√		
1468_at	10131	*TRAP1*	chr16p13.3	TNF receptor-associated protein 1	0.029	1.2	√										√	
36531_r_at	79090	***TRAPPC6A***	chr19q13.32	trafficking protein particle complex 6A	0.034	-1.3												
31322_at	6964	***TRD@***	chr14q11.2	T cell receptor delta locus	0.046	-1.1												
32955_at	51499	*TRIAP1*	chr12q24.31	TP53 regulated inhibitor of apoptosis 1	0.036	1.3	√											
36825_at	10346	***TRIM22***	chr11p15	tripartite motif-containing 22	0.033	2.4												
39412_at	7726	*TRIM26*	chr6p21.3	tripartite motif-containing 26	0.013	1.3												
34700_at	10612	*TRIM3*	chr11p15.5	tripartite motif-containing 3	0.040	-1.2												
41611_at	9325	*TRIP4*	chr15q22.31	thyroid hormone receptor interactor 4	0.005	1.3												
36806_at	55503	*TRPV6*	chr7q34	transient receptor potential cation channel, subfamily V, member 6	0.011	-1.2												
38469_at	7103	*TSPAN8*	chr12q14.1-q21.1	tetraspanin 8	0.038	-1.1												
36123_at	7263	***TST***	chr22q13.1	thiosulfate sulfurtransferase (rhodanese)	0.023	-1.3												
31899_at	9694	*TTC35*	chr8q23.1	tetratricopeptide repeat domain 35	0.009	1.3												
39332_at	347733	*TUBB2B*	chr6p25	tubulin, beta 2B	0.035	-1.1												
40985_g_at	27229	*TUBGCP4*	chr15q15	tubulin, gamma complex associated protein 4	0.042	-1.2												
36992_at	7295	*TXN*	chr9q31	thioredoxin	0.015	1.4	√											
38089_at	9898	*UBAP2L*	chr1q21.3	ubiquitin associated protein 2-like	0.004	1.4												
1725_s_at	7337	*UBE3A*	chr15q11.2	ubiquitin protein ligase E3A	0.040	1.5	√											
35161_at	22888	*UBOX5*	chr20p13	U-box domain containing 5	0.011	-1.2												
34422_r_at	7352	*UCP3*	chr11q13	uncoupling protein 3 (mitochondrial, proton carrier)	0.035	-1.4												
811_at	7353	*UFD1L*	chr22q11.21	ubiquitin fusion degradation 1 like (yeast)	0.009	1.2	√											
32392_s_at	54658	*UGT1A1*	chr2q37	UDP glucuronosyltransferase 1 family, polypeptide A1	0.025	-1.2												
32382_at	7348	*UPK1B*	chr3q13.32	uroplakin 1B	0.030	-1.2												
32448_at	7379	*UPK2*	chr11q23	uroplakin 2	0.036	-1.2												
283_at	7384	*UQCRC1*	chr3p21.3	ubiquinol-cytochrome c reductase core protein I	0.024	1.4												
38561_at	27340	*UTP20*	chr12q23	UTP20, small subunit (SSU) processome component, homolog (yeast)	0.032	-1.3												
33780_at	6843	*VAMP1*	chr12p	vesicle-associated membrane protein 1 (synaptobrevin 1)	0.048	-1.2												
41757_at	9217	*VAPB*	chr20q13.33	VAMP (vesicle-associated membrane protein)-associated protein B and C	0.039	1.3												
1704_at	7410	*VAV2*	chr9q34.1	vav 2 guanine nucleotide exchange factor	0.045	-1.2				√		√			√	√		
38111_at	1462	*VCAN*	chr5q14.3	versican	0.010	2			√									
33818_at	7415	*VCP*	chr9p13.3	valosin-containing protein	0.016	1.2												
31607_at	7416	*VDAC1*	chr5q31	voltage-dependent anion channel 1	0.031	-1.4												
37697_s_at	7417	*VDAC2*	chr10q22	voltage-dependent anion channel 2	0.046	1.4	√											

36102_at	7419	*VDAC3*	chr8p11.2	voltage-dependent anion channel 3	0.040	1.3												
334_s_at	7432	*VIP*	chr6q25	vasoactive intestinal peptide	0.041	-1.3												
40001_r_at	23230	*VPS13A*	chr9q21	vacuolar protein sorting 13 homolog A (S. cerevisiae)	0.027	1.2												
35779_at	11311	*VPS45*	chr1q21.2	vacuolar protein sorting 45 homolog (S. cerevisiae)	0.024	1.4												
34281_at	7447	*VSNL1*	chr2p24.3	visinin-like 1	0.017	-1.1												
38987_at	79084	*WDR77*	chr1p13.2	WD repeat domain 77	0.012	-1.2												
35164_at	7466	*WFS1*	chr4p16	Wolfram syndrome 1 (wolframin)	0.034	-1.3	√											
33392_at	26100	*WIPI2*	chr7p22.1	WD repeat domain, phosphoinositide interacting 2	0.018	1.3												
32359_at	8840	*WISP1*	chr8q24.22	WNT1 inducible signaling pathway protein 1	0.011	-1.2			√									
32358_at	8838	*WISP3*	chr6q21	WNT1 inducible signaling pathway protein 3	0.005	-1.3												
33718_at	58525	*WIZ*	chr19p13.1	widely interspaced zinc finger motifs	0.033	-1.2												
35305_at	7511	*XPNPEP1*	chr10q25.3	X-prolyl aminopeptidase (aminopeptidase P) 1, soluble	0.036	1.2												
40824_at	23039	*XPO7*	chr8p21	exportin 7	0.021	1.5												
38977_at	8565	*YARS*	chr1p35.1	tyrosyl-tRNA synthetase	0.009	1.4												
31922_i_at	9189	*ZBED1*	chrXp22.33 and Yp11	zinc finger, BED-type containing 1	0.017	-1.2												
38532_at	9880	*ZBTB39*	chr12q13.3	zinc finger and BTB domain containing 39	0.009	-1.1												
32587_at	678	*ZFP36L2*	chr2p22.3-p21	zinc finger protein 36, C3H type-like 2	0.032	1.6												
35611_at	7539	*ZFP37*	chr9q32	zinc finger protein 37 homolog (mouse)	0.050	-1.2												
37943_at	23503	*ZFYVE26*	chr14q24.1	zinc finger, FYVE domain containing 26	0.018	1.4												
34611_at	7745	*ZKSCAN8*	chr6p21.3	zinc finger with KRAB and SCAN domains 8	0.006	-1.1												
37116_at	23613	*ZMYND8*	chr20q13.12	zinc finger, MYND-type containing 8	0.007	-1.1												
36392_at	7694	***ZNF135***	chr19q13.4	zinc finger protein 135	0.018	-1.2												
41393_at	7748	*ZNF195*	chr11p15.5	zinc finger protein 195	0.003	1.3												
35838_at	57862	*ZNF410*	chr14q24.3	zinc finger protein 410	0.022	1.3												

Supplementary Table 1. CCLE DEGs (blood) associated with enriched, disease-related pathways and processes. We delineated an extended 783 DEGs from a comparison of peripheral blood of 3 CCLE patients vs. 3 healthy control individuals (fold change (FC) ≥ ±1.1 and p-value at <0.05). Eighty seven DEGs (in **bold**) are shared between the blood and our previously published skin expression profile (PMID: 24956118). Six of the 87 shared DEGs (**bold and underlined**) have corresponding genes in both lists which are either synonymous or have similar functions. Disease-related pathways and processes that are associated with DEGs in both blood and skin profiles are marked with √.
Abbreviations used : **FC,** Fold change**, CCLE**, chronic cutaneous lupus erythematosus.
Disease related pathways and processes: **Apop**; apoptosis, **Oxid:Red**; oxidation:reduction, **Cell adh**; cell adhesion, **Cyto-chemo**; cytokine/chemokine, **Leu che**; leukocyte chemotaxis, **NK**; NK cells, **TLR**; Toll like receptor signaling, **DC**; Decdritic cells, **TC**; T cell, **BC**; B cell, **IFN**; Type I interferon and **Lys/Prot**; lysosome/ proteasome related.

Running title: Interactome analysis: Cutaneous lupus- **Dey-Rao and Sinha, 2018**
