# Supplementary material for: In silico Analyses of Skin and Peripheral Blood Transcriptional Data in Cutaneous Lupus Reveals CCR2-A Novel Potential Therapeutic Target
Source: Front Immunol. 2019 Mar 29;10:640. doi: 10.3389/fimmu.2019.00640 (PMC6450170; doi:10.3389/fimmu.2019.00640)
Supplement: Supplementary file 2 [file Data_Sheet_2.PDF]

#	Gene Symbol	Entrez Gene	Chromosomal Location	Fold-Change (Lesional vs. nonlesional Skin)	p-value	Fold-Change (LUPUS vs. CONTROL-Blood)	p-value
1	***ACAA1***	30	chr3p23-p22	-1.4	0.045	1.4	0.030
2	***AHCYL1***	10768	chr1p13.2	-1.5	0.040	1.6	0.050
3	*AHNAK2*	113146	chr14q32.33	-2.0	0.016	-1.2	0.004
4	***AIM2***	9447	chr1q22	2.4	0.000	-1.1	0.029
5	*ANP32E*	81611	chr1q21.2	1.5	0.017	1.5	0.023
6	***AP3S2***	10239	chr15q26.1	-1.2	0.020	1.2	0.035
7	*APOA1*	335	chr11q23-q24	-1.3	0.009	-1.3	0.047
8	*APOBEC3G*	60489	chr22q13.1-q13.2	1.8	0.022	2.4	0.018
9	*ARL4C*	10123	chr2q37.1	1.8	0.022	1.7	0.030
10	*BICD1*	636	chr12p11.2-p11.1	1.1	0.027	1.1	0.023
11	*CALCOCO1*	57658	chr12q13.13	-1.3	0.034	-1.2	0.010
12	*CAP2*	10486	chr6p22.3	-1.2	0.011	-1.3	0.013
13	*CASP10*	843	chr2q33-q34	1.6	0.001	1.6	0.013
14	*CCR2*	729230	chr3p21.31	1.8	0.031	1.6	0.006
15	*CD163*	9332	chr12p13.3	2.0	0.015	2.0	0.000
16	***CD1A;CD1D***	912;909	chr1q22-q23	-1.6	0.026	1.6	0.002
17	*CD3G*	917	chr11q23	1.7	0.021	1.5	0.050
18	*CD48*	962	chr1q21.3-q22	3.2	0.011	1.5	0.045
19	*CRTAM*	56253	chr11q24.1	1.1	0.040	1.3	0.049
20	*CTSL*	1514	chr9q21.33	2.4	0.025	2.6	0.002
21	***CYB5A***	1528	chr18q23	-3.1	0.037	1.2	0.019
22	*DYNLT1*	6993	chr6q25.2-q25.3	1.7	0.013	1.7	0.031
23	***ECH1***	1891	chr19q13.1	-2.1	0.029	1.5	0.022
24	*EFNA1*	1942	chr1q21-q22	-1.5	0.016	-1.2	0.022
25	*ERBB3*	2065	chr12q13	-2.3	0.004	-1.2	0.041
26	*EXPH5*	23086	chr11q22.3	-1.5	0.033	-1.2	0.049
27	*FEN1*	2237	chr11q12	1.4	0.046	1.4	0.032
28	*FEZ2*	9637	chr2p21	-1.1	0.029	-1.2	0.006
29	FGFR2	2263	chr10q26	-2.0	0.016	-1.3	0.049
30	*FZD2*	2535	chr17q21.1	1.4	0.041	1.1	0.041
31	*GARS*	2617	chr7p15	1.5	0.034	1.7	0.002
32	*GAS1*	2619	chr9q21.3-q22	-2.3	0.005	-1.1	0.031
33	*GK*	2710	chrXp21.3	-1.4	0.043	-1.3	0.021
34	*GLS2;GLS*	2744	Chr2q32-q34	-1.3	0.035	-1.1	0.031
35	***GNLY***	10578	chr2p11.2	2.5	0.017	-2.7	0.010
36	*HDAC1*	3065	chr1p34	1.2	0.007	1.2	0.045

37	*IFI30*	10437	chr19p13.1	3.9	0.001	2.0	0.027
38	*IL10RA*	3587	chr11q23	3.2	0.000	1.3	0.047
39	*ITGB2*	3689	chr21q22.3	2.0	0.017	1.6	0.049
40	***KLRC1 /// KLRC2***	3821 /// 3822	chr12p13	1.9	0.035	-2.3	0.017
41	***KLRC1 /// KLRC2 /// KLRC3***	3821 /// 3822 /// 3823	chr12p13	2.6	0.018	-2.8	0.026
42	*L3MBTL1*	26013	chr20q13.12	-1.2	0.009	-1.2	0.047
43	*LAMA5*	3911	chr20q13.2-q13.3	-1.4	0.001	-1.3	0.030
44	*LGALS2*	3957	chr22q12-q13|22q13.1	2.5	0.004	2.0	0.012
45	*LGALS3BP*	3959	chr17q25	2.0	0.020	2.8	0.002
46	*LGALS9*	3965	chr17q11.2	1.9	0.002	1.5	0.037
47	*LHFPL2*	10184	chr5q14.1	1.4	0.038	1.8	0.010
48	*LILRB4*	11006	chr19q13.4	3.4	0.000	2.1	0.019
49	*LPCAT3*	10162	chr12p13	-1.7	0.029	-1.1	0.023
50	*LSM1*	27257	chr8p11.2	1.1	0.042	1.3	0.020
51	*MAOB*	4129	chrXp11.23	-1.2	0.041	-1.3	0.043
52	*MUC1*	4582	chr1q21	-2.5	0.006	-1.2	0.020
53	*TRAC;NCOR2*	28755;9612	chr14q11;chr12q24	3.2	0.005	1.8	0.015
54	*NDC80*	10403	chr18p11.32	2.0	0.014	1.7	0.019
55	***NNMT***	4837	chr11q23.1	1.9	0.035	-1.1	0.024
56	*OAS1*	4938	chr12q24.1	2.6	0.000	6.0	0.001
57	*OAS2*	4939	chr12q24.2	2.6	0.007	1.9	0.007
58	*ODC1*	4953	chr2p25	1.5	0.028	1.5	0.014
59	*PC;PODXL*	5091;5420	chr7q32-q33	-1.3	0.042	-1.3	0.021
60	***PLAU***	5328	chr10q24	1.6	0.017	-1.3	0.007
61	***PLIN2***	123	chr9p22.1	-3.8	0.001	1.5	0.044
62	*PLSCR1*	5359	chr3q23	2.4	0.010	2.6	0.042
63	*PSMB8*	5696	chr6p21.3	1.7	0.033	1.4	0.019
64	***RAMP3***	10268	chr7p13-p12	1.6	0.019	-1.2	0.012
65	***RELA***	5970	chr11q13	1.4	0.029	-1.2	0.008
66	*RGL1*	23179	chr1q25.3	1.9	0.031	1.4	0.048
67	***RNASE4***	6038	chr14q11.1	-1.9	0.014	1.4	0.045
68	*RNASE6*	6039	chr14q11.2	2.5	0.017	1.7	0.003
69	***RPS6KA2***	6196	chr6q27	1.3	0.027	-1.2	0.041
70	*SEMA5A*	9037	chr5p15.2	-1.5	0.007	-1.2	0.047
71	*SERPINB1*	1992	chr6p25	2.4	0.022	1.5	0.035
72	***SLC12A3***	6559	chr16q13	1.2	0.048	-1.3	0.030
73	*SLC15A1*	6564	chr13q33-q34	-1.8	0.048	-1.2	0.036
74	*SLC26A3*	1811	chr7q31	-1.2	0.029	-1.1	0.026
75	*SLC7A7*	9056	chr14q11.2	1.5	0.006	1.5	0.036

76	*STAT1*	6772	chr2q32.2	4.2	0.003	2.2	0.043
77	***TAP1;SEC14L2***	23541;6890	Chr22q12.2;chr6p21.3	2.1	0.016	-1.3	0.03
78	***TBC1D1***	23216	chr4p14	1.3	0.011	-1.3	0.042
79	***TF;F3***	7018	chr3q22.1	-1.9	0.007	1.1	0.04
80	*THOC2*	57187	chrXq25-q26.3	1.4	0.001	1.2	0.029
81	*TMEM30B*	161291	chr14q23.1	-1.1	0.037	-1.2	0.039
82	*TNFAIP3*	7128	chr6q23	2.1	0.003	1.5	0.048
83	*TRAPPC6A*	79090	chr19q13.32	-1.4	0.000	-1.3	0.034
84	***TRD@***	6964	chr14q11.2	1.4	0.013	-1.1	0.046
85	*TRIM22*	10346	chr11p15	2.8	0.020	2.4	0.033
86	*TST*	7263	chr22q13.1	-1.8	0.048	-1.3	0.023
87	*ZNF135*	7694	chr19q13.4	-1.3	0.048	-1.2	0.018

Supplementary Table 2. Overlap in CCLE lesional skin and blood transcriptional profiles. Using similar criteria for DEGs inclusion, we initially generated a comparable list of 783 DEGs in CCLE blood (CCLE patients vs. controls) as our previously published 776 DEGs from lesional skin (lesional vs. non-lesional). We found 87 DEGs common (a few with similar functions) between the transcriptional profiles from the two tissue environments. Of these, 49 transcripts were up- and 38 were down-regulated in CCLE blood and 53 transcripts were up- and 34 were down-regulated in CCLE lesional skin transcriptional profile. Among the overlapping transcripts, twenty-two genes (**in bold**) were dysregulated in the opposite directions in the CCLE- blood and skin analyses.

Running title: Interactome analysis: Cutaneous lupus- **Dey-Rao and Sinha, 2018**
