# Supplementary material for: In silico Analyses of Skin and Peripheral Blood Transcriptional Data in Cutaneous Lupus Reveals CCR2-A Novel Potential Therapeutic Target
Source: Front Immunol. 2019 Mar 29;10:640. doi: 10.3389/fimmu.2019.00640 (PMC6450170; doi:10.3389/fimmu.2019.00640)
Supplement: Supplementary file 3 [file Data_Sheet_3.PDF]

Chromosome Region	#genes	GENE SYMBOL
1q21- q25.1	19	*S100A7, SHC1, GJA5, ANP32E, VPS45, ENSA, C1orf77, INTS3, UBAP2L, **CD48**, EFNA1, AIM2, CD1D, CCT3, LOC100131825, BLZF1, GPR161, FMO6P, TNR*
3p21.2-p22.3	10	*RARB, CRTAP, CX3CR1, GLB1, **CCR2**, MAP4, **RHOA**, UQCRC1, MAPKAPK3, HYAL1*
5q31.1-q35	17	***H2AFY**, GFRA3, PURA, CD14, PDE6A, GNPDA1, HMHB1, KIAA0141, HTR4, PPP2R2B, GLRA1, GM2A, SGCD, FGF18, GABRA1, RARS,STC2*
6p21.1-p23	12	*ATXN1, ACOT13, CAP2, HIST1H2AG, HIST1H2BM, **CSNK2B**, DHX16, RDBP, TRIM26, PSMB8, CDKN1A, C6orf1*
11q12-q13	6	*FEN1, DNAJC4, STIP1, GSTP1, PLCB3, ALDH3B1*
11q23.1-q24.3	13	*HTR3A, NNMT, TAGLN, PAFAH1B2, **IL10RA**, **CD3G**, UPK2, **APOA1**, EI24, CRTAM, CHEK1, BARX2, KCNJ5*
12q12-q15	20	*ADCY6, TIMELESS, CD63, PRKAG1, IGFBP6, AMHR2, KRT18, RAB5B, CALCOCO1, MYL6B, SMARCC2, MARS, ZBTB39, STAT2, GPR182, SMARCD1, AVIL, AGAP2, TSPAN8, PTPRB*
12q24.31	5	*RAB35, TRIAP1, MLEC, PXN, HNF1A*
14q11.2	7	*OSGEP, **RNASE4**, SLC7A7, RNASE6, **RNASE2**, RNASE3, LRP10*
15q15.1-q21.1	9	*NDUFAF1, **TMEM87A**, TGM5, TUBGCP4, MFAP1, SPG11, SLC28A2, **EIF3J**, FBN1*
16q22.1-q22.3	5	*SLC12A4, ACD, PSKH1, NFATC3, PSMD7*
19q13.1-q13.4	19	*SCN1B, ECH1, CLC, RYR1, PSMC4, GAPDHS, PSMD8, PLD3, DHX34, KLK1, GRLF1, KLK6, RTN2, PRRG2, MYBPC2, RRAS, LILRA1, LILRB4, KIR3DL1 /// KIR3DL2 /// LOC727787*
20p13	6	*SNPH, ProSAPiP1, UBOX5, SNRPB, PTPRA, TGM3*
21q22.2-q22.3	6	*SH3BGR, DSCAM, PKNOX1, PDXK, SUMO3, ITGB2*
22q11.21-q13.31	18	*HIRA, CDC45, ARVCF, UFD1L, TOP3B, C22orf28, **SEC14L2**, DEPDC5, HMOX1, **LGALS2**, SERHL /// SERHL2, ATF4, TST, GTPBP1, LGALS1, APOBEC3G, CELSR1, FBLN1*
Xq28	5	*DUSP9, CTAG2, DNASE1L1, LAGE3, DKC1*

Supplementary Table 3. Chromosomal locations of the 16 transcriptional "hot spots" across the CCLE blood signature. A total of 177 CCLE blood DEGs are included in 16 "hot spots". Six DEGs (underlined) are associated with previously putative susceptibility loci in SLE. Thirteen CCLE blood DEGs (**bold**) from the present analysis are also previously reported as SLE associated DEGs. *CSNK2B* (**bold** and underlined) has been reported as a CLE-susceptibility locus.

Running title: Interactome analysis: Cutaneous lupus- **Dey-Rao and Sinha, 2018**
