# Supplementary material for: In silico Analyses of Skin and Peripheral Blood Transcriptional Data in Cutaneous Lupus Reveals CCR2-A Novel Potential Therapeutic Target
Source: Front Immunol. 2019 Mar 29;10:640. doi: 10.3389/fimmu.2019.00640 (PMC6450170; doi:10.3389/fimmu.2019.00640)
Supplement: Supplementary file 4 [file Data_Sheet_4.PDF]

Protein class	Actual	n	R	N	E	Ratio	p-value	z-score	In data set %	In protein function %	Protein function in database %
**Ligands**	78	1665	523	38444	22.7	3.44	1.46E-21	12.0	4.7	14.9	1.4
**Proteases**	79	1665	583	38444	25.3	3.13	2.98E-19	11.0	4.7	13.6	1.5
**Receptors**	210	1665	1674	38444	72.5	2.90	8.94E-45	16.9	12.6	12.5	4.4
**Kinases**	79	1665	657	38444	28.5	2.78	3.2E-16	9.8	4.7	12.0	1.7
**Phosphatases**	28	1665	239	38444	10.4	2.71	1.96E-06	5.6	1.7	11.7	0.6
**Enzymes**	286	1665	2798	38444	121.2	2.36	1.44E-43	15.9	17.2	10.2	7.3
**Transcription factors**	119	1665	1212	38444	52.5	2.27	8.44E-17	9.5	7.2	9.8	3.2
Other	803	1665	30812	38444	1334.0	0.60	0	-33.4	48.2	2.6	80.2

Supplementary Table 4. Enrichment by Protein Function. CCLE- peripheral blood and skin DEGs lists were activated and corresponding network objects as well as nodes were mapped in MetaCore. We then conducted an enrichment analysis by protein function that scores and ranks the most relevant protein functions related to the activated dataset. Protein class (**in bold**) are the significant functional categories.

Explanation of each column: Protein class: a broadly defined protein function; Actual; number of network objects from the activated dataset(s) for a given protein class; n: total number of network objects in the activated dataset(s); R: total number of network objects of a given protein class in the complete database or background list; N: total number of network objects in the complete database or background list; E: # of objects that would be expected to occur by chance, mean value for hypergeometric distribution (n*R/N); Ratio: connectivity ratio (Actual/Expected); z-score: {(Actual-Expected)/sqrt (variance)}; p-value: probability to have the given value of Actual or higher (or lower for negative z-score); In data set %: fraction of network objects with a selected function in the activated dataset; In protein function %: fraction of network with a selected function in the activated dataset among network objects with this function in the complete database or background list; protein function in database %: fraction of network objects with a selected function in the complete database or background list.

Running title: Interactome analysis: Cutaneous lupus- **Dey-Rao and Sinha, 2018**
