# Supplementary material for: In silico Analyses of Skin and Peripheral Blood Transcriptional Data in Cutaneous Lupus Reveals CCR2-A Novel Potential Therapeutic Target
Source: Front Immunol. 2019 Mar 29;10:640. doi: 10.3389/fimmu.2019.00640 (PMC6450170; doi:10.3389/fimmu.2019.00640)
Supplement: Supplementary file 5 [file Data_Sheet_5.PDF]

	Complete database	Experiment(s)	Experiment(s) only
Degree	17.17	62.68	10.86
Degree-in	8.585	30.89	5.43
Degree-out	8.585	31.79	5.43
Clustering coefficient	0.05093	0.0938	0.1069

Supplementary Table 5. Interactome topology and over-connected genes. We annotated the CCLE-blood and skin experimental datasets and subjected them to interactome analysis: **Interactome topology:** The experimental CCLE- datasets show connectivity (coming in and going out) to objects within the CCLE-datasets (0.1069) and to objects in the metabase (0.0938) both with a higher clustering coefficient than connectivity only among objects within the larger metabase (0.05093).

Explanation of each column and row:
Degree: average number of interactions per node; Degree in: average number of ingoing interactions per node; Degree-out: average number of outgoing interactions per node; Clustering coefficient: average clustering coefficient for node; Complete database: calculated for the complete database or background list; Experiment(s): calculated for the complete database; average is calculated for the experimental set; Experiment(s) only: calculated for the experimental set (zero values in this column means that there is no interactions between objects from experimental set.

Running title: Interactome analysis: CCLE skin and blood profiles- **Dey-Rao and Sinha, 2018**
