# Supplementary material for: In silico Analyses of Skin and Peripheral Blood Transcriptional Data in Cutaneous Lupus Reveals CCR2-A Novel Potential Therapeutic Target
Source: Front Immunol. 2019 Mar 29;10:640. doi: 10.3389/fimmu.2019.00640 (PMC6450170; doi:10.3389/fimmu.2019.00640)
Supplement: Supplementary file 6 [file Data_Sheet_6.PDF]

Protein Function	Gene IDs in active data set	Object name Metabase	Actual	n	R	N	Expected	Ratio	z-score	p-value
**Transcription factors**	*STAT1*	STAT1	146	1665	610	38444	26.42	5.53	23.98	2.7585E-66
	RELA	NF-kB p65/p65	16	1665	90	38444	3.90	4.10	6.27	1.4758E-06
	TRIM22	Staf-50	4	1665	21	38444	0.91	4.40	3.31	0.01162539
**Receptors**	***ITGB2***	ITGB2	22	1665	113	38444	4.89	4.50	7.92	2.939E-09
	CCR2	CCR2	14	1665	49	38444	2.12	6.60	8.34	1.2552E-08
	ERBB3	ErbB3	21	1665	129	38444	5.59	3.76	6.68	1.7148E-07
	FGFR2	FGFR2	20	1665	127	38444	5.50	3.64	6.33	5.792E-07
	LGALS3BP	90K	14	1665	86	38444	3.72	3.76	5.45	1.8848E-05
	LILRB4	ILT3	4	1665	8	38444	0.35	11.54	6.35	0.00021329
	RAMP3	RAMP3	5	1665	16	38444	0.69	7.22	5.29	0.00044317
**Ligands**	*APOA1*	APOA1	29	1665	143	38444	6.19	4.68	9.39	3.2738E-12
	EFNA1	Ephrin-A1	7	1665	33	38444	1.43	4.90	4.77	0.00044726
**Kinases**	*ERBB3**	ErbB3	21	1665	129	38444	5.59	3.76	6.68	1.7148E-07
	FGFR2*	FGFR2	20	1665	127	38444	5.50	3.64	6.33	5.792E-07
	GK	GLPK	7	1665	28	38444	1.21	5.77	5.37	0.00014982
	RPS6KA2	p90RSK3(RPS6KA2)	5	1665	35	38444	1.52	3.30	2.89	0.01668052
**Proteases**	*PLAU*	PLAU (UPA)	25	1665	126	38444	5.46	4.58	8.57	1.6423E-10
	PSMB8	PSMB8(LMP7)	12	1665	30	38444	1.30	9.24	9.60	1.7516E-09
	CTSL	Cathepsin V	9	1665	29	38444	1.26	7.17	7.07	2.3918E-06
	CASP10	Caspase-10	11	1665	65	38444	2.82	3.91	4.99	0.00010095
**Enzymes**	*HDAC1*	HDAC1	145	1665	1068	38444	46.25	3.13	15.05	1.2667E-34
	FEN1	FEN1	16	1665	73	38444	3.16	5.06	7.39	7.2555E-08
	OAS1	OAS1	10	1665	32	38444	1.39	7.22	7.48	6.0821E-07
	OAS2	OAS2	5	1665	12	38444	0.52	9.62	6.35	9.2917E-05
	ECH1	ECH1	7	1665	30	38444	1.30	5.39	5.12	0.00023878
	CYB5A	Cytochrome B5	5	1665	24	38444	1.04	4.81	3.97	0.00323186
	GARS	GlyRS	5	1665	29	38444	1.26	3.98	3.42	0.00755104
	RNASE4	RNASE4	3	1665	10	38444	0.43	6.93	3.99	0.0077386
	AHCYL1	SAHH2	6	1665	44	38444	1.91	3.15	3.03	0.0112818
	LGALS2	Galectin-3	54	1665	216	38444	9.35	5.77	14.97	3.978E-26
	MUC1	MUC1	33	1665	116	38444	5.02	6.57	12.78	2.2563E-18

	CD48	CD48	11	1665	19	38444	0.82	13.37	11.47	5.3383E-11
	TNFAIP3	A20	22	1665	128	38444	5.54	3.97	7.16	3.2165E-08
	LGALS9	Galectin-9	9	1665	30	38444	1.30	6.93	6.91	3.2855E-06
	PLIN2	Adipophilin	9	1665	35	38444	1.52	5.94	6.22	1.3331E-05
	PLSCR1	PL scramblase 1	21	1665	172	38444	7.45	2.82	5.09	1.92E-05
	CAP2	CAP2	7	1665	23	38444	1.00	7.03	6.15	3.7516E-05
**Others**	***L3MBTL1***	L3MBTL	8	1665	35	38444	1.52	5.28	5.39	0.00010053
	CD3G	CD3 gamma	5	1665	13	38444	0.56	8.88	6.05	0.0001456
	FEZ2	FEZ2	10	1665	66	38444	2.86	3.50	4.32	0.00052156
	AIM2	AIM2	6	1665	25	38444	1.08	5.54	4.83	0.00056917
	RGL1	RGL1	5	1665	23	38444	1.00	5.02	4.10	0.00265208
	SERPINB1	SERPINB1	5	1665	23	38444	1.00	5.02	4.10	0.00265208
	NDC80	HEC	11	1665	96	38444	4.16	2.65	3.43	0.00293925
	BICD1	BICD1	5	1665	24	38444	1.04	4.81	3.97	0.00323186
	SLC15A1	PEPT1	3	1665	12	38444	0.52	5.77	3.52	0.01329741

Supplementary Table 6. Interactome analysis of CCLE blood and skin DEGs: We first activated both the CCLE skin and blood DEG lists and investigated "interactions by protein function" based on their connectivity with proteins from the (background) total Human Proteome in the MetaCore database (metabase). We found several significantly over-connected proteins in our transcriptional profiles where the number of observed interactions exceeded the number of expected interactions. We then investigated only those over-connected genes which coincide with the 87 overlapping CCLE skin and blood profiles and have listed 44 genes that are assigned to 7 major functional categories that are significantly over-connected to network objects in our own data as well as network objects in the entire metabase. DEGs that do not overlap with previously reported SLE expressed genes or susceptibility loci are in **bold**. *ERBB3* and *FGFR2* are receptor tyrosine kinases that appear in both receptor and kinase groups. This helps us to prioritize potentially CCLE- relevant individual genes/proteins and hubs. We focus in on the 7 receptor genes/proteins (shaded in yellow).
Explanation of each column:
Protein Function: overall associated functions with proteins; Gene IDs in active data sets: gene symbol associated with the CCLE skin and blood signatures; Actual: number of network objects in the activated signatures which interact with the chosen object; n: number of network objects in the signature; R: number of network objects in the background list which interact with the chosen object; N: total number of protein-based objects in the background list; Expected: mean of hypergeometric distribution. Ratio: connectivity ratio (Actual/Expected); z-score: (Actual-Expected)/(standard deviation); p-value: probability to have the value of Actual or higher (lower for negative z-score) by chance under null hypothesis of no over- or under-connectivity.

Running title: Interactome analysis: Cutaneous lupus- **Dey-Rao and Sinha, 2018**
