# Supplementary material for: In silico Analyses of Skin and Peripheral Blood Transcriptional Data in Cutaneous Lupus Reveals CCR2-A Novel Potential Therapeutic Target
Source: Front Immunol. 2019 Mar 29;10:640. doi: 10.3389/fimmu.2019.00640 (PMC6450170; doi:10.3389/fimmu.2019.00640)
Supplement: Supplementary file 7 [file Data_Sheet_7.PDF]

Type	Sub-Type	Name of drug	Type	Clinical Trial	Clinical Trial #	Type/Route /Delivery	Pubmed	Recruitment Status	Details
Topical	Corticosteroid	Fluocinonide	Small molecule	Phase 0	NCT02176148	cream	28476075, 19821298	Recruiting	A topical glucocorticoid, synthetic steroids used topically as anti-inflammatory and antipruritic agents. Corticosteroids play a role in cellular signaling, immune function, inflammation, and protein regulation; however, the precise mechanism of action of fluocinonide cream in corticosteroid responsive dermatoses is unknown.
	Calcineurin inhibitor	Pimecrolimus (Elidel)	Small molecule	Unknown	NCT00222183	cutaneous/ cream	18797893	Recruiting status is unknown currently, completion date passed.	Topical calcineurin inhibitors: down-regulate T-cell activity by inhibiting the phosphatase calcineurin, responsible for dephosphorylation of the nuclear factor of activated T cells. It has a higher affinity to skin due to its lipophilic characteristic .
		Tacrolimus	Small molecule	Phase II	NCT00317681	ointment	14513247, 16547761, 16501922	Completed	Tacrolimus targets T lymphocytes and suppresses their activation by inhibiting the expression of cytokine genes, such as IL-2. Therefore, treatment of cutaneous lupus erythematosus with topical tacrolimus might result in an improvement of skin lesions in such patients.
	Anti-inflammatory	R-salbutamol sulphate (ASF-1096; Levabuterol)	Small molecule	Phase II	NCT00625157	cream	19681862	Completed	R-salbutamol binds to and activates B2 adrenoreceptors and inhibits CD4 cells and other leukocytes (monocytes, macrophages and langerhans cells). It thus inhibits activation of inflammatory genes, proinflammatory cytokines IL-2 and IFN-g. R -salbutamol also inhibits superoxide generation and peroxiase release.
	Glucocorticoid	Methylprednisolone Acetate	Small molecule	FDA app	(NDA) 011757	intralesional	14278445	Completed	Methylprednisolone acetate is a prodrug of methylprednisolone Because of methylprednisolone's low water solubility, a sodium acetate salt of methylprednisolone is used as an injectable dosage form.
	Corticosteroids (synthetic)	Triamcinolone diacetate	Small molecule	FDA app	(NDA) 084072	intralesional		Discontinued	Triamcinolone diacetate is a synthetic corticosteroid. It is a prodrug of triamcinolone. Aristocort /Kenacort discontinued
		Triamcinolone acetonide	Small molecule	FDA app	(NDA) 012041	intralesional/injectable	28342206	Discontinued	The acetonide salt form of triamcinolone, a synthetic glucocorticosteroid with immunosuppressive and anti-inflammatory activity. Triamcinolone acetonide
		Triamcinolone hexacetonide	Small molecule	FDA app	(NDA) 016466	intralesional/injectable	28343615	Completed	Triamcinolone injection is a steroid. It prevents the release of inflammatory substances.
	Anti- malarial	Hydroxychloroquine	mall molecu	FDA app	(NDA) 009768	oral/tablet	21768444, 90616669, 9733451	Completed	Chloroquine and hydroxychloroquine increase pH within intracellular vacuoles and alter processes such as protein degradation by acidic hydrolases in the lysosome, assembly of macromolecules in the endosomes, and posttranslation modification of proteins in the Golgi apparatus. Antimalarials also inhibit binding of antigens to the major histocompatibility complex. The inhibition of antigen processing also impairs the production of antibodies, the activity of natural killer cells, and the release of interleukin-1, interleukin-2, and tumor necrosis factor-alfa. Smoking reduces efficacy.

**Systemic**	Retinoids	Alitretinoin	Small molecule	Phase II	NCT01407679	oral/capsule	22890744	Terminated	9-cis-retinoic acid is an active retinoid that regulates expression of retinoid responsive genes, serving as ligand for two classes of ligand-dependent transcription factors, the retinoic acid receptors and retinoid X receptors. Their use is limited in young women due to teratogenicity. There is report of the vitamin-A derivative, alitretinoin in the treatement of CLE patients (PMID: 22890744)
	Thalidomide	Thalidomide	Small molecule	Phase II	NCT00001680	topical	27097914, 26872954	Completed	Thalidomide is known to inhibit synthesis of TNF-alfa and to modify the expression of TNF-alfa-induced adhesion molecules on endothelial cells and human leukocytes.Thalidomide inhibits inflammatory cytokine synthesis and prevents UVB –induced keratinocyte apoptosis.Neurotxic tetragenic side effects. Dose should be reduced to a minimum.
	Lenalidomide	Lenalidomide	Small molecule	Phase IV	NCT01408199, NCT00633945	oral	26873674, 24528907	Completed	A thalidomide analog with potential antineoplastic activity. Lenalidomide inhibits TNF-alpha production, stimulates T cells, reduces serum levels of the cytokines vascular endothelial growth factor (VEGF) and basic fibroblast growth factor (bFGF), and inhibits angiogenesis. This agent also promotes G1 cell cycle arrest and apoptosis of malignant cells.
	Methotrexate	Methotrexate	Small molecule	Phase III	NCT00470522	oral, IV or SC	16029342	Completed	MTX inhibits the enzyme dihydrofolate reductase and thus T-cell proliferation. MTX was first used for SLE but is now shown to be effective to a degree for DLE and SCLE. Significant reduction in autoantibodies. Side effects : nephro- and hepato- and bone marrow- toxicity. PMID:11907517. Low doses of MTX in carefully selected CLE patients (carefully monitored for side effecrts and contraindications) appears to be safe as shown by Wenzel et al., 2005. PMID 16029342
**Experimental Agents /Small**	Immuno-suppressants	Tanzisertib	Small molecule	Phase II	NCT01466725	oral/capsule	25475995	Terminated	An orally active anti-fibrotic JNK inhibitor.
		TRX-1	Biologic	Phase Ib	http://bit.ly/2s0ifrj		Tolerex Inc.		TRX1 is a novel humanized monoclonal antibody (MAb) that binds to the CD4 receptor found on both T effector cells and T regulatory cells. TRX1 is expected to block the activation and function of T effector cells and to favor dominance of T regulatory cells. This process, referred to as down regulation of the immune system, results in hyporesponsiveness, or tolerance, to antigens. In a preclinical study in a primate model, administration of TRX1 during the development of a primary immune response to a foreign protein, or antigen, resulted in longstanding and specific tolerance to that antigen. This type of suppression of the immune system may have therapeutic benefit in treating autoimmune diseases that occur when the human immune system mistakenly identifies the components of the human body as foreign.
		AMG557	Biologic	Phase I	NCT01389895	SC/ injection	23370250	Terminated	AMG-557 is a blocking anti-ICOSL antibody. AMG-557 prevent the interaction of B7RP-1 with ICOS on the surface of activated T cells. http://bit.ly/1BBiPz7
		AMG811	Biologic	Phase I	NCT01164917		28118537	Terminated	AMG 811 is a fully human monoclonal antibody that binds to interferon gamma, a widely active pro-inflammatory cytokine. It is being investigated as a treatment DLE.
		CC-11050	Small molecule	Phase II	NCT01300208	oral	26981575	Completed	CC-11050 is an oral, small-molecule, tumor necrosis factor (TNF)-alpha and PDE 4 inhibitor. There are safety concerns.

[illegible] molecules and biologics	[illegible]	R333	Small molecule	Phase II	NCT01597050	cutaneous /ointment	Rigel Pharmaceuticals, Inc.	Completed	R333 (R-932333) is a topical JAK/spleen tyrosine kinase (SYK) inhibitor.
		KRP-203	Small molecule	Phase II	NCT01294774	oral	not found	Terminated	Sphingosine-1-phosphate receptor (S1PR) agonist.
		PD0360324	Biologic	Phase I	NCT01470313	intravenous	not found	Terminated	Human mAb against macrophage colony stimulating factor (M-CSF). The decision to terminate the trial was not based on any clinical safety or efficacy concerns.
		Apremilast	Small molecule	Phase I/II	NCT00708916	oral/tablet	23134988	Completed	Apremilast is a well-tolerated, selective PDE4 (phosphodiesterase-4) inhibitor with a demonstrated inhibitory effect on inflammatory mediators and is under development for the treatment of inflammatory and immune mediated conditions.
		Efalizumab	Biologic	Phase II	NCT00308204	subcutaneous/ injection	28521707	Terminated	Immunosuppressive recombinant humanized IgG1 kappa isotype mAb that binds to human CD11a.
		Etanercept	Biologic	Phase II	NCT00797784	subcutaneous	28626974	Recruiting	It interfers with tumor necrosis factor (TNF; a soluble inflammatory cytokine) by acting as a TNF inhibitor. PMID: 19638570 PMID: 16134723, PMID: 16490847, PMID: 14969575
		Sirukumab	Biologic	Phase I	NCT01702740	intravenous/infusion	24525782	Completed	Sirukumab (CNTO 136) is a human anti-interleukin-6 (IL-6) monoclonal antibody

Drugs for the proposed novel CCLE/DLE relevant molecular target-*CCR2*

Type	Sub-Type	Name of drug	Type	Clinical Trial	Link to study	Disease	Pubmed/ PMID #	Recruitment Status	Target Molecule	Effect	Details
Suggested Novel Agents in present study	Xenobiotics	CCX915	Small molecule	Phase I	http://bit.ly/2uj2vEK	Multiple sclerosis, rheumatoid arthritis, diabetes mellitus and cardiovascular diseases.	http://ir.chemocentryx.com/releasedetail.cfm?ReleaseID=290843	Started	*CCR2*	Inhibition	CCX915 is a highly selective inhibitor of the CCR2 chemokine receptor which is implicated in the damaging inflammation underlying multiple sclerosis and other autoimmune and inflammatory diseases-Chemocentryx discovered CCX915 using the company's proprietary high throughput screening RAM Assay™
		CCX140-B	Small molecule	Phase II-Type 2 Diabetes Mellitus	NCT01028963		26268910	Completed	*CCR2*	Inhibition	CCX140 is a potent and selective small molecule antagonist of the CCR2 chemokine receptor.
		TAK-779	Small molecule	Preclinical			18270317, 16870431, 12954060, 12496074		*CCR2*	Inhibition	TAK-779 was discovered as an extremely potent antagonist of chemokine receptors CCR2 and CCR5 with a small molecular weight.
	Biologic	MLN1202	mAB	Phase II-Multiple sclerosis	NCT01199640		18576534	Completed	*CCR2*	Inhibition	Humanized monoclonal antibody that specifically targets the CCR2 chemokine receptor.

Supplementary Table 7. Existing emerging and experimental therapeutic agents in treatment of CCLE/DLE. Details of drugs and therapeutic agents involved in DLE treatment have been included in this table linked to some of the ongoing or completed clinical trials. The standard of care is topical application of corticosteroids and their derivatives on a case by case basis with a second line of treatment using systemic drugs that have been used in systemic lupus or other inflammatory diseases previously. Several drugs that are effectively used in other autoimmune and inflammatory diseases show severe side effects upon long-tern usage in patients with CCLE/DLE, and thus need to be used with care. Due to the lack of well-designed clinical trials, none of the drugs have passed all criteria for final approval. We have included some of the new therapeutic agents which make use of humanized monoclonal antibodies, biologics and small molecules that

[illegible] being tested in private clinics and linked the limited literature connected to these treatments. In the "Disease Road Map" (Figure 3), we have pinpointed some of the known targets of the treatments in CLE. We have used the known therapeutic approaches in CLE along with our strategy of combining genome-wide expression with chromosome mapping to discover and speculate about drugs that target the CCLE-related molecule *CCR2*. These drugs are being used in the clinic currently to treat diseases other than CCLE. We include in the suggested novel agents in present study section (shaded in grey). Drugs that are FDA approved for diseases other than CLE can be searched for by the (NDA) number in: http://www.accessdata.fda.gov/scripts/cder/drugsatfda/index.cfm . Clinical trials can be found linked to NCT# at https://clinicaltrials.gov Abbreviations : mAB = monoclonal antibody.

Running title: Interactome analysis: Cutaneous lupus- **Dey-Rao and Sinha, 2018**
