# Supplementary material for: In silico Analyses of Skin and Peripheral Blood Transcriptional Data in Cutaneous Lupus Reveals CCR2-A Novel Potential Therapeutic Target
Source: Front Immunol. 2019 Mar 29;10:640. doi: 10.3389/fimmu.2019.00640 (PMC6450170; doi:10.3389/fimmu.2019.00640)
Supplement: Supplementary file 8 [file Data_Sheet_8.PDF]

A

KEGG Pathway Term	Hsa#	Count	Fold Enrichment	p-value	Genes
Antigen processing and presentation	04612	6	7.8	8.32E-04	*KLRC2, KLRC3, TAP1, IFI30, CTSL, KLRC1*
Arginine and proline metabolism	00330	4	8.1	0.01	*GLS2, ODC1, GLS, MAOB*
Natural killer cell mediated cytotoxicity	04650	5	4.0	0.03	*CD48, KLRC2, KLRC3, ITGB2, KLRC1*
D-Glutamine and D-glutamate metabolism	00471	2	54.0	0.03	*GLS2, GLS*

B

Biological Process term	GO #	Count	Fold Enrichment	p-value	Genes
Cellular defense response	0006968	7	18.5	1.88E-06	*KLRC2, LGALS3BP, KLRC3, TRAC, RELA, GNLY, CCR2, TRD@*
Defense response	0006952	16	4.2	4.25E-06	*TF, KLRC2, KLRC3, RELA, GNLY, RNASE6, APOBEC3G, ITGB2, TRD@, CD163, CD1D, CD48, LGALS3BP, TRAC, F3, TAP1, CCR2*
Immune response	0006955	13	3.0	9.77E-04	*CRTAM, RELA, CD1A, APOBEC3G, OAS1, OAS2, TRIM22, PSMB8, AIM2, CD1D, TAP1, CCR2, LILRB4*
Response to virus	0009615	5	7.4	0.004	*PLSCR1, RELA, APOBEC3G, STAT1, TRIM22*
Cell morphogenesis	0000902	8	3.6	0.006	*SEMA5A, CAP2, CD3G, ERBB3, LAMA5, NDC80, GAS1, FEZ2*
Antigen processing and presentation	0019882	4	7.8	0.01	*IFI30, CD1A, PSMB8, CD1D*
Response to wounding	0009611	9	2.7	0.01	*TF, PLSCR1, ERBB3, RELA, F3, CCR2, ITGB2, PLAU, CD163*
Regulation of cell proliferation	0042127	11	2.3	0.02	*FGFR2, ODC1, HDAC1, ERBB3, LAMA5, RELA, F3, IFI30, GAS1, STAT1, PLAU*
Cell motion	0006928	8	2.7	0.02	*SEMA5A, APOA1, LAMA5, PODXL, ITGB2, GAS1, FEZ2, PLAU*
Response to organic cyclic substance	0014070	4	5.3	0.03	*TF, PLIN2, RELA, STAT1*

T cell activation	0042110	4	5.1	0.04	*CD48, CRTAM, CD3G, CD1D*
Inflammatory response	0006954	6	3.0	0.04	*TF, RELA, F3, CCR2, ITGB2, CD163*
Regulation of apoptosis	0042981	10	2.0	0.05	*CASP10, CRTAM, CD3G, HDAC1, ERBB3, RELA, F3, GAS1, TNFAIP3, STAT1*

Supplementary Table 8. Functional annotation and pathway analysis. The 87 overlapping CCLE blood and lesional skin DEGs were used in this analysis. The table summarizes enriched **(A)** KEGG pathways and **(B)** GO (Gene Ontology) biological processes.
