# Supplementary figures and images for: In silico Analyses of Skin and Peripheral Blood Transcriptional Data in Cutaneous Lupus Reveals CCR2-A Novel Potential Therapeutic Target
Source: Front Immunol. 2019 Mar 29;10:640. doi: 10.3389/fimmu.2019.00640 (PMC6450170; doi:10.3389/fimmu.2019.00640)

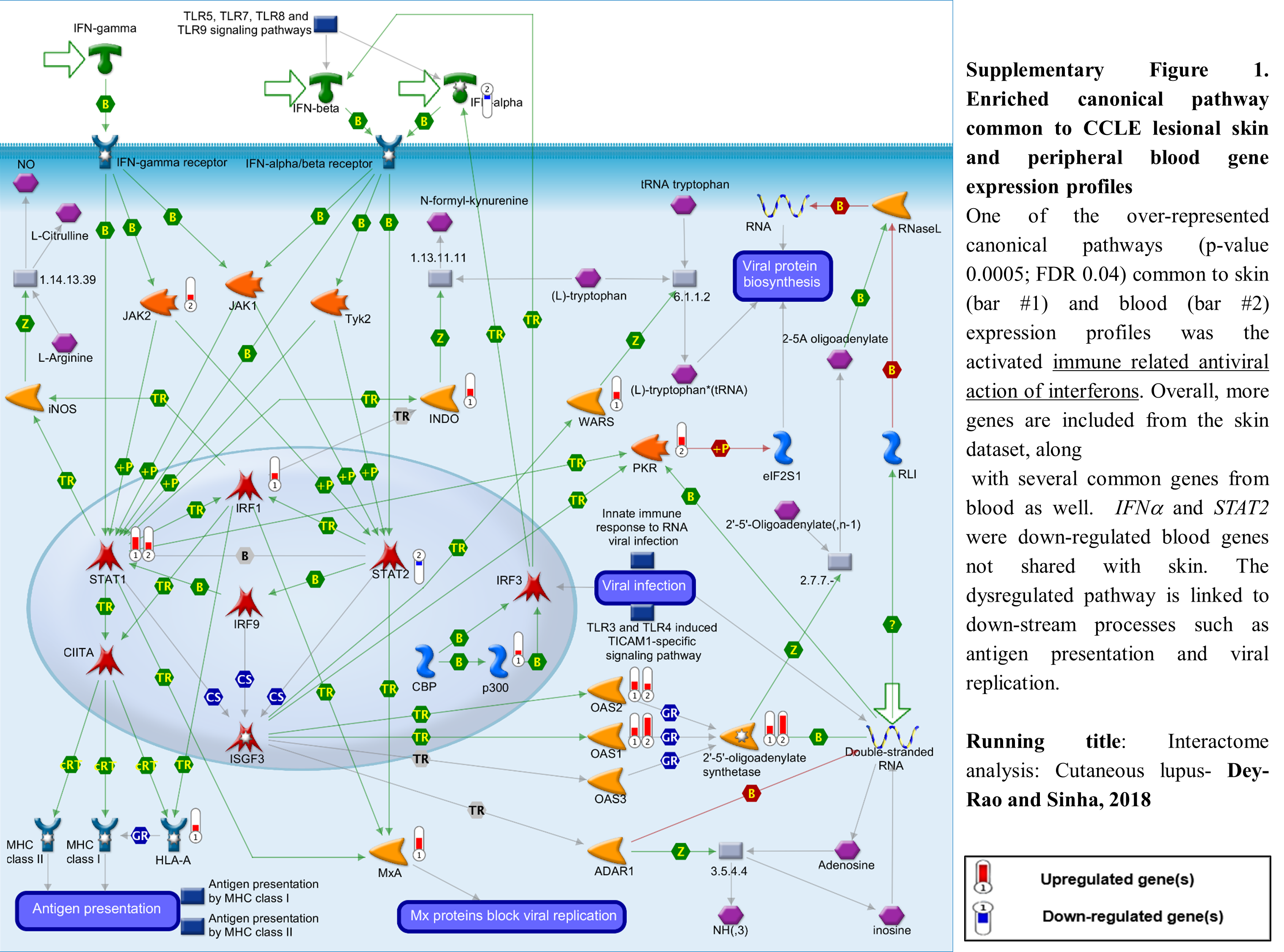

Supplement: Supplementary file 9 [file Image_1.TIF]
